# Comparative Analysis of Small-Molecule LIMK1/2 Inhibitors: Chemical Synthesis, Biochemistry, and Cellular Activity

**DOI:** 10.1021/acs.jmedchem.2c00751

**Published:** 2022-10-07

**Authors:** Ross Collins, Hyunah Lee, D. Heulyn Jones, Jonathan M. Elkins, Jason A. Gillespie, Carys Thomas, Alex G. Baldwin, Kimberley Jones, Loren Waters, Marie Paine, John R. Atack, Simon E. Ward, Olivera Grubisha, David W. Foley

**Affiliations:** † Medicines Discovery Institute, School of Biosciences, Cardiff University, Cardiff CF10 3AT, United Kingdom; ‡ Centre for Medicines Discovery, University of Oxford, Old Road Campus, Roosevelt Drive, Oxford OX3 7DQ, United Kingdom

## Abstract

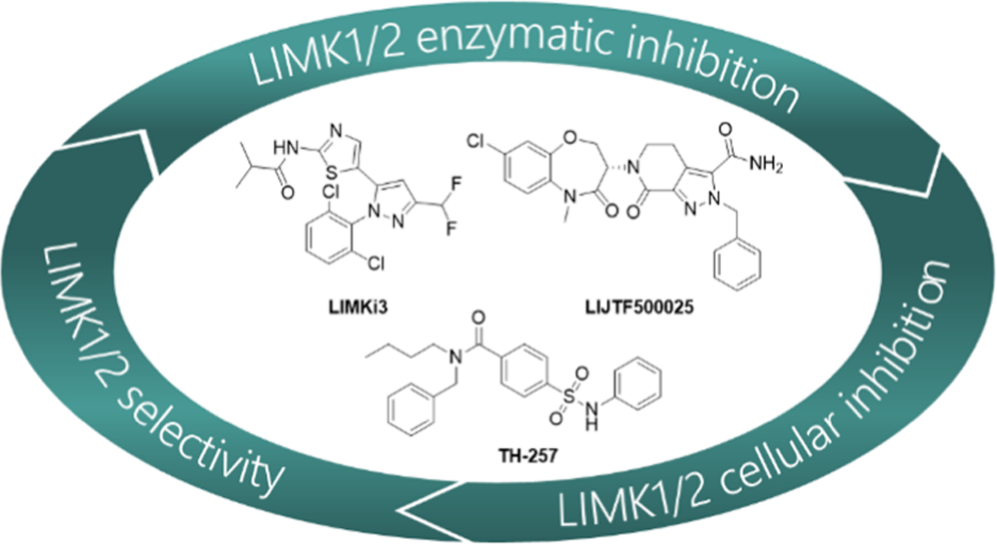

LIM domain kinases 1 and 2 (LIMK1 and LIMK2) regulate actin dynamics and subsequently key cellular functions such as proliferation and migration. LIMK1 and LIMK2 phosphorylate and inactivate cofilin leading to increased actin polymerization. As a result, LIMK inhibitors are emerging as a promising treatment strategy for certain cancers and neurological disorders. High-quality chemical probes are required if the role of these kinases in health and disease is to be understood. To that end, we report the results of a comparative assessment of 17 reported LIMK1/2 inhibitors in a variety of *in vitro* enzymatic and cellular assays. Our evaluation has identified three compounds (TH-257, LIJTF500025, and LIMKi3) as potent and selective inhibitors suitable for use as *in vitro* and *in vivo* pharmacological tools for the study of LIMK function in cell biology.

## Introduction

LIMK1 and LIMK2 are dual-specificity serine/threonine and tyrosine protein kinases involved in actin cytoskeletal dynamics.^1,2^ Both enzymes share the primary function of phosphorylating, and thereby inactivating, the actin depolymerizing factor (ADF)/cofilin family of proteins.^3−5^ This alters the cellular ratio between filamentous (F) and globular (G) actin, which, in turn, regulates processes such as cell motility, proliferation, and migration, as well as synapse stability. The ability to pharmacologically alter these important cellular processes could have implications for a wide variety of diseases, and, indeed, the inhibition of LIMKs has been proposed as a therapeutic strategy for various cancers,^6−15^ glaucoma,^16^ and CNS diseases.^17−19^

It is unsurprising, given the emerging roles of LIMK1 and LIMK2 in diseases, that the number of reports of small-molecule LIMK inhibitors is steadily increasing. A diverse range of assay formats were used to assess the potency of both enzymatic and cellular inhibitions of the reported compounds, and in many instances, limited or no LIMK selectivity and/or cellular data was reported. To fully understand the role of LIMKs in these diseases, well-characterized chemical probes are an essential requirement.^20,21^

As part of our ongoing drug discovery efforts around selective LIMK1 inhibitors for the treatment of Fragile X Syndrome (FXS),^18^ we profiled reported LIMK inhibitors in a series of assays to assess their potency and selectivity in both enzymatic (RapidFire mass spectrometry IC_50_ assay assessing cofilin phosphorylation by the kinase catalytic domain) and cellular models (NanoBRET IC_50_ intracellular kinase assay and AlphaLISA IC_50_ assay measuring phospho-cofilin). *In vitro* and *in vivo* DMPK parameters were generated for the most promising compounds. We share these data with the community, alongside our recommendations for the most suitable currently available LIMK inhibitors to accelerate and support drug development against these proteins.

**Figure 1:**
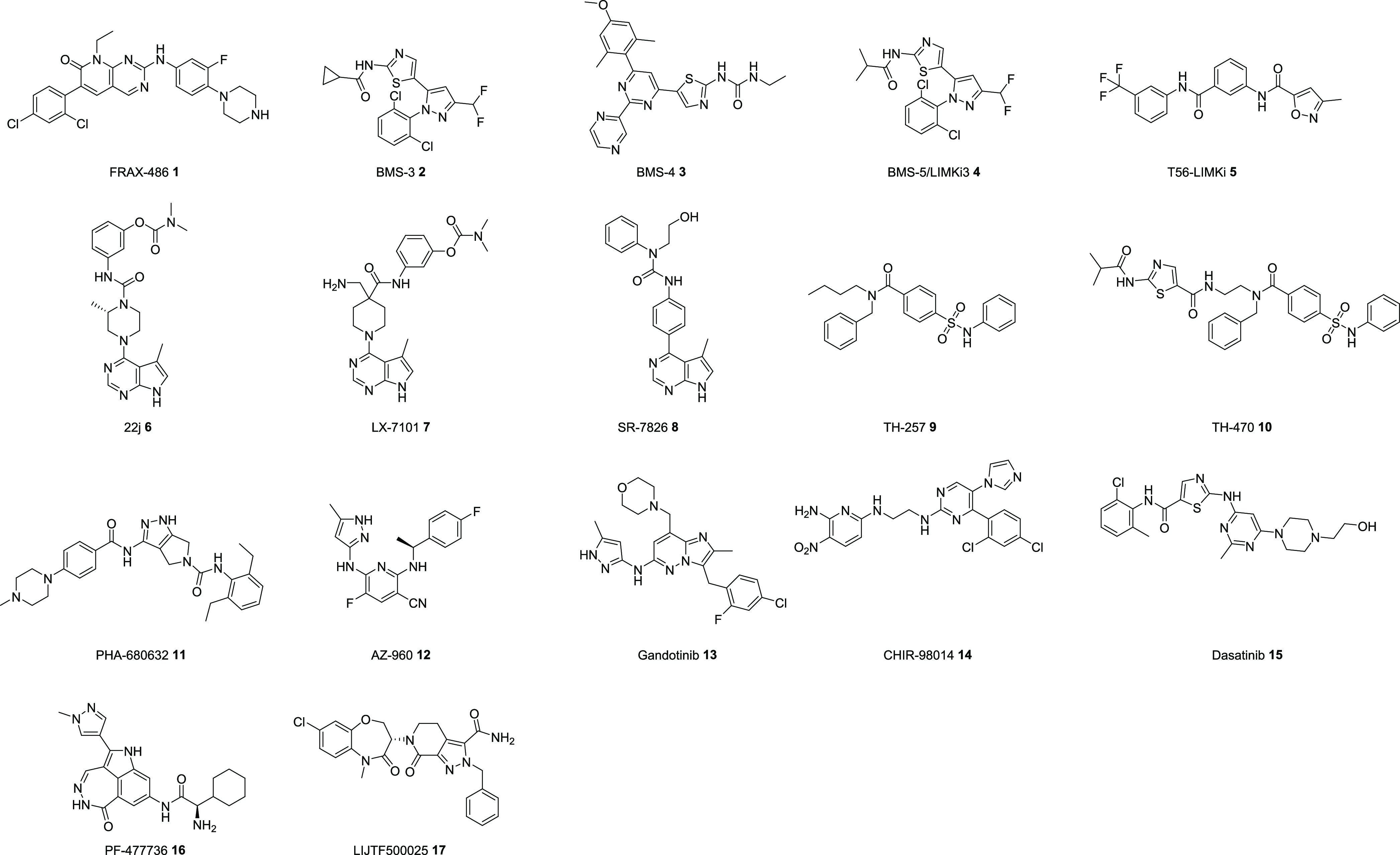
Structures of reported LIMK inhibitors evaluated in this study.

We chose to profile a selection of 17 inhibitors, including examples of some of the most widely used LIMK inhibitors from the literature (BMS-3, **2**, BMS-4, **3**, and LIMKi3, **4**);^22,23^ examples of clinical candidates (LX7101, **7**, a topical LIMK inhibitor for glaucoma);^24^ bis-aryl urea inhibitors, **8;**(25,26) reported examples of ATP-competitive inhibitors with selectivity between LIMK1 and 2 (T56-LIMKi, **5**,^27^ PHA-680632, **11**,^28^ AZ960, **12**,^28^ and gandotinib, **13**(28)); approved drugs (dasatinib, **15**);^28^ and examples of inhibitors that do not bind at the hinge region of the kinases (TH-257, **9**(29,30) and LIJTF500025, **17**,^31,32^) along with the hinge fusion analogue of TH-257 (TH-470, **10**),^29,30^ first discovered by Knapp and colleagues.^29−32^ The structures of all compounds chosen for the study are given in Figure 1. FRAX486 (**1**), a potent PAK inhibitor and a clinical candidate for FXS, was chosen as a positive control for measuring cellular phospho-cofilin (*p*-cofilin) levels in our alphaLISA assay (*vide infra*).^33^

## Results and Discussion

### Chemistry

**Scheme 1:**
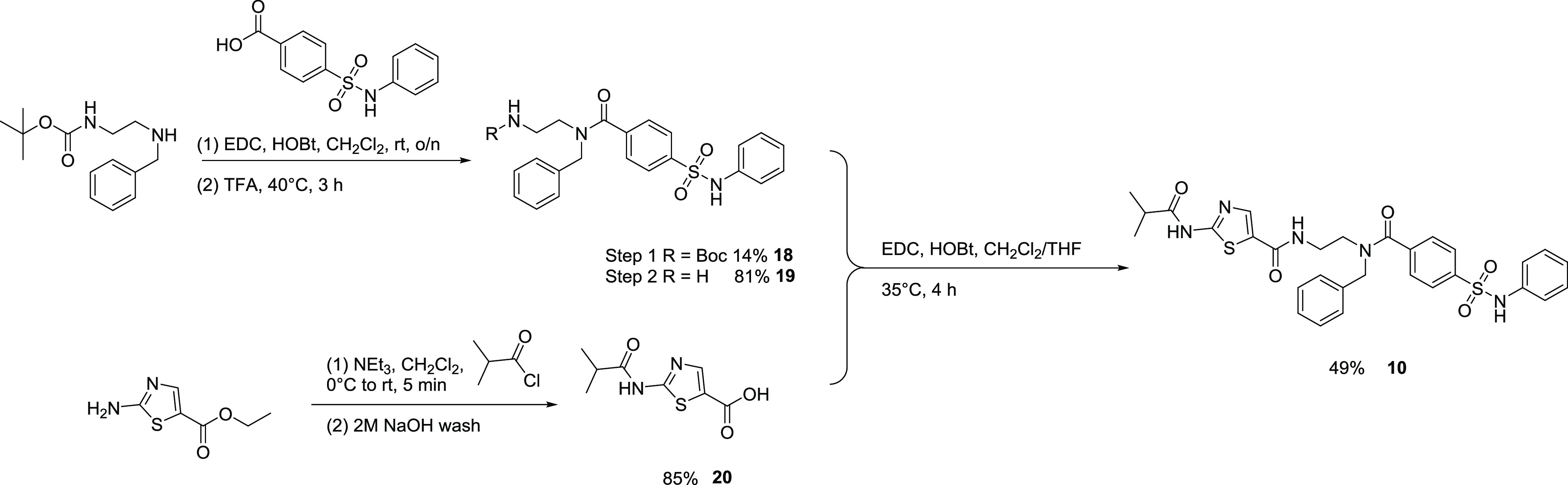
Synthesis of TH-470 (**10**)

The compounds reported in this study were purchased, where possible, from commercial suppliers, and quality control was carried out using ^1^H NMR spectroscopy and ultraperformance liquid chromatography–mass spectrometry (UPLC–MS) analysis (Supporting Information). Where synthesis was required, this was carried out according to the literature procedure with minor modifications, as detailed in the Supporting Information. The synthesis of TH-470 (**10**) has not been reported to date, so it was carried out using a five-step procedure from commercial building blocks (Scheme 1). *N*-Benzyl-*N*′-boc-ethylenediamine was coupled with 4-(phenylsulfamoyl)benzoic acid using the EDC/HOBt coupling reagents. Removal of the Boc-protected intermediate (**18**) with TFA furnished the desired amine (**19**). 2-(2-Methylpropanoylamino)thiazole-5-carboxylic acid (**20**) was prepared in high yield by the reaction of ethyl 2-aminothiazole-5-carboxylate with 2-methylpropanoyl chloride followed by sodium hydroxide-mediated ester hydrolysis. Finally, **19** and **20** were coupled using EDC/HOBt to give TH-470 (**10**) in moderate yield.

### Biological Evaluation

To address some of the limitations of the existing data, including missing enzymatic and cellular activity data and varying accounts for selectivity within the LIMK family, we chose to evaluate the compounds in a series of enzymatic and cellular inhibition assays against both LIMK1 and LIMK2, using a slightly modified version of a previously reported RapidFire mass spectrometry assay.^28^ Additionally, because LIMK1/2 is activated by phosphorylation (on Thr508/Thr505) by p21-activated kinases (PAKs), we conducted our enzymatic inhibition assay in both the presence and absence of PAK1 kinase domain. To assess cellular activity and selectivity, we employed separate LIMK1 and LIMK2 NanoBRET assays in HEK293 cells. One limitation of both our assay formats is that they use modified LIMK proteins (RapidFire—catalytic domain; NanoBRET—NanoLuc fusion). To study the effect of inhibitors in more biologically relevant conditions, we measured the impact of the compounds on *p*-cofilin levels in SH-SY5Y cells using an AlphaLISA platform, although this assay does not discriminate between LIMK1 and/or LIMK2 inhibition since cofilin is a substrate for both enzymes. The results of these studies are presented in Table 1.

**Table 1:** Comparative Evaluation of Enzymatic and Cellular Activities of Reported LIMK Inhibitors

	RapidFire pIC_50_^a^				NanoBRET pIC_50_^a^		AlphaLISA pIC_50_^a^
compound	LIMK1^b^	LIMK2^c^	PAK pLIMK1	PAK pLIMK2	LIMK1	LIMK2	*p*-cofilin
**1**	8.13 ± 0.05	7.87 ± 0.13	6.61 ± 0.05	6.29 ± 0.14	7.16 ± 0.04	7.41 ± 0.05	7.50 ± 0.13
**2**	7.75 ± 0.04	7.52 ± 0.04	7.00 ± 0.03	6.56 ± 0.06	7.09 ± 0.08	7.79 ± 0.02	7.40 ± 0.32
**3**	7.25 ± 0.06	6.87 ± 0.10	5.64 ± 0.06	6.15 ± 0.04	6.45 ± 0.14	6.33 ± 0.12	6.34 ± 0.05
**4**	8.19 ± 0.05	7.48 ± 0.03	7.15 ± 0.07	6.47 ± 0.10	7.12 ± 0.19	7.59 ± 0.28	7.36 ± 0.09
**5**	<5	<5	<5	<5	<5	<5	<5^d^
**6**	8.70 ± 0.07	8.74 ± 0.12	7.68 ± 0.04	7.68 ± 0.07	8.28 ± 0.08	8.61 ± 0.04	8.52 ± 0.13
**7**	7.91 ± 0.04	7.97 ± 0.08	6.53 ± 0.05	6.51 ± 0.04	7.63 ± 0.03	8.17^d^	7.05 ± 0.17
**8**	6.43 ± 0.36	5.72 ± 0.27	5.69 ± 0.19	<5	6.13 ± 0.15	6.26 ± 0.09	6.33 ± 0.20
**9**	6.70 ± 0.10	7.84 ± 0.05	6.71 ± 0.13	7.81 ± 0.03	6.64 ± 0.08	6.72 ± 0.07	6.59 ± 0.07
**10**	8.19 ± 0.05	8.26 ± 0.01	8.84 ± 0.09	8.15 ± 0.04	7.59 ± 0.20	7.57 ± 0.17	6.19 ± 0.17
**11**	6.39 ± 0.03	5.10 ± 0.04	6.24 ± 0.04	<5	<5	<5	<5
**12**	6.82 ± 0.06	5.71 ± 0.14	6.52 ± 0.01	5.18 ± 0.03	<5	<5	<5^d^
**13**	6.40 ± 0.03	<5	5.98 ± 0.06	5.22 ± 0.05	<5	<5	<5
**14**	<5	<5	<5	<5	<5	<5	<5
**15**	7.23 ± 0.02	6.65 ± 0.09	5.99 ± 0.06	5.82 ± 0.06	5.75 ± 0.04	6.58 ± 0.15	7.13 ± 0.09
**16**	6.09 ± 0.07	<5	6.76 ± 0.12	5.47 ± 0.04	6.40 ± 0.04	6.56 ± 0.02	<5
**17**	6.1^e^	8.2^e^	5.5^e^	8.1^e^	6.77 ± 0.13	7.03 ± 0.07	7.04 ± 0.06

a Data are reported as mean ± SEM of at least three independent experiments unless otherwise stated.

b Enzyme assay concentration of 40 nM means pIC_50_ greater than 7.7 (<20 nM) should be treated with caution.

c Enzyme assay concentration of 15 nM means pIC_50_ greater than 8.1 (<7.5 nM) should be treated with caution.

d Mean of two independent experiments.

e Representative data of one experiment only.

### Results

Initial assessment of reported inhibitors in our RapidFire assays yielded some interesting results. As expected, most reported inhibitors showed activity and high potency in these assays. One exception was T56-LIMKi (**5**), which has been reported in the literature as a selective LIMK2 inhibitor,^27^ even being administered to some animal models of diseases.^34^ However, evidence for this observation was limited to Western blot experiments and no direct evidence of this compound inhibiting these kinases has been presented to date. Our results show that T56-LIMKi has no inhibitory activity against either LIMK1 or 2 (or their PAK-phosphorylated forms), nor does it show any cellular activity against either enzyme in the NanoBRET assay. T56-LIMKi also failed to influence *p*-cofilin levels in SH-SY5Y cells using our AlphaLISA assay. Collectively, these data strongly suggest that, in our laboratories, T56-LIMKi is not an inhibitor of these kinases and should not be employed as a tool for the study of these enzymes.

Several ATP-competitive inhibitors designed to bind the kinase hinge region showed a clear loss of potency *in vitro* when phosphorylated pLIMK1/2 was used instead of unmodified LIMK1/2. This included key tool compounds such as BMS-3 (**2**) and BMS-4 (**3**) as well as clinical LIMK inhibitors such as LX7101 (**7**). Numerous studies have shown significant levels of pLIMK1/2 in cells and tissues,^35−37^ and the phosphorylated form of LIMK has significantly greater enzymatic activity; however, the relevance of this observation for drug discovery remains an open question as LIMK inhibitors could prevent the activation of LIMK in cells, in which case *in vitro* inhibition of the unphosphorylated LIMK would be more predictive of the cellular effects. Data on a wider range of inhibitors will be required to adequately assess the correlation between the inhibition of LIMK or pLIMK and cellular effect.

In our RapidFire assay, we were able to use 5 nM LIMK1 + 0.4 nM PAK1 or 6 nM LIMK2 + 0.2 nM PAK1 to achieve a similar cofilin phosphorylation rate as 40 nM LIMK1 or 15 nM LIMK2, respectively, demonstrating the effect on the LIMK activity of PAK activation. In contrast to the hinge binding compounds, phosphorylation of LIMK1/2 did not appreciably change the potencies of the allosteric inhibitor **9**. To confirm this observation, five structurally similar reported allosteric inhibitors (**21**–**24**) were synthesized using modified literature procedures^29^ (Supporting Information), which showed the same trend (Table 2), leading us to conclude that these allosteric inhibitors are less affected by the PAK-mediated phosphorylation state of LIMK. A structurally distinct allosteric inhibitor (**17**) was also unaffected by phosphorylation status, further strengthening this observation.

**Table 2:**
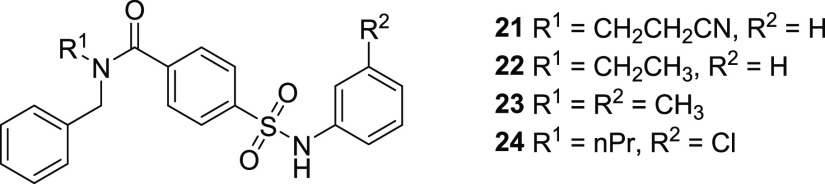
LIMK and pLIMK1/2 Inhibition by Reported Nonhinge Binding Compounds^a^

	RapidFire pIC_50_			
compound	LIMK1	LIMK2	PAK pLIMK1	PAK pLIMK2
**9**	6.70 ± 0.10	7.84 ± 0.05	6.71 ± 0.13	7.81 ± 0.03
**21**	6.45 ± 0.04	8.14 ± 0.06	6.84 ± 0.05	8.05 ± 0.04
**22**	6.77 ± 0.14	7.90 ± 0.04	7.25 ± 0.06	8.19 ± 0.05
**23**	5.75 ± 0.12	6.57 ± 0.01	5.96 ± 0.06	6.70 ± 0.11
**24**	6.24 ± 0.06	7.40 ± 0.07	6.20 ± 0.10	7.30 ± 0.04

a Data are reported as mean ± SEM of at least three independent experiments.

Compounds with a reported difference in the ability to stabilize LIMK1 and LIMK2 against thermal unfolding (change in melting temperature, the *T*_M_) such as PHA-680632 (**11**), AZ960 (**12**), and gandotinib (**13**) showed only a slight preference at best (∼10-fold) in our hands. None of these molecules had any effect in our cellular assays, which for compounds **11** and **13** is most likely attributed to their poor cell permeability and high efflux (see the Supporting Information). However, compound **12** demonstrates good permeability; therefore, there are likely to be other reasons for its poor cellular potency.

No LIMK2-selective compound was identified, despite the reported selectivity of T56-LIMKi (**5**).^27^

FRAX486 (**1**) was initially chosen as a control in the AlphaLISA, as it was known to strongly decrease cofilin phosphorylation by inhibiting PAK kinases,^33^ which lie upstream of LIMK1/2. As expected, FRAX486 led to a decrease in *p*-cofilin levels; however, to our surprise, it also potently inhibited LIMK1/2 in both RapidFire and NanoBRET assays. FRAX486 is a widely used tool compound in Fragile X Syndrome studies, and it is therefore of interest to note that it may derive some of its efficacy for synergistic or additive effects of both PAK and LIMK inhibitions.

The most cellularly potent molecules (**1**, **4**, **7**, **8**, **10**) were further evaluated for their suitability as *in vitro* and *in vivo* tools by assessing their aqueous solubilities, microsomal clearances, *in vivo* PK (including CNS penetration), and wider kinome profiles *via* the commercially available Eurofins/DiscoverX scanMax panel (which employs the KINOMEscan Technology; details at https://www.eurofinsdiscoveryservices.com/services/in-vitro-assays/kinases/screening-profiling-services/kinomescan-technology/) at a single concentration and comparing molecules by the calculation of selectivity score or S-score, wherein the S50 is calculated by dividing the number of kinases that a compound binds to by at least 50% relative to control at the stated concentration, divided by the stated total number of kinases in the panel. Although compound **6** was the most potent inhibitor in our hands, its reported aqueous instability makes it unsuitable for advanced studies.^24^ As a comparator for kinome selectivity, the nonhinge binding compound **17** was also evaluated. The results are summarized in Table 3.

**Table 3:** *In Vitro* and *In Vivo* DMPK Properties and Kinome Selectivity of Selected Tools

compound	*p*-cofilin pIC_50_	aq. solubility (μM, pH 7)^b^	R/H^a^ Mic. Cl_int_ (μL/min/mg)^b^	IV dose (mg/kg)	Cl_int_ (mL/min/kg)	*V*_D_ (L/kg)	*T*_1/2_ (h)	B/P ratio	kinome S_50_ (#kinases, conc)^d^
**1**	7.50 ± 0.13	11	37/6	1	48.8	23.1	6.5	1.35^e^	0.54 (468, 1000 nM)
**4**	7.36 ± 0.09	4	157/4	0.2^c^	30.7	2.0	1.1	1.85^f^	nd
**7**	7.05 ± 0.17	451	50/3	0.5	98.1	6.4	1.3	nd	0.13 (468, 300 nM)
**8**	6.33 ± 0.20	28	18/8	1	4.2	0.5	2.2	0.02^e^	0.07 (468, 1000 nM)
**9**	6.59 ± 0.07	17	>500/450	nd	nd	nd	nd	nd	nd
**10**	6.19 ± 0.17	14	503/356	nd	nd	nd	nd	nd	nd
**17**	7.04 ± 0.06	nd	19/23	1	18.8	1.7	1.3	nd	0.01 (468, 1,000 nM)

a Rat and human microsomes.

b Average of two separate experiments.

c Dosed as a cassette of five compounds.

d S_50_ is the number of compounds that bind/inhibit a kinase >50% at the stated concentration, divided by the stated total number of kinases in the panel.

e Determined at Pharmidex (U.K.).

f Determined internally after IP dosing at 3 mg/kg. nd, not determined.

The allosteric compound **9**, developed by Knapp and co-workers, demonstrated good biochemical and cellular potencies, making it a promising *in vitro* tool for the study of LIMK biology. However, its extremely rapid *in vitro* clearance renders it unsuitable as a potential *in vivo* tool. Its ATP-fusion TH-470 (**10**), also developed by the Knapp laboratory, does not offer improved potency or *in vivo* potential.

LX7101 (**7**) is the optimized analogue of (**6**) with improved stability.^24^ It was developed for ocular administration wherein it reached phase 1 clinical trial [NCT01528111], where it showed efficacy in lowering intraocular pressure. However, its rapid *in vivo* clearance along with its lack of kinome selectivity makes it less useful as a general *in vivo* and *in vitro* tool. Both FRAX486 (**1**) and SR7826 (**8**) have shown efficacy *in vivo* for CNS diseases. Indeed, FRAX486 was found to be a highly brain-penetrant molecule (B/P = 1.35), which supports its use as a tool compound for both systemic and CNS indications. FRAX486, however, is a dual LIMK/PAK inhibitor whose clinical development was also halted due to toxicity and therefore is not recommended as a tool compound as any results could not be unambiguously attributed to LIMK or PAK inhibition. However, it is an extremely potent inhibitor of *p*-cofilin production and could be exploited as a positive control in these assays. Compound **8** shows sufficient promiscuity across the kinome to limit its usefulness as an LIMK investigative tool. In addition, despite a recent study demonstrating *in vivo* efficacy in a model of Alzheimer’s disease,^36^ SR7826 also shows poor brain penetration (B/P = 0.02), limiting its effectiveness for CNS applications and would be more appropriate for systemic indications.

LIMKi3 (**4**) demonstrates good biochemical and cellular potencies and has suitable *in vitro* and *in vivo* PK properties. LIMKi3 has excellent brain penetration (B/P = 1.85), making this compound the best pharmacological inhibitor of LIMK for CNS diseases in our hands. However, it is worth highlighting that in numerous cancer studies, *in vitro* concentrations of **4** of up to 5 μM have been required to see a phenotypic response, which does not align with its *in vitro* potency in our hands. The CaCo-2 permeability of this compound (*P*_app_ (A – B)/(B – A) = 8.8/12.4 × 10^–6^ cm/s) does not suggest a permeability problem, suggesting perhaps that the cancers studied are not susceptible to LIMK inhibition. To reduce the risk of off-target kinome activity even further, the recently described allosteric inhibitor (**17**) also shows excellent kinome selectivity combined with the potency and a suitable *in vivo* DMPK profile for use as both an *in vitro* and *in vivo* tool.

### Conclusions

We set out to evaluate the most commonly used inhibitors of LIMK1 and 2. Our study revealed several key findings:

(1) PAK phosphorylation of LIMK1/2 consistently decreases the in vitro potency of ATP-competitive LIMK1/2 inhibitors.
(2) Allosteric LIMK1/2 inhibitors are not appreciably affected by the PAK phosphorylation of LIMK1/2. The above two points are of general interest to other researchers attempting to develop ATP-competitive LIMK inhibitors, in particular.
(3) FRAX486 (**1**), a PAK inhibitor and preclinical candidate for FXS therapy, also strongly inhibited LIMK1/2. A portion of its efficacy may therefore be due to the dual inhibition of the RAK–PAK–LIMK pathway.
(4) The claimed LIMK2-selective inhibitor, T56-LIMKi (**5**), was inactive in all of our assays.

The key aim of our study was to evaluate reported inhibitors of LIMK in a panel of assays and compare them head to head to enable recommendations for researchers in the field. The allosteric inhibitor LIJTF500025 (**17**) is, as expected by its allosteric mode of inhibition, by far the most selective LIMK inhibitor while retaining reasonable *in vitro* enzymatic and cellular activities and as such is our recommendation as the *in vitro* probe of choice for the study of LIMK biology. TH-257 is a potent and likely selective inhibitor (due to its allosteric mode of action), but its rapid *in vitro* clearance makes it unsuitable for use as an *in vivo* tool; however, LIJTF500025 does have a suitable IV rat DMPK profile to be considered for such use. In addition, the ATP-competitive inhibitor LIMKi3 (**4**) also shows a promising DMPK profile for use as an *in vivo* probe, particularly for exploring the effect of LIMK inhibition on CNS indications.

The availability of well-characterized, selective molecules with orthogonal inhibition modes that are suitable for *in vivo* use can only be of benefit to researchers wishing to study the role of LIMKs in health and diseases.

## Experimental Section

### General Methods

All commercial materials were used as received without further purification. Identity and purity checks were carried out prior to use in biological experiments using ^1^H NMR spectroscopy and UPLC–MS analysis on the instruments detailed below and is included in the Supporting Information for reference. Analytical thin-layer chromatography was conducted using aluminum-backed plates coated with a VWR TLC silica gel 60 F254 that were visualized under ultraviolet (UV) light (at 254 nm) or stained using KMnO_4_. Nuclear magnetic resonance (NMR) spectra were recorded on a Bruker Avance III HD 500 MHz equipped with a Prodigy cryoprobe. Chemical shifts were reported in parts per million (ppm) in the scale relative to residual solvent signals. Multiplicities are abbreviated as follows: s, singlet; d, doublet; t, triplet; q, quartet; dd, doublet of doublets; tt, triplet of triplets; pent; pentet; hept, heptet; m, multiplet; br, broad, or combinations thereof. Coupling constants were measured in Hertz (Hz). Liquid chromatography–mass spectrometry (LCMS) data were recorded on a Waters Acquity H-class plus UPLC coupled to a Waters Acquity UPLC PDA detector and a Waters Acquity QDa API-ES mass detector. Samples were eluted through a BEH C18 2.1 mm × 50 mm, 1.7 μm column or a Cortecs C_18_ 2.1 mm × 50 mm, 1.6 μm column using water and acetonitrile acidified by 0.1% formic acid. The gradient runs H_2_O/MeCN/formic acid at 90:10:0.1–10:90:0.1 for 3 min at 1.5 mL/min and detected at 254 nm. Normal-phase purifications were completed using a Teledyne ISCO CombiFlash NEXTGEN 300+; reverse-phase purifications were completed using a Teledyne ACCQPrep system equipped with a 20 mm × 150 mm C_18_ column and eluted with a 10–100% MeOH/water gradient. All compounds were determined to be >95% pure, as determined by ^1^H NMR and UPLC analyses.

#### *tert*-Butyl *N*-[2-[Benzyl-[4-(phenylsulfamoyl)benzoyl]amino]ethyl]carbamate (**18**)

A mixture of 4-(phenylsulfamoyl)benzoic acid (627 mg, 2.26 mmol), *N*-(3-dimethylaminopropyl)-*N*′-ethylcarbodiimide hydrochloride (520 mg, 2.71 mmol), and hydroxybenzotriazole hydrate (381 mg, 2.49 mmol) in CH_2_Cl_2_ (35 mL) was stirred at rt for 1 h before the addition of *N*-benzyl-*N*′-boc-ethylenediamine (849 mg, 3.39 mmol) in CH_2_Cl_2_ (5 mL). The vial was sealed, and the mixture was stirred at rt overnight. The reaction mixture was concentrated under reduced pressure, and the residue was purified by automated flash column chromatography (silica, 24 g, CH_2_Cl_2_/MeOH; 100:0–98:2 over 30 CV). The appropriate fractions were combined, and the solvent was removed to give *tert*-butyl *N*-[2-[benzyl-[4-(phenylsulfamoyl)benzoyl]amino]ethyl]carbamate as a colorless solid (**18**, 168 mg, 0.313 mmol, 14% yield).

^1^H NMR (500 MHz, chloroform-*d*) δ 7.78 (d, *J* = 7.8 Hz, 0.6H), 7.72 (d, *J* = 8.0 Hz, 1.4H), 7.47 (d, *J* = 7.8 Hz, 2.0H), 7.40–7.27 (m, 3.7H), 7.24–7.18 (m, 2.0H), 7.12 (t, *J* = 7.5 Hz, 1H), 7.07 (d, *J* = 7.4 Hz, 1.9), 7.03 (d, *J* = 7.9 Hz, 1.5H), 6.65–6.53 (m, 0.7H), 4.96 (s, 0.6H), 4.80 (s, 0.6H), 4.45 (s, 1.4H), 4.31 (s, 0.2H), 3.65–3.57 (m, 1.4H), 3.45–3.38 (m, 1.3H), 3.20–3.09 (m, 1.1H), 1.45–1.40 (m, 9.0H). Rotamers were observed in a 1:0.43 ratio, and substantial broadening of peaks was observed. ACQUITY UPLC BEH C_18_ 1.7 μm: *R*_t_ = 1.79 min; *m*/*z* 508.1 [M – H]^−^.

#### *N*-(2-Aminoethyl)-*N*-benzyl-4-(phenylsulfamoyl)benzamide (**19**)

To a solution of *tert*-butyl-*N*-[2-[benzyl-[4-(phenylsulfamoyl)benzoyl]amino]ethyl]carbamate (168 mg, 0.330 mmol) in CH_2_Cl_2_ (2.5 mL) was added trifluoro acetic acid (1.5 mL, 19.59 mmol), and the mixture was stirred at 40 °C for 3 h. LCMS analysis confirmed reaction completion. The reaction mixture was concentrated under reduced pressure in a fumehood, and the resulting residue was dissolved in MeOH/DMSO and passed over a 2 g SCX-2 column, releasing the captured free amine with 0.7 M ammonia in MeOH. The amine was dried under reduced pressure and further dried in a vacuum oven at 40 °C for 48 h to afford the product *N*-(2-aminoethyl)-*N*-benzyl-4-(phenylsulfamoyl)benzamide (**19**) as a colorless solid (115 mg, 0.267 mmol, 81% yield).

^1^H NMR (400 MHz, DMSO-*d*_6_) δ 7.79 (d, *J* = 7.9 Hz, 1.1H), 7.73 (d, *J* = 8.0 Hz, 0.9H), 7.59 (d, *J* = 7.8 Hz, 1.1H), 7.54 (d, *J* = 7.8 Hz, 0.9H), 7.41–7.24 (m, 3.9H), 7.23–7.12 (m, 2.1H), 7.12–6.90 (m, 4.0H), 4.69 (s, 1.1H), 4.40 (s, 0.9H), 3.04 (t, *J* = 6.8 Hz, 1.3H), 2.79 (t, *J* = 8.5 Hz, 0.8H), 2.57 (t, *J* = 7.0 Hz, 1H). Rotamers were observed in a 1:0.8 ratio; one multiplet is underneath a broad water signal (not reported). ACQUITY UPLC BEH C_18_ 1.7 μm: *R*_t_ = 1.55 min; *m*/*z* 410.1 [M + H]^+^.

#### 2-(2-Methylpropanoylamino)thiazole-5-carboxylic Acid Hydrochloride (**20**)

To an ice-cold stirring solution of triethylamine (0.97 mL, 6.97 mmol) and ethyl 2-aminothiazole-5-carboxylate (1.00 g, 5.81 mmol) in CH_2_Cl_2_ (20 mL) was added 2-methylpropanoyl chloride (0.61 mL, 5.81 mmol) drop-wise. Upon the completion of addition, the mixture was left to stir at rt for 5 min, after which LCMS analysis indicated reaction completion. The reaction mixture was diluted with CH_2_Cl_2_ (20 mL) and washed sequentially with water (20 mL), 1 M HCl (aq) (2 × 30 mL) and 2 M NaOH (2 × 30 mL). LCMS analysis of the basic aqueous phase indicated the presence of the hydrolyzed ester; therefore, the pH was made acidic (pH 4) and extracted with EtOAc (3 × 20 mL). The organic phase was dried over magnesium sulfate, filtered, and concentrated under reduced pressure to give the desired acid (223 mg). LCMS analysis of the remaining aqueous phase indicated that a substantial amount of the desired acid was present. The aqueous phase was concentrated under reduced pressure, redissolved in water (10 mL), and made acidic (pH 4). An attempt was made to extract using EtOAc; however, a suspension formed in the organic phase and so the mixture was filtered. The solid collected was left to air-dry overnight and then collected and dried more thoroughly in a vacuum oven (40°C) for 3 h to give a second batch of the desired acid. Both batches were combined to give 2-(2-methylpropanoylamino)thiazole-5-carboxylic acid as the presumed acid hydrochloride salt, isolated as a beige solid (**20**, 1.296 g, 4.911 mmol, 85% yield).

^1^H NMR (500 MHz, DMSO-*d*_6_) δ 13.06 (s, 1H), 12.45 (s, 1H), 8.04 (s, 1H), 2.76 (sept, *J* = 6.9 Hz, 1H), 1.12 (d, *J* = 6.9 Hz, 6H). ACQUITY UPLC BEH C18 1.7 μm: *R*_t_ = 1.24 min; *m*/*z* 215.0 [M + H]^+^.

#### *N*-[2-[Benzyl-[4-(phenylsulfamoyl)benzoyl]amino]ethyl]-2-(2-methylpropanoylamino)thiazole-5-carboxamide (**10**)

A mixture of *N*-(2-aminoethyl)-*N*-benzyl-4-(phenylsulfamoyl)benzamide (**19**, 100 mg, 0.240 mmol), 2-(2-methylpropanoylamino)thiazole-5-carboxylic acid (**20**, 134.4 mg, 0.370 mmol), *N*-(3-dimethylaminopropyl)-*N*′-ethylcarbodiimide hydrochloride (56.2 mg, 0.290 mmol), and 1-hydroxybenzotriazole hydrate (52.2 mg, 0.290 mmol) in CH_2_Cl_2_ (10 mL) and THF (2 mL) was stirred at 35°C for 4 h under an inert atmosphere. Heating was ceased, and the reaction mixture was concentrated under reduced pressure to give a residue, which was suspended in methanol (7.5 mL). The suspension was filtered through cotton wool, and the filtrate was subjected to automatic reverse-phase column chromatography (20 mm × 150 mm Prep. HPLC column, MeOH/H_2_O; 10:90–100:0 for 25 min) to afford *N*-[2-[benzyl-[4-(phenylsulfamoyl)benzoyl]amino]ethyl]-2-(2-methylpropanoylamino)thiazole-5-carboxamide (**10**) as an off-white solid (77 mg, 0.121 mmol, 49% yield).

^1^H NMR (500 MHz, methanol-*d*_4_)^38^ δ 7.94 (s, 0.4H), 7.86 (s, 0.3H), 7.73 (d, *J* = 7.9 Hz, 1.7H), 7.47–7.42 (m, 1.8H), 7.41–7.33 (m, 1.5H), 7.33–7.22 (m, 1.9H), 7.22–7.17 (m, 0.7H), 7.17–7.11 (m, 1.1H), 7.11–6.97 (m, 3.7H), 4.46 (s, 1.0H), 3.72–3.63 (m, 2.1H), 3.38 (dd, *J* = 9.7, 4.6 Hz, 1.3H), 3.36–3.34 (m, 0.6H), 2.76 (h, *J* = 7.1 Hz, 1H), 1.23 (d, *J* = 7.2 Hz, 6H). ACQUITY UPLC BEH C18 1.7 μm: *R*_t_ = 1.62 min; *m*/*z* 606.1 [M + H]^+^.

### Cell Lines and Growth Conditions

HEK293 and SH-SY5Y cells used in this study were purchased from Sigma/Merck (Dorset, U.K.). Cells were cultured in Dulbecco’s modified Eagle’s medium (DMEM)/F12 (#11320033, Thermofisher Scientific, U.K.), supplemented with 1% penicillin/streptomycin (Sigma-Aldrich, Dorset, U.K.) and 10% fetal calf serum (Sigma-Aldrich, Dorset, U.K.). Cells were cultured at 37 °C, 5% CO_2_ in a humidified incubator.

### RapidFire Mass Spectrometry (RF-MS) Kinase Assays

A 50 μL reaction was prepared in 384-well polypropylene plates. First, compounds were transferred in duplicate in a 16-point 2-fold serial dilution in DMSO (maximum inhibitor concentration of 40 μM in the LIMK1/2 assay or 4 μM in the PAK-phosphorylated LIMK1/2 assay) using an ECHO 550 acoustic dispenser (Labcyte). To these plates, 25 μL of 80 nM LIMK1_330–637_ (for a final concentration of 40 nM) or 30 nM LIMK2_347–659_ (final concentration of 15 nM) in assay buffer was dispensed into each well using a COMBI multidrop dispenser and the plates were incubated for 45 min at room temperature. Then, 25 μL of 8 μM CFL1 (for a final concentration of 4 μM) and 4 mM ATP (final concentration 2 mM) in assay buffer was added to each well and incubated for 105 and 180 min for LIMK1 and LIMK2, respectively. The composition of the assay buffer used for LIMK1 was 50 mM tris pH 7.5, 0.1 mM EDTA, 0.1 mM EGTA, 1 mM MgCl_2_, while that of the assay buffer used for LIMK2 was 50 mM HEPES pH 7.5, 0.1 mM EGTA, 1 mM EDTA, and 1 mM MnCl_2_. For the PAK-phosphorylated LIMK1/2 RF-MS assay, PAK1 kinase domain (249–545) was used at a final concentration of 0.4 nM and 0.2 nM along with LIMK1 (5 nM) and LIMK2 (6 nM), respectively.

The LIMK phosphorylation reactions were halted by the addition of 5 uL of 10% formic acid (final concentration of 1%), and the assay plates were transferred onto a RapidFire RF360 instrument (Agilent). Once loaded, the samples were aspirated under vacuum and the salts and the nonvolatile buffer components were removed by loading onto a C4 solid-phase extraction (SPE) cartridge (Agilent Technologies) in 0.1% formic acid in water at a flow rate of 1.5 mL/min. Elution using 85% acetonitrile and 0.1% formic acid was then used to elute analytes into the mass spectrometer (Agilent 6530 QTOF) at a flow rate of 1.2 mL/min. The resulting data were analyzed using RapidFire integrator software (Agilent), and GraphPad Prism 7 was used to calculate IC_50_ values.

### Transient Transfection of HEK293 Cells

A transfection reagent mix was prepared and consisted of 1.25 mL of Opti-MEM without phenol red (Fisher Life Technologies, U.K.), 1.25 μg of NanoLuc LIMK1 or LIMK2 kinase fusion vector (Promega, Hampshire, U.K.), 11.25 μg of transfection carrier DNA (Promega, Hampshire, U.K.), and 37.5 μL of FuGENE HD transfection reagent (Promega, Hampshire, U.K.), according to the manufacturer’s protocol. HEK293 cells were resuspended in growth media following routine trypsinization, neutralization, and sedimentation techniques. Cell density was adjusted to 1 ×10^5^ cells/mL for each transfection in a total of 25 mL media. The transfection mix was added directly to the cells and mixed gently by inversion. The cells were then plated into T-75 tissue culture flasks and incubated for 20 h.

### Cellular NanoBRET LIMK1/2 Assay

Kit components were purchased from Promega (Hampshire, U.K.). Initially, a compound plate of an 8-point serial dilution was set up in DMSO, which was then further diluted to 1:20 in assay media (Opti-MEM, #31985062, Thermofisher). NanoBRET Tracer #10 (27.5 μL) was diluted in 192.5 μL of DMSO for use as a positive control, which was further diluted with 880 μL of tracer dilution buffer. For negative control, 20 μL of DMSO was added to 80 μL of tracer dilution buffer. LIMK1/2-transfected HEK293 cells were trypsinized, centrifuged, and resuspended in 10 mL of Opti-MEM. Extracellular NanoLuc inhibitor was added at 1:10,000 dilution to the cells, after which 85 μL of cells/well were plated into flat-bottomed, white, 96-well plates. For positive control wells, 5 μL of NanoBRET Tracer #10 was added to wells without compound. For negative controls, the DMSO/tracer dilution buffer solution was added to wells without compound. For test compounds, 10 μL of diluted compounds from the intermediate plate were added in duplicate or DMSO (for control wells) at a final concentration of 0.5% DMSO in Opti-MEM. Plates were incubated at 37 °C and 5% CO_2_ for 2 h. Following incubation, plates were removed and allowed to reach RT for 15 min. A solution of Nano-Glo substrate was made consisting of 72 μL of Nano-Glo substrate and 11.93 mL of Opti-MEM (without phenol red) and mixed gently by inversion 5–10 times. Fifty microliters of diluted Nano-Glo substrate was added to each well on the plate, and luminescence was measured using dual emission for the donor at 450 nM and the acceptor at 610 nM on a BMG Pherastar plate reader.

### AlphaLISA SureFire Assay for the Detection of Phospho-Cofilin Ser3

SH-SY5Y cells were plated into a 96-well plate at 20,000 cells/well. The next day, compounds were prepared in an 8-point, 3-fold serial dilution in DMSO and then further diluted to 20-fold in DMEM/F12 with 10% FBS, 1% antibiotics. SH-SY5Y cell media was replaced with fresh DMEM/F12 media containing 10% FBS and 1% antibiotics. Compounds were added to the SH-SY5Y cells in duplicate at a final compound concentration of 10 μM to 3 nM. LIMKi3 (10 μM) was used as a positive control and 0.5% DMSO was used as a negative control. Cells were placed in the incubator for 2 h, after which the media was removed, and the cells were lysed using 50 μL of AlphaLISA 1× lysis buffer (Perkin Elmer) containing protease inhibitor cocktail (Sigma-Aldrich, Dorset, U.K.) and Pierce phosphatase inhibitor cocktail (Thermofisher, U.K.). Cells were placed on a plate shaker at 350 rpm at RT. Ten microliters of total cell lysate was then transferred to a clean, flat-bottom, white 384-well plate. An acceptor bead solution was made consisting of reaction buffer 1, reaction buffer 2, activation buffer, and acceptor beads from the p-Cofilin SureFire Ultra assay kit (Perkin Elmer, Cat# ALSU-PCOF-A500). To each well in the assay, 5 μL of the acceptor mix was added to the wells under dim light; the plate was placed on a plate shaker for 2 min at 450 rpm, centrifuged briefly, and the plate was incubated at RT for 1 h. A donor solution, consisting of dilution buffer and donor beads, was added at 5 μL/well, mixed on a plate shaker, centrifuged briefly, and incubated at RT for 1 h. Finally, the plate was read on a Pherastar reader (BMG Labtech Ltd., Aylesbury, U.K.) using an AlphaLISA cartridge and AlphaLISA plate settings. The AlphaLISA assay was robust and reproducible (*Z*′ = 0.7).

### Microsomal Stability

Five microliters of microsomes (20 mg/mL, Corning BV) diluted in 95 μL of PBS (pH 7.4 with 0.6% MeCN) containing 0.04% of DMSO and 4 μM of compound were incubated with 100 μL of prewarmed 4 mM of NADPH in PBS (final concentrations: 0.5 mg/mL microsomes, 2 μM MDI-62708, 0.02% DMSO, 0.3% MeCN, and 2 mM NADPH). After mixing thoroughly, the *T* = 0 sample (40 μL) was immediately quenched into an 80 μL ice-cold methanol containing a 4 μM internal standard (carbamazepine). Three further samples were quenched in the same way at *T* = 3, 9, and 30 min. Samples were incubated on ice for 30 min before centrifugation at 4700 rpm for 20 min. The supernatant was analyzed *via* LCMS/MS, and compound/carbamazepine peak area ratios were calculated to determine the rate of substrate depletion.

### Thermodynamic Solubility

One to two milligrams of the accurately weighed compound was suspended in 1 mL of PBS (pH 7.0) at 1 mg/mL and incubated (rotating end over end) at room temperature for 24 h. The samples were then centrifuged at >10,000 rpm for 10 min to pellet any remaining solid. The supernatant was then diluted sequentially (1:5, 1:50, 1:500, and 1:5000) in acetonitrile and mixed 1:1 with acetonitrile containing 4 μM of carbamazepine. To prepare the standard, an 8-point, 1:3 dilution curve was prepared in DMSO with a top concentration of 1 mM, which was then diluted to 1:100 in acetonitrile containing 2 μM of carbamazepine. Standards and samples were analyzed *via* LCMS/MS. The compound carbamazepine peak area ratios were calculated, and the test article solubility was determined by interpolation from the standard curve.

### *In Vivo* DMPK

*In vivo* studies were carried out under appropriate licenses at Pharmidex (U.K.), Sygnature Discovery (U.K.), or Sai Life (India) using male Sprague-Dawley rats. Compounds **1** and **7** were formulated as a solution in 20% hydroxypropyl-β-cyclodextrin in normal saline (w/w) at a concentration of 1 and 0.5 mg/mL, respectively, and were administered in a dose volume of 1 mL/kg (final dose = 1 and 0.5 mg/kg i.v., respectively). Compound **8** was formulated as a solution in 60:40 DMA/normal saline (v/v) at a concentration of 1 mg/mL and was administered in a dose volume of 1 mL/kg (final dose = 1 mg/kg i.v). Compound **4** was formulated as a solution in 10% DMSO and 20% Cremophor EL in normal saline (w/w/w) at a concentration of 0.2 mg/mL and was administered as a cassette with four other compounds in a dose volume of 1 mL/kg (final dose = 0.2 mg/kg i.v.). Serial plasma samples were obtained at predetermined time points and then stored at −20 °C. Samples were then thawed, protein-precipitated with acetonitrile followed by LCMS/MS quantification. No adverse effects were noted for the duration of the experiment for all compounds.

## Supporting Information Available

FR095-0029533-O_EUR152-01-p-00001-000-00_scanMAX_Data Report.csv (CSV)
LIMK strings final.csv (CSV)
Molecular formula strings for final compounds; Western blot and AlphaLISA dose–response curve for compound **4**; Caco-2 permeability results for compounds **11**–**13**; ^1^H NMR and UPLC–MS characterization data for all compounds (including the description of chemical synthesis for compounds **7**, **17**, **21**–**24**); and ^1^H NMR spectra and UPLC–MS chromatograms for all compounds (PDF)

## Notes

All data is provided in full in the results section of this paper.

## Abbreviations Used

ATP: adenosine triphosphate
ADF: actin depolymerizing factor
CCR2: CC chemokine receptor 2
CCL2: CC chemokine ligand 2
CCR5: CC chemokine receptor 5
CNS: central nervous system
FXS: fragile X syndrome
HEK293: human embryonic kidney 293
LIMKs: LIM domain kinases
PAK: p21-activated kinase
TLC: thin-layer chromatography
