# Supplementary material for: Comparative Analysis of Small-Molecule LIMK1/2 Inhibitors: Chemical Synthesis, Biochemistry, and Cellular Activity
Source: J Med Chem. 2022 Oct 7;65(20):13705–13. doi: 10.1021/acs.jmedchem.2c00751 (PMC9619402; doi:10.1021/acs.jmedchem.2c00751)
Supplement: Supplementary file 3 — jm2c00751_si_003.pdf [file jm2c00751_si_003.pdf]

Supporting Information

A Comparative Analysis of Small Molecule LIMK1/2 Inhibitors: Chemical Synthesis, Biochemistry and Cellular Activity

Ross Collins[a], Hyunah Lee[b], D. Heulyn Jones[a], Jonathan M. Elkins[b], Jason A. Gillespie[a], Carys Thomas[a], Alex G. Baldwin[a], Kimberley Jones[a], Loren Waters[a], Marie Paine[a], John R. Atack[a], Simon E. Ward[a]*, Olivera Grubisha[a] and David W. Foley[a]

[a]Medicines Discovery Institute, School of Biosciences, Cardiff University, Cardiff, CF10 3AT, United Kingdom

[b]Centre for Medicines Discovery, University of Oxford, Old Road Campus, Roosevelt Drive, Oxford, OX3 7DQ, United Kingdom

Corresponding Author email address: WardS10@cardiff.ac.uk

Contents

Supplementary biology 2

Final compound characterization and details of chemical synthesis 3-15

Copies of ^{1}H NMR and UPLC data for final compounds

FRAX-486 (**1**) 16

BMS-3 (**2**) 17

BMS-4 (**3**) 18

BMS-5/LIMKi3 (**4**) 19

T56-LIMKi (**5**) 20

22j (**6**) 21

LX-7101 (**7**) 22

SR-7826 (**8**) 23

TH-257 (**9**) 24

TH-470 (**10**) 25

PHA-680632 (**11**) 26

AZ-960 (**12**) 27

Gandotinib (**13**) 28

CHIR-98014 (**14**) 29

Dasatinib (**15**) 30

PF-477736 (**16**) 31

LIJTF500025 (**17**) 32

MDI-60021 (**21**) 33

MDI-60018 (**22**) 34

MDI-60052 (**23**) 35

MDI-60047 (**24**) 36

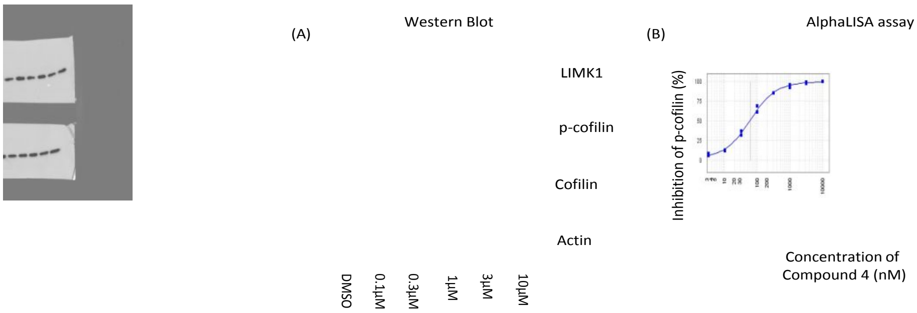

Figure S1 (A) Western blot of SH-SY5Y cell lysates treated with increasing concentrations of compound **4** (0.1µM – 10µM). Effect of compound **4** treatment to LIMK1, p-cofilin and cofilin shown. B-Actin included as a housekeeping protein. Western blot data is representative of n=1. (B) AlphaLISA assay of SH-SY5Y cell lysates treated with increasing concentrations of compound **4** (3nM – 10,000nM). AlphaLISA data is representative of n=9.

Table S1. Caco-2 permeability data for compound **11**-**13**.

Compound	Caco-2 permeability (A->B, x 10^{-6} cm)	Efflux ratio
11	0.10	279
12	19.3	1.01
13	5.04	10.2

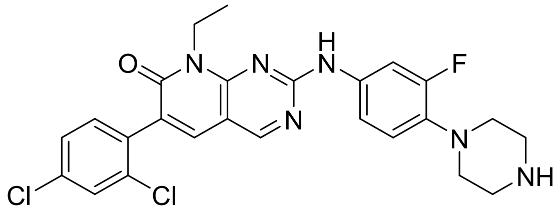

(**1**) FRAX-486. Supplier = Tocris.

^{1}H NMR (500 MHz, DMSO-d_6) δ 10.22 (s, 1H), 8.82 (s, 1H), 7.89 (s, 1H), 7.77 (dd, *J* = 15.2, 2.4 Hz, 1H), 7.72 (d, *J* = 2.1 Hz, 1H), 7.54 – 7.41 (m, 3H), 7.06 – 6.98 (m, 1H), 4.37 (q, *J* = 7.0 Hz, 2H), 2.92 – 2.86 (m, 4H), 2.86 – 2.81 (m, 4H), 1.29 (t, *J* = 7.0 Hz, 3H). ACQUITY UPLC® BEH C18 1.7 µm: Rt = 1.57 min; *m/z* 513.1 [M+H]$^+$.

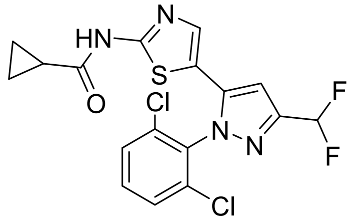

(**2**) BMS-3. Supplier = Sigma Aldrich.

^{1}H NMR (500 MHz, DMSO-d_6) δ 12.60 (s, 1H), 7.82 – 7.77 (m, 2H), 7.76 – 7.69 (m, 2H), 7.23 (s, 1H), 7.07 (t, *J* = 54.2 Hz, 1H), 1.92 – 1.85 (m, 1H), 0.91 – 0.83 (m, 4H). ACQUITY UPLC® BEH C18 1.7 µm: Rt = 1.75 min; *m/z* 429.0 [M+H]$^+$.

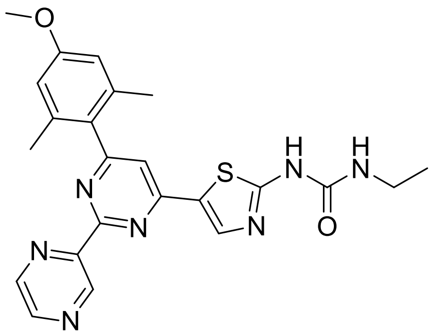

(**3**) BMS-4. Supplier = Axon MedChem.

^{1}H NMR (500 MHz, DMSO-d_6) δ 10.77 (s, 1H), 9.52 (s, 1H), 8.88 – 8.84 (m, 1H), 8.81 (d, *J* = 2.5 Hz, 1H), 8.48 (s, 1H), 8.02 (s, 1H), 6.79 (s, 2H), 6.63 (s, 1H), 3.80 (s, 3H), 3.20 (app p, *J* = 7.2 Hz, 1H), 2.13 (s, 6H), 1.10 (t, *J* = 7.2 Hz, 3H). ACQUITY UPLC® BEH C18 1.7 µm: Rt = 1.65 min; *m/z* 426.2[M+H]$^+$.

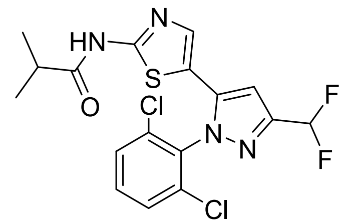

(**4**) LIMKi3, BMS-5. Supplier = Axon MedChem.

^{1}H NMR (500 MHz, DMSO-d_6) δ 12.29 (s, 1H), 7.84 – 7.78 (m, 2H), 7.75 (dd, *J* = 9.2, 7.0 Hz, 1H), 7.72 (s, 1H), 7.24 (s, 1H), 7.07 (t, *J* = 54.4 Hz, 1H), 2.70 (h, *J* = 6.9 Hz, 1H), 1.07 (d, *J* = 6.9 Hz, 6H). ACQUITY UPLC® BEH C18 1.7 µm: Rt = 1.79 min; *m/z* 431.1[M+H]$^+$.

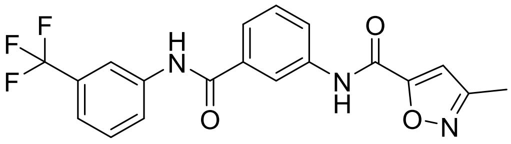

(**5**)T56-LIMKi. Supplier = Sigma Aldrich.

^{1}H NMR (500 MHz, DMSO-d_6) δ 10.91 (s, 1H), 10.61 (s, 1H), 8.33 (t, *J* = 2.0 Hz, 1H), 8.24 (s, 1H), 8.06 (d, *J* = 8.2 Hz, 1H), 8.00 (dd, *J* = 8.2, 2.2 Hz, 1H), 7.76 (d, *J* = 7.7 Hz, 1H), 7.61 (app t, *J* = 8.0 Hz, 1H), 7.56 (app t, *J* = 7.9 Hz, 1H), 7.47 (d, *J* = 7.8 Hz, 1H), 7.15 (s, 1H), 2.35 (s, 3H). ACQUITY UPLC® BEH C18 1.7 µm: Rt = 1.74 min; *m/z* 390.1 [M+H]$^+$.

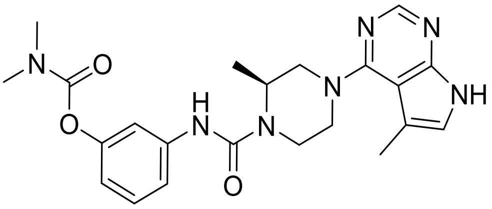

(**6**) 22j. Supplier = Sigma Aldrich.

^{1}H NMR (500 MHz, DMSO-d_6) δ 11.58 (s, 1H), 8.64 (s, 1H), 8.23 (s, 1H), 7.38 – 7.33 (m, 1H), 7.32 – 7.26 (m, 1H), 7.25 – 7.18 (m, 1H), 7.10 (s, 1H), 6.68 (d, *J* = 8.0 Hz, 1H), 4.51 – 4.48 (m, 1H), 4.01 (app t, *J* = 12.0 Hz, 2H), 3.84 (app d, *J* = 12.8 Hz, 1H), 3.46 – 3.34 (m, 1H), 3.26 – 3.20 (m, 1H), 3.05 – 2.95 (m, 4H), 2.90 (s, 3H), 2.40 (s, 3H), 1.18 (d, *J* = 6.6 Hz, 3H). ACQUITY UPLC® BEH C18 1.7 µm: Rt = 1.60 min; *m/z* 438.2 [M+H]$^+$.

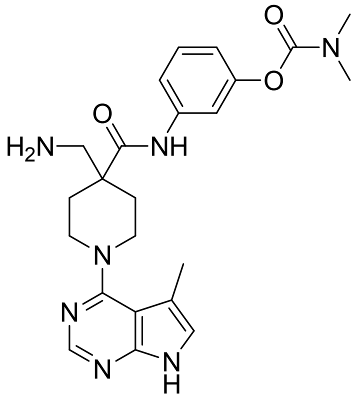

(**7**) LX7101

Step 1 – Synthesis of O-1-tert-butyl O-4-methyl 4-(benzyloxycarbonylaminomethyl)piperidine-1,4-dicarboxylate

A solution of methyl 4-aminomethyl-1-boc-piperidine-4-carboxylate (1.00 g, 3.67 mmol) and *N*-ethyldiisopropylamine (0.75 mL, 4.41 mmol) in CH_2Cl_2 (20 mL) was cooled in an ice-bath before the addition of benzyl chloroformate (0.55 mL, 3.86 mmol). Upon completion of addition, the reaction mixture was stirred at rt overnight. A saturated aqueous solution of $NaHCO_3$ (25 mL) was added and the mixture stirred vigorously for 20 min before being passed through a phase separator. The filtrate

was concentrated under reduced pressure and the residue purified by automated flash column chromatography using an ISCO system (24 g silica; gradient from 20-50% EtOAc in petroleum ether) to give *O*-1-tert-butyl *O*-4-methyl 4-(benzyloxycarbonylaminomethyl)piperidine-1,4-dicarboxylate as a yellow oil (1.350 g, 3.155 mmol, 86% yield). ^{1}H NMR (500 MHz, DMSO-d_6) δ 7.44 (t, *J*= 6.6 Hz, 1H), 7.40 – 7.27 (m, 5H), 5.00 (s, 2H), 3.71 (app d, *J*= 13.3 Hz, 2H), 3.57 (s, 3H), 3.17 (d, *J*= 6.5 Hz, 2H), 2.88 – 2.67 (br s, 2H), 1.89 (app d, *J*= 13.4 Hz, 2H), 1.38 (s, 9H), 1.30 (ddd, *J*= 13.8, 11.1, 4.2 Hz, 2H). ACQUITY UPLC® HSS T3 1.8µm: Rt = 1.90 min; m/z 307.1 [M-Boc+H]$^+$.

Step 2 – Synthesis of tert-butyl 4-(benzyloxycarbonylaminomethyl)-4-[[3-(dimethylcarbamoyloxy)phenyl]carbamoyl]piperidine-1-carboxylate

To a solution of *O*-1-tert-butyl *O*-4-methyl 4-(benzyloxycarbonylaminomethyl)piperidine-1,4-dicarboxylate (600 mg, 1.4 mmol) in MeOH (10 mL) was added an aqueous solution of 1M NaOH (1.68 mL, 1.68 mmol). The solution was heated to 50 ˚C overnight. Once cooled, the reaction mixture was acidified to pH 4 with 1M HCl and concentrated under reduced pressure. CH_2Cl_2 (15 mL) was added, the layers seperated and the aqueous phase back-extracted with CH_2Cl_2 (2 × 15 mL). The combined organic phase was washed with brine (15 mL), dried over $MgSO_4$, filtered and concentrated under reduced pressure to give the desired acid product as a yellow oil (690 mg), which was used in the next step without further purification. To a solution of the crude acid in CH_2Cl_2 (20 mL) was added pyridine (0.40 mL) and DMF (0.050 mL). The mixture was degassed with nitrogen for 10 min, cooled using an ice-bath and oxalyl chloride (0.14 mL, 1.68 mmol) was added drop-wise to the solution. Following the evolution of gas, the cooling bath was removed and the mixture stirred at rt for 30 min. The solution of acid chloride was cooled in an ice-bath and 3-aminophenyl dimethylcarbamate (250 mg, 1.39 mmol) was added in a single portion before stirring at rt overnight. LCMS analysis indicated incomplete conversion the product, therefore *N*-(3-dimethylaminopropyl)-*N*′-ethylcarbodiimide hydrochloride (150 mg, 0.780 mmol) was added and stirring continued overnight. The reaction mixture was diluted with CH_2Cl_2 (20 mL) and washed sequentially with 1 M HCl (3 × 20 mL) and brine (1 × 20 mL). The organic phase was dried over $MgSO_4$, filtered and concentrated under reduced pressure to give the crude product, which was purified by automatic flash column chromatography using an ISCO system (24 g silica; gradient of 30-70% EtOAc in petroleum ether) to give *tert*-butyl 4-(benzyloxycarbonylaminomethyl)-4-[[3-(dimethylcarbamoyloxy)phenyl]carbamoyl]piperidine-1-carboxylate as a light yellow oil (440 mg, 0.793 mmol, 57% yield).^{1}H NMR (400 MHz, Chloroform-*d*) δ 8.02 (s, 1H), 7.49 (s, 1H), 7.29 – 7.18 (m, 5H), 7.08 (dd, *J* = 8.2, 7.3 Hz, 1H), 6.92 (dd, *J* = 8.3 Hz, 1H), 6.74 (d, *J* = 8.1 Hz, 1H), 5.56 (t, *J* = 6.3 Hz, 1H), 5.01 (s, 2H), 3.55 – 3.48 (m, 2H), 3.40 – 3.35 (m, 1H), 3.30 – 3.20 (m, 2H), 3.02 (s, 3H), 2.93 (s, 3H), 1.97 – 1.88 (m, 2H), 1.52 – 1.43 (m, 2H), 1.39 (s, 9H). ACQUITY UPLC® BEH 1.8 µm: Rt = 1.89 min; *m/z* 553.2 [M-H]$^-$.

Step 3 – Synthesis of [3-[[4-(benzyloxycarbonylaminomethyl)-1-(5-methyl-7H-pyrrolo[2,3-d]pyrimidin-4-yl)piperidine-4-carbonyl]amino]phenyl] N,N-dimethylcarbamate

To a suspension of *tert*-butyl 4-(benzyloxycarbonylaminomethyl)-4-[[3-(dimethylcarbamoyloxy)phenyl]carbamoyl]piperidine-1-carboxylate (220 mg, 0.400 mmol) in MeOH (2 mL) was added 4M Hydrochloric acid in dioxane (0.30 mL, 1.19 mmol). The reaction mixture was heated to 50 °C for 1 h. Heating was ceased as TLC analysis indicated complete deprotection. The reaction mixture was concentrated under reduced pressure in a fumehood using MeOH to form an azeotrope to give the amine hydrochloride as a white solid (180 mg). To the amine salt dissolved in isopropanol (4 mL) was added *N*-ethyldiisopropylamine (0.20 mL, 1.19 mmol) and 4-chloro-5-methyl-7*H*-pyrrolo[2,3-*d*]pyrimidine (66.5 mg, 0.400 mmol). The mixture was heated in a sealed vial at 120°C overnight. LCMS analysis indicated a small amount of amine starting material remained, therefore additional 20 mg of the 4-chloro-5-methyl-7*H*-pyrrole[2,3-*d*]pyrimidine (20 mg, 0.12 mmol) was added

and reaction continued at 120°C for a further 6 h. LCMS analysis indicated little change in reaction profile. The reaction mixture was concentrated under reduced pressure and the residue re-dissolved in CH_2Cl_2 (20 mL). The organic phase was washed with water (10 mL), brine (10 mL), dried over $MgSO_4$, filtered and concentrated under reduced pressure to give the crude product, which was purified by automated by flash column chromatography using an ISCO system (12 g silica; gradient of 0-4% MeOH in EtOAc) to give [3-[[4-(benzyloxycarbonylaminomethyl)-1-(5-methyl-7*H*-pyrrolo[2,3-*d*]pyrimidin-4-yl)piperidine-4-carbonyl]amino]phenyl] *N*,*N*-dimethylcarbamate (195 mg, 0.316 mmol, 80% yield). ^{1}H NMR (500 MHz, Chloroform-*d*) δ 9.38 (s, 1H), 8.34 (s, 1H), 7.89 (s, 1H), 7.53 (s, 1H), 7.34 – 7.27 (m, 5H), 7.25 – 7.20 (m, 1H), 7.19 – 7.13 (m, 1H), 6.90 – 6.84 (m, 2H), 5.44 – 5.40 (m, 1H), 5.09 (s, 2H), 3.80 – 3.72 (m, 2H), 3.63 – 3.47 (m, 4H), 3.08 (s, 3H), 2.99 (s, 3H), 2.38 (s, 3H), 2.26 – 2.17 (m, 2H), 1.87 – 1.78 (m, 2H). ACQUITY UPLC® BEH 1.8 µm: Rt = 1.51 min; *m/z* 586.1 $[M+H]^+$.

Step 4 – Synthesis of LX-7101

A solution of [3-[[4-(benzyloxycarbonylaminomethyl)-1-(5-methyl-7*H*-pyrrolo[2,3-*d*]pyrimidin-4-yl)piperidine-4-carbonyl]amino]phenyl] *N*,*N*-dimethylcarbamate (195 mg, 0.320 mmol) in EtOH (150 mL) was passed through a 10% Pd/C cartridge at 1 mL/min under 5 bar of hydrogen at 30°C using a ThalesNano H-cube mini system. The reaction mixture was continuously passed through the system and monitored by LCMS analysis. Following 6 h of cycling, the reaction mixture was concentrated under reduced pressure and purified by reverse-phase automatic column chromatography using an ISO AQQCPrep C_{18} (10-100% gradient of water-MeOH) to give LX-7101 (**7**, 30 mg, 20%) as a colourless solid. ^{1}H NMR (500 MHz, DMSO-d_6) δ 11.49 (s, 1H), 8.18 (s, 1H), 7.50 (t, *J* = 2.2 Hz, 1H), 7.37 (d, *J* = 8.2, 1.5 Hz, 1H), 7.28 (app t, *J* = 8.1 Hz, 1H), 7.04 (s, 1H), 6.80 – 6.77 (m, 1H), 3.68 (d, *J* = 13.4 Hz, 2H), 3.32 – 3.29 (m, 2H), 3.03 (s, 3H), 2.90 (s, 3H), 2.84 (s, 2H), 2.37 – 2.32 (m, 3H), 2.23 – 2.12 (m, 2H), 1.67 – 1.55 (m, 2H). ACQUITY UPLC® BEH 1.8 µm: Rt = 1.22 min; m/z 452.2 $[M+H]^+$.

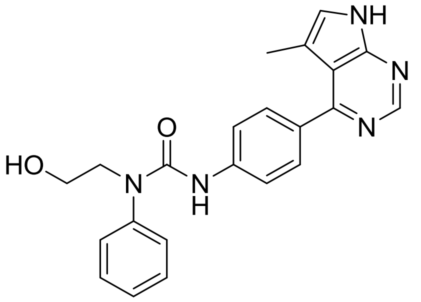

(**8**) SR7826. Supplier = Tocris.

^{1}H NMR (500 MHz, DMSO-d_6) δ 11.87 (s, 1H), 8.70 (s, 1H), 8.32 (s, 1H), 7.58 – 7.53 (m, 4H), 7.48 – 7.42 (m, 2H), 7.41 – 7.36 (m, 2H), 7.34 (s, 1H), 7.33 – 7.27 (m, 1H), 5.01 (s, 1H), 3.77 (t, *J* = 6.2 Hz, 2H), 3.57 (t, *J* = 6.2 Hz, 2H), 2.06 (s, 3H). ACQUITY UPLC® BEH C18 1.7 µm: Rt = 1.33 min; *m/z* 388.1 $[M+H]^+$.

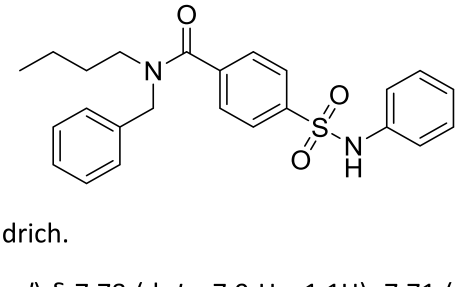

(**9**) TH-257. Supplier = Sigma Aldrich.

^{1}H NMR (500 MHz, Chloroform-*d*) δ 7.78 (d, *J* = 7.9 Hz, 1.1H), 7.71 (d, *J* = 8.0 Hz, 0.9H), 7.44 (dd, *J* = 12.1, 7.9 Hz, 2H), 7.39 – 7.17 (m, 5H), 7.17 – 7.10 (m, 1.1H), 7.10 – 7.00 (m, 3H), 6.67 (s, 0.49H), 6.64

(s, 0.42H), 4.74 (s, 1.1H), 4.37 (s, 0.9H), 3.47 (t, *J* = 7.7 Hz, 0.9H), 3.01 (t, *J* = 7.7 Hz, 1.1H), 1.62 (app pent, *J* = 7.7 Hz, 0.9H), 1.41 (app pent, *J* = 7.9 Hz, 1.1H), 1.35 (app sext, *J* = 7.6 Hz, 0.9H), 1.04 (app sext, *J* = 7.6 Hz, 1.1H), 0.93 (t, *J* = 7.4 Hz, 1.4H), 0.71 (t, *J* = 7.4 Hz, 1.6H). Mixture of rotamers in 1:0.85 ratio. ACQUITY UPLC® BEH C18 1.7 µm: Rt = 1.93 min; *m/z* 423.2[M+H]$^+$.

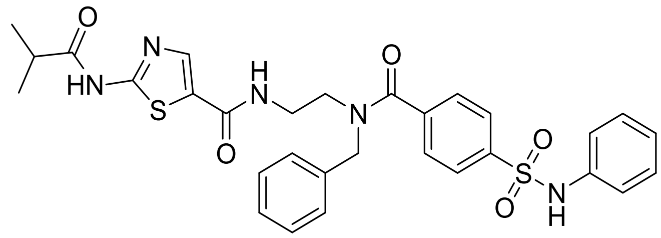

(**10**) TH-460

Synthesis described in main text.

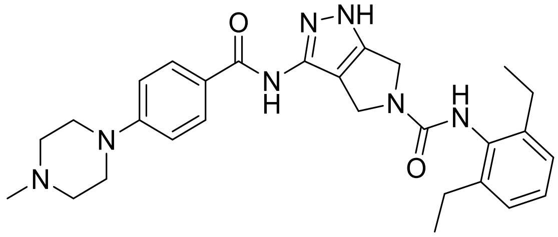

(**11**) PHA-680632. Supplier = Cambridge Biosciences.

^{1}H NMR (500 MHz, Chloroform-*d*) δ 7.97 – 7.81 (m, 2H), 7.76 – 7.61 (m, 1H), 7.15 (dd, *J* = 8.3, 6.7 Hz, 1H), 7.08 (d, *J* = 7.5 Hz, 2H), 7.05 – 6.91 (m, 2H), 5.80 – 5.68 (m, 4H), 4.71 – 4.36 (m, 4H), 2.57 (q, *J* = 7.6 Hz, 4H), 2.43 (dd, *J* = 5.1 Hz, 4H), 2.22 (s, 3H), 1.13 (t, *J* = 7.6 Hz, 6H). ACQUITY UPLC® BEH C_{18} 1.7 µm: R_t = 1.41 min; *m/z* 502.5 [M+H]$^+$.

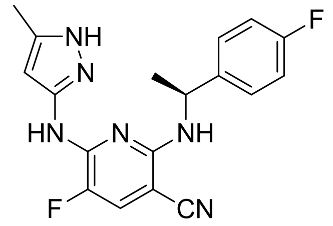

(**12**) AZ-960. Supplier = Axon MedChem.

^{1}H NMR (500 MHz, DMSO-d_6) δ 11.98 (s, 1H), 9.34 (s, 1H), 7.59 (d, *J* = 10.8 Hz, 1H), 7.43 – 7.30 (m, 2H), 7.15 – 7.04 (m, 2H), 7.00 (d, *J* = 7.5 Hz, 1H), 5.94 (s, 1H), 5.11 (p, *J* = 7.1 Hz, 1H), 2.20 (s, 3H), 1.47 (d, *J* = 7.1 Hz, 3H). ACQUITY UPLC® BEH C_{18} 1.7 µm: R_t = 1.69 min; *m/z* 355.1 [M+H]$^+$.

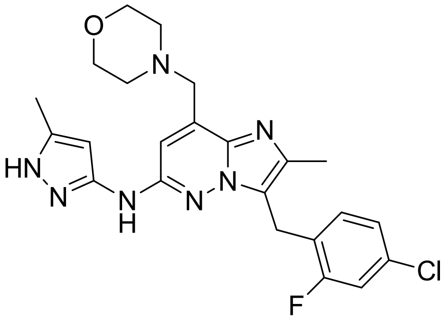

(**13**) Gandotinib. Supplier = Axon MedChem.

^{1}H NMR (500 MHz, DMSO-d_6) δ 11.82 (s, 1H), 9.40 (s, 1H), 7.43 (dd, J = 10.0, 2.1 Hz, 1H), 7.18 – 7.06 (m, 1H), 7.04 – 6.92 (m, 2H), 6.07 (s, 1H), 4.22 (s, 2H), 3.75 (d, J = 1.3 Hz, 2H), 3.64 (app t, J = 4.6 Hz, 4H), 3.30 – 3.28 (m, 4H), 2.31 (s, 3H), 2.18 (s, 3H). ACQUITY UPLC® BEH C_{18} 1.7 µm: R_t = 1.81 min; *m/z* 470.2 [M+H]$^+$.

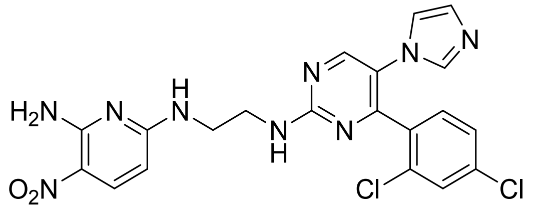

(**14**) CHIR-98014. Supplier = Key Organics.

^{1}H NMR (500 MHz, DMSO-d_6) δ 8.50 (d, J = 15.5 Hz, 1H), 8.20 – 8.00 (m, 2H), 7.99 – 7.82 (m, 2H), 7.62 (s, 1H), 7.52 (s, 1H), 7.45 (s, 2H), 6.99 (s, 1H), 6.87 (t, J = 1.2 Hz, 1H), 5.95 (s, 1H), 3.67 – 3.42 (m, 4H). ACQUITY UPLC® BEH C_{18} 1.7 µm: R_t = 1.48 min; *m/z* 486.1 [M+H]$^+$.

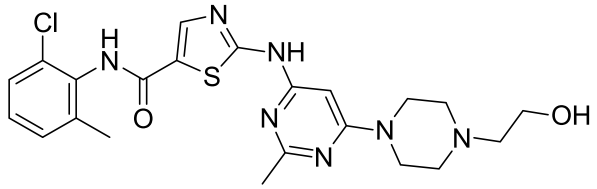

(**15**) Dasatinib. Supplier = Apollo Scientific.

^{1}H NMR (500 MHz, DMSO-d_6) δ 11.45 (s, 1H), 9.87 (s, 1H), 8.21 (s, 1H), 7.43 – 7.34 (m, 1H), 7.34 – 7.20 (m, 2H), 6.05 (s, 1H), 4.44 (t, J = 5.3 Hz, 1H), 3.57 – 3.46 (m, 6H), 2.49 – 2.46 (m, 4H), 2.42 (t, J = 6.3 Hz, 2H), 2.40 (s, 3H), 2.24 (s, 3H). ACQUITY UPLC® BEH C18 1.7 µm: Rt = 1.45 min; *m/z* 488.2 [M+H]$^+$.

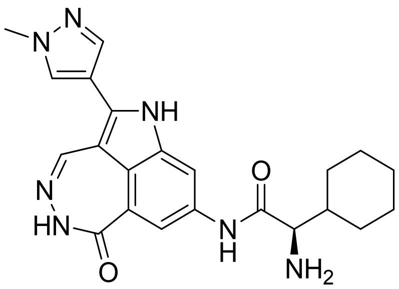

(**16**) PF-477736. Supplier = Cambridge Bioscience

^{1}H NMR (500 MHz, DMSO-d_6): 11.91 (s, 1H), 10.23 (s, 1H), 8.30 (s, 1H), 8.10 (d, J = 1.8 Hz, 1H), 7.91 (d, J = 0.8 Hz, 1H), 7.58 (s, 1H), 7.57 (d, J = 1.8 Hz, 1H), 3.92 (s, 3H), 3.09 (d, J = 5.8 Hz, 1H), 1.79 – 1.66 (m, 4H), 1.64 – 1.50 (m, 3H), 1.27 – 0.90 (m, 7H). ACQUITY UPLC® BEH C18 1.7 µm: Rt = 1.29 min; 420.3 [M+H]$^+$.

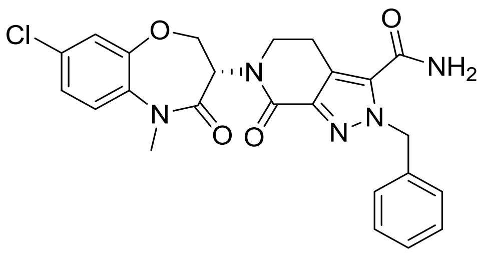

(**17**) LIJTF500025

Step 1 – Synthesis of dimethyl 1-benzyl-4-[(E)-2-ethoxyvinyl]pyrazole-3,5-dicarboxylate

A suspension of dimethyl 4-iodo-1H-pyrazole-3,5-dicarboxylate (5.0 g, 16.1 mmol) and potassium carbonate (3.0 g, 21.4 mmol) in DMF (30 mL) was cooled in an ice-bath. benzyl bromide (1.95 mL, 16.4 mmol) was added slowly over 5 minutes. The bath was removed and the mixture stirred at room temperature for 1 hour. Poured onto 350 mL ice-cold water and the resultant precipitate cold-filtered and washed with 100 mL cold water to give dimethyl 1-benzyl-4-iodo-pyrazole-3,5-dicarboxylate (6.1 g, 90% yield) as a colourless solid. ^{1}H NMR (500 MHz, Chloroform-*d*) δ 7.37 – 7.27 (m, 3H), 7.25 – 7.18 (m, 2H), 5.84 (s, 2H), 3.99 (s, 3H), 3.89 (s, 3H). ACQUITY UPLC® BEH 1.8µm: Rt = 1.8 min; m/z 400.9 $[M+H]^+$.

Step 2 – Synthesis of dimethyl 1-benzyl-4-[(E)-2-ethoxyvinyl]pyrazole-3,5-dicarboxylate

A mixture of dimethyl 1-benzyl-4-iodo-pyrazole-3,5-dicarboxylate (2.0 g, 4.8 mmol), (E)-(2-Ethoxyvinyl)boronic acid, pinacol ester (1.7 mL, 8.1 mmol), cesium carbonate (3.3 g, 10.1 mmol) and [1,1'-bis(diphenylphosphino)ferrocene]dichloropalladium(II) (0.3 g, 0.5 mmol) in monoglyme (20 mL) and Water (2 mL) in a microwave vial was degassed with nitrogen for 10 minutes before sealing and heating conventionally to 90 ^{o}C overnight. After 18 hours, mixture was diluted with 60 mL EtOAc and 20 mL water, filtered, separated and aqueous extracted with further 20 mL EtOAc. Combined organic dried over sodium sulphate, filtered and absorbed onto Celite. Purification by flash column chromatography using an ISCO system (40 g silica; 0-30% EtOAc in PE gradient) afforded dimethyl 1-benzyl-4-[(E)-2-ethoxyvinyl]pyrazole-3,5-dicarboxylate (1.4 g, 77% yield) as a brown oil. ^{1}H NMR (400 MHz, Chloroform-*d*) δ 7.40 (d, *J* = 12.8 Hz, 1H), 7.32 – 7.25 (m, 3H), 7.17 (d, *J* = 7.1 Hz, 2H), 6.19 (d, *J* = 12.9 Hz, 1H), 5.77 (s, 2H), 3.99 – 3.92 (m, 5H), 3.83 (s, 3H), 1.35 (t, *J* = 7.1 Hz, 3H). ACQUITY UPLC® BEH 1.8µm: Rt = 1.9 min; m/z 367.1 $[M+Na]^+$.

Step 3 – Synthesis of 3(S)-3-amino-8-chloro-5-methyl-2,3-dihydro-1,5-benzoxazepin-4-one hydrochloride

To an ice-cold solution of 4-chloro-2-fluoroaniline (1.68 mL, 14.3 mmol) and Boc-Ser(*t*Bu)-OH (3.79 g, 14.5 mmol) in CH_2Cl_2 (115 mL) was added drop-wise *N*-ethyldiisopropylamine (5.07 mL, 29.6 mmol) followed by a solution of propylphosphonic anhydride (6.49 mL, 21.8 mmol, 50% sol. In EtOAc). Following completion of addition, the mixture was stirred for 15 min before removal of the cooling bath. The mixture was left to stir at room temperature for 48 h. UPLC analysis showed two species, the most prominent of which corresponded to the product. The reaction mixture was washed with aqueous 1M HCl (50 mL × 2), saturated aqueous $NaHCO_3$ (50 mL × 2), water (50 mL) and brine (50 mL). After drying over $MgSO_4$ and filtration, the organic solution was concentrated under reduced pressure to afford the crude product, which was purified by automated column chromatography on silica (40 g, ISCO, very slow gradient from petroleum ether to EtOAc over 30CV) to give tert-butyl *N*-[(1S)-1-(*tert*-butoxymethyl)-2-(4-chloro-2-fluoro-anilino)-2-oxo-ethyl]carbamate as a colourless solid (4.02, 71% yield). ^{1}H NMR (500 MHz, Chloroform-*d*) δ 9.09 (s, 1H), 8.33 (t, *J* = 8.5 Hz, 1H), 7.16 – 7.12 (m, 2H),

5.54 (s, 1H), 4.40 – 4.26 (m, 1H), 3.94 – 3.83 (m, 1H), 3.48 – 3.38 (m, 1H), 1.49 (s, 9H), 1.27 (s, 9H). ACQUITY UPLC® BEH C_{18} 1.7 µm: Rt = 1.95 min; m/z 387.1[M-H]$^-$ for ^{35}Cl.

To a solution of *tert*-butyl *N*-[(1*S*)-1-(*tert*-butoxymethyl)-2-(4-chloro-2-fluoro-anilino)-2-oxo-ethyl]carbamate (1.99 g, 4.85 mmol) in DMF (35 mL) was added cesium carbonate (1.59 g, 4.85 mmol). The suspension was cooled in an ice bath and iodomethane (0.30 mL, 4.85 mmol) was added slowly. The cooling bath was removed after 1 h and the mixture left to stir at room temperature overnight. The reaction mixture was poured in water (80 mL) and extracted with CH_2Cl_2 (3 × 20 mL) and the combined organic extracts washed with water (4 × 20 mL), dried over $MgSO_4$, filtered and concentrated under reduced pressure to give the crude product, which was purified by automated column chromatography on silica (24 g, ISCO, slow gradient from petroleum ether to EtOAc containing 1% triethylamine) and a second time (12 g, ISCO, very slow gradient from petroleum ether to CH_2Cl_2 over 25CV) to give *tert*-butyl *N*-[(1*S*)-1-(tert-butoxymethyl)-2-(4-chloro-2-fluoro-N-methyl-anilino)-2-oxo-ethyl]carbamate. ^{1}H NMR (500 MHz, Chloroform-*d*) rotamers present in spectrum δ 7.38 (*app* t, *J* = 8.3 Hz, 0.6H), 7.32 (*app* t, *J* = 8.3 Hz, 0.4H), 7.22 – 7.10 (m, 2H), 5.22 (d, *J* = 8.5 Hz, 0.6H), 5.11 (d, *J* = 9.2 Hz, 0.6H), 4.37 (dt, *J* = 8.4, 5.7 Hz, 0.6H), 4.28 (dt, *J* = 14.1, 6.9 Hz, 0.4H), 3.43 – 3.35 (m, 0.9H), 3.35 – 3.27 (m, 1.1H), 3.26 – 3.16 (m, 4.1H), 1.38 (s, 3.6H), 1.35 (s, 3.2H), 1.09 (s, 4.7H), 1.03 (s, 5.7H). ACQUITY UPLC® BEH C_{18} 1.7 µm: Rt = 1.89 min; m/z 425.1 [M+H + Na]$^+$.

To a solution of *tert*-butyl *N*-[(1*S*)-1-(tert-butoxymethyl)-2-(4-chloro-2-fluoro-*N*-methyl-anilino)-2-oxo-ethyl]carbamate (0.31 g, 0.77 mmol) in CH_2Cl_2 (4 mL) was added 4M HCl in dioxane (1.06 mL, 4.24 mmol). The reaction mixture was allowed to stir at room temperature for 48 h. UPLC analysis indicated full removal of both the Boc and *tert*-butyl ether protecting groups. The mixture was concentrated under reduced pressure in a fumehood to give the crude product, which was triturated with diethyl ether (10 mL) to give (2*S*)-2-amino-*N*-(4-chloro-2-fluoro-phenyl)-3-hydroxy-*N*-methyl-propanamide hydrochloride (210 mg, 0.705 mmol, 96% yield) as a colourless solid. ^{1}H NMR (500 MHz, DMSO-*d*6) spectrum too complex to assign due to mixture of HCl salt/free base and rotamers from hindered rotation. ACQUITY UPLC® BEH C_{18} 1.7 µm: Rt = 0.49 min; m/z 247.0 [M-HCl+H]$^+$.

To an ice-cold solution of (2*S*)-2-amino-*N*-(4-chloro-2-fluoro-phenyl)-3-hydroxy-*N*-methyl-propanamide hydrochloride (210 mg, 0.74 mmol) in chloroform (10 mL) was added triethylamine (0.26 mL, 1.86 mmol) followed by trityl chloride (0.25 g, 0.89 mmol). The cooling bath was removed after 30 min, and the mixture allowed to stir at room temperature overnight. The reaction mixture was diluted with CH_2Cl_2 (40 mL) and washed with a saturated aqueous solution of sodium hydrogen carbonate (40 mL), before being dried over $MgSO_4$ and filtered. The filtrate was concentrated under reduced pressure to give the crude product, which was purified by trituration - 5% EtOAc in petroleum ether (50 mL) was added and the mixture stirred vigorously for 10 min. The majority of the material dissolved and the supernatant transferred into a separate flask, however, upon light scratching with a glass pipette a colourless solid precipitated out from this supernatant. The solid was collected with filter paper and dried, to give (2*S*)-*N*-(4-chloro-2-fluoro-phenyl)-3-hydroxy-*N*-methyl-2-(tritylamino)propanamide (976 mg, 1.780 mmol, 57% yield) as a colourless solid. ^{1}H NMR (500 MHz, Chloroform-*d*) spectrum too complex to assign due to rotamers from hindered rotation. ACQUITY UPLC® BEH C_{18} 1.7 µm: Rt = 1.94 min; m/z 511.2 [M+H+Na]$^+$.

Cesium carbonate (2.3 g, 7.0 mmol) was added to a solution of (2S)-N-(4-chloro-2-fluoro-phenyl)-3-hydroxy-N-methyl-2-(tritylamino)propanamide (1.0 g, 2.0 mmol) in DMF (5 mL). The mixture was heated to 50° C overnight. Once cooled, the reaction mixture was diluted with EtOAc (50 mL) and washed with water (5 x 30 mL). The organic phase was dried over magnesium sulfate, filtered and concentrated under reduced pressure to give the crude product, which was purified by automated column chromatography on silica (eluting with petroleum ether to 20% EtOAc in petroleum ether) to

give (3S)-8-chloro-5-methyl-3-(tritylamino)-2,3-dihydro-1,5-benzoxazepin-4-one (0.6 g, 61% yield) as a colourless solid. ^{1}H NMR (500 MHz, Chloroform-*d*) δ 7.42 – 7.36 (m, 6H), 7.24 – 7.19 (m, 6H), 7.19 – 7.14 (m, 3H), 7.11 – 7.06 (m, 1H), 7.04 (d, *J* = 2.4 Hz, 1H), 6.80 (d, *J* = 8.5 Hz, 1H), 4.53 (dd, *J* = 9.9, 7.2 Hz, 1H), 4.39 (dd, *J* = 11.8, 9.9 Hz, 1H), 3.56 – 3.46 (m, 1H), 3.35 – 3.27 (m, 1H), 2.86 (s, 3H). ACQUITY UPLC® BEH C18 1.7 µm: Rt = 2.08 min; *m/z* 491.1 $[M+Na]^+$.

To a solution of (3S)-8-chloro-5-methyl-3-(tritylamino)-2,3-dihydro-1,5-benzoxazepin-4-one (571.0 mg, 1.2 mmol) in 1,4-dioxane (24 mL) and methanol (4 mL) was added 4 M HCl in dioxane (1.4 mL, 5.4 mmol). The mixture was left to stir overnight at room temperature. An additional portion of 4 M HCl in dioxane (1.4 mL, 5.4 mmol) was added. After two hours, the mixture was concentrated under reduced pressure in a fumehood and diethyl ether (50 mL) was added. The solid was isolated by filtration and dried under reduced pressure to give (3S)-3-amino-8-chloro-5-methyl-2,3-dihydro-1,5-benzoxazepin-4-one hydrochloride (270 mg , 80% yield) as a colourless solid. ^{1}H NMR (500 MHz, Methanol-d_4) δ 7.45 (d, *J* = 8.6 Hz, 1H), 7.37 (dd, *J* = 8.6, 2.4 Hz, 1H), 7.35 – 7.32 (m, 1H), 4.65 (dd, *J* = 9.9, 7.6 Hz, 1H), 4.50 (dd, *J* = 11.3, 9.9 Hz, 1H), 4.37 (dd, *J* = 11.2, 7.6 Hz, 1H), 3.42 (s, 3H). ACQUITY UPLC® BEH C18 1.7 µm: Rt = 0.72 min; m/z 227.0 $[M+H-HCl]^+$ for ^{35}Cl.

Step 4 – Synthesis of dimethyl 1-benzyl-4-[2-[[(3S)-8-chloro-5-methyl-4-oxo-2,3-dihydro-1,5-benzoxazepin-3-yl]amino]ethyl]pyrazole-3,5-dicarboxylate

An ice-cold solution of dimethyl 1-benzyl-4-[(E)-2-ethoxyvinyl]pyrazole-3,5-dicarboxylate (400 mg, 1.2 mmol) in THF (4 mL) was added slowly to an ice-cold solution of 6 M hydrochloric acid (4.0 mL, 24.0 mmol) and resultant solution stirred at room temperature. After 1 hour, poured onto 50 mL sat. NaHCO3 (pH was 7). Extracted 3 x 25 mL EtOAc. Combined EtOAc washed with 25 mL sat. NaHCO3 and 25 mL brine, dried over MgSO4, filtered and concentrated to give dimethyl 1-benzyl-4-(2-oxoethyl)pyrazole-3,5-dicarboxylate (400 mg,quantitative yield) as a yellow oil which was taken forward to next step without further purification or characterisation. A suspension this aldehyde (150 mg, 0.47 mmol) and (3S)-3-amino-8-chloro-5-methyl-2,3-dihydro-1,5-benzoxazepin-4-one hydrochloride (135 mg, 0.51 mmol) in methanol (1.5 mL) had acetic acid (0.1 mL) added and the mixture stirred for 15 minutes at RT until a solution resulted. Borane-2-methylpyridine complex (65 mg, 0.61 mmol) was added and the resultant solution stirred at RT for 1 hours. Poured onto 30 mL at. NaHCO3 (pH was 8). Extraced 3 x 25 mL EtOAc. Combined organic layer dried over MgSO4, filtered and concentrated to a yellow oil.Purification by flash column chromatography using an ISCO system (24 g silica; 30-100% EtOAc in PE gradient) gave dimethyl 1-benzyl-4-[2-[[(3S)-8-chloro-5-methyl-4-oxo-2,3-dihydro-1,5-benzoxazepin-3-yl]amino]ethyl]pyrazole-3,5-dicarboxylate (80 mg, 27% yield) as a colourless glass. Taken forward to next step without further purification or characterisation.

Step 5 – Synthesis of 2-benzyl-6-[(3S)-8-chloro-5-methyl-4-oxo-2,3-dihydro-1,5-benzoxazepin-3-yl]-7-oxo-4,5-dihydropyrazolo[3,4-c]pyridine-3-carboxamide

A solution of dimethyl 1-benzyl-4-[2-[[(3S)-8-chloro-5-methyl-4-oxo-2,3-dihydro-1,5-benzoxazepin-3-yl]amino]ethyl]pyrazole-3,5-dicarboxylate (80.0 mg, 0.15 mmol) and lithium hydroxide monohydrate (36.0 mg, 0.86 mmol) in THF (1 mL)/Water (1 mL)/methanol (1 mL) was stirred at room temperature for 20 hours. Volatile solvents removed on Biotage V10 and pH adjusted to 1 with 1 M HCl. Resultant white solid isolated by filtration and washed with 10 mL water then dried at pump for an hour. This material was suspended with N-(3-dimethylaminopropyl)-N′-ethylcarbodiimide hydrochloride (40.0 mg, 0.21 mmol) and 1-hydroxybenzotriazole hydrate (25.0 mg, 0.16 mmol) in DCM (5 mL). N-Ethyldiisopropylamine (62 uL, 0.36 mmol) was added and resultant solution left to stand at room temperature for 24 hours. Transferred to microwave vial. N-(3-dimethylaminopropyl)-N′-ethylcarbodiimide hydrochloride (70.0 mg, 0.37 mmol), 1-hydroxybenzotriazole hydrate (45.0 mg,

0.29 mmol) and ammonium chloride (34.0 mg, 0.64 mmol) were added, followed by N-Ethyldiisopropylamine (62 uL, 0.36 mmol). Vial sealed and heated to 45 °C for 24 hours. Partitioned with 3 mL 0.6 M citric acid and passed through phase separator. Partitioned with 3 mL saturated NaHCO3 and passed through phase separator. Solvent removed and residue purified by flash column chromatography using an ISCO system (24 g silica; 50-90% EtOAc in PE gradient). Dried in vacuum oven for 24 hours to give 2-benzyl-6-[(3S)-8-chloro-5-methyl-4-oxo-2,3-dihydro-1,5-benzoxazepin-3-yl]-7-oxo-4,5-dihydropyrazolo[3,4-c]pyridine-3-carboxamide (**17**, 9 mg, 12% yield) as a colourless solid. ^{1}H NMR (500 MHz, DMSO-d_6) δ 7.83 (s, 1H), 7.73 (s, 1H), 7.54 (d, *J* = 8.6 Hz, 1H), 7.40 (dd, *J* = 8.6, 2.5 Hz, 1H), 7.38 (d, *J* = 2.4 Hz, 1H), 7.34 – 7.28 (m, 2H), 7.28 – 7.23 (m, 1H), 7.16 (dd, *J* = 7.2, 1.6 Hz, 2H), 5.63 (s, 2H), 5.54 (dd, *J* = 12.0, 7.8 Hz, 1H), 4.89 (dd, *J* = 12.0, 10.1 Hz, 1H), 4.39 (dd, *J* = 10.0, 7.8 Hz, 1H), 3.98 (ddd, *J* = 12.2, 7.0, 5.1 Hz, 1H), 3.59 (ddd, *J* = 12.8, 8.8, 4.6 Hz, 1H), 3.28 (s, 3H), 3.04 (ddd, *J* = 15.8, 8.8, 5.0 Hz, 1H), 2.84 (ddd, *J* = 16.0, 7.0, 4.7 Hz, 1H). ACQUITY UPLC® BEH 1.7µm: Rt = 1.7 min; m/z 480.1 $[M+H]^+$.

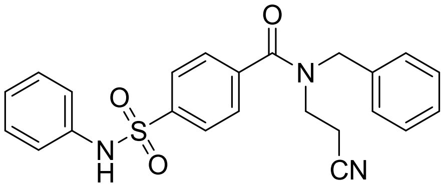

(**21**)

Step 1 Synthesis of 4-(phenylsulfamoyl)benzoic acid

To a stirring solution of 4-(chlorosulfonyl)benzoic acid (10.20 g, 46.2 mmol) in THF (150 mL) was added aniline (20.4 mL, 223.9 mmol) quickly drop-wise. Upon completion of addition, the mixture was allowed to stir at rt for 4 h. LCMS analysis showed reaction completion with the major product being the desired acid, although the presence of the product from bis-addition of the aniline was also observed. The reaction mixture was poured onto ice-cold water (800 mL), filtered using a Büchner funnel under reduced pressure, washed with additional water (200 mL) and finally, dried in a vacuum oven set at 40 °C over the weekend to give 4-(phenylsulfamoyl)benzoic acid (10.58 g, 36.2 mmol, 78% yield) as a beige solid. ^{1}H NMR (500 MHz, DMSO-d_6) δ 13.38 (s, 1H), 10.42 (s, 1H), 8.10 – 7.99 (m, 2H), 7.87 – 7.80 (m, 2H), 7.26 – 7.19 (m, 2H), 7.10 – 7.06 (m, 2H), 7.04 (ddt,*J*= 8.5, 7.1, 1.2 Hz, 1H). ACQUITY UPLC® BEH C18 1.7 µm: Rt = 1.49 min; m/z 277.0 $[M]^+$.

Step 2 Synthesis of N-benzyl-N-(2-cyanoethyl)-4-(N-phenylsulfamoyl)benzamide

4-(Phenylsulfamoyl)benzoic acid (99.5 mg, 0.36 mmol), EDC·HCl (82.5 mg, 0.43 mmol) and HOBt (64.3 mg, 0.41 mmol) were suspended in dichloromethane (8 mL) and the mixture degassed with nitrogen for 10 min, after which the reaction mixture was stirred at rt for 1 h. 3-(Benzylamino)propionitrile (0.85 µL, 0.54 mmol) was added *via* pipette and the mixture stirred overnight at rt. The reaction mixture was adsorbed onto silica, concentrated under reduced pressure and purified by automatic flash column chromatography (silica, 12 g, petrol:EtOAc; 100:0 to 0:100 over 25 CV), followed by a second round of column chromatography (silica, 12 g, CH_2Cl_2:MeOH; 100:0 to 99:1 over 30 CV) to give near-pure product. Finally, the impure desired product was dissolved in MeCN (2.0 mL) and purified by reverse-phase HPLC preparative chromatography (C_{18}, 20 × 150 mm, H_2O:MeCN; 90:10 to 80:20 over 25 min). The residue following concentration under reduced pressure was further dried in a vacuum oven at 40°C to afford *N*-benzyl-*N*-(2-cyanoethyl)-4-(*N*-phenylsulfamoyl)benzamide as a colourless glass (**21**, 53 mg, 0.13 mmol, 36% yield). ^{1}H NMR (500 MHz, Chloroform-*d*) δ 7.84 – 7.79 (m, 0.2H), 7.77 (d, *J* = 8.0 Hz, 1.9H), 7.52 (d, *J* = 8.5 Hz, 2.2H), 7.42 – 7.29 (m, 3.6H), 7.25 – 7.20 (m, 2.3H),

7.14 (app t, J = 7.4 Hz, 1.2H), 7.09 (d, J = 7.3 Hz, 1.84H), 7.04 (d, J = 7.5 Hz, 2.1H), 6.56 (s, 0.9H), 4.87 – 4.79 (m, 0.2H), 4.56 (s, 1.8H), 3.69 (t, J = 6.5 Hz, 1.8H), 3.45 – 3.34 (m, 0.2H), 2.78 (t, J = 6.4 Hz, 1.8H), 2.44 – 2.31 (m, 0.2H). Mixture of rotamers in 9:1 ratio. ACQUITY UPLC® BEH C18 1.7 µm: Rt = 1.69 min; m/z 420.1 $[M + H]^+$.

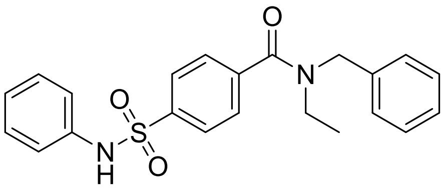

(**22**)

4-(Phenylsulfamoyl)benzoic acid (100.4 mg, 0.36 mmol), EDC·HCl (87.7 mg, 0.46 mmol) and HOBt (63.7 mg, 0.41 mmol) were suspended in dichloromethane (8 mL), the mixture degassed with nitrogen for 10 min and there after stirred for 1 h at rt. *N*-Benzylethanamine (79 µL, 0.54 mmol) was added *via* pipette and the mixture stirred overnight at rt. The reaction mixture was adsorbed onto silica, concentrated under reduced pressure and purified by automatic flash column chromatography (silica, 12 g, petrol:EtOAc; 100:0 to 0:100 over 25 CV), followed by a second round of column chromatography (silica, 12 g, CH_2Cl_2:MeOH; 100:0 to 99:1 over 30 CV) to give near-pure product. Finally, the impure desired product was dissolved in MeCN (2.0 mL) and purified by reverse-phase HPLC preparative chromatography (C_{18}, 20 × 150 mm, H_2O:MeCN; 90:10 to 80:20 over 25 min). The residue following concentration under reduced pressure was further dried in a vacuum oven at 40°C to afford *N*-benzyl-*N*-ethyl-4-(*N*-phenylsulfamoyl)benzamide as a colourless glass (**22**, 98.6 mg, 0.25 mmol, 69% yield). ^{1}H NMR (500 MHz, Methanol-d_4) δ 7.85 (d, J = 8.3 Hz, 1.1H), 7.76 (d, J = 8.0 Hz, 0.9H), 7.54 (d, J = 8.2 Hz, 1.1H), 7.48 (d, J = 8.0 Hz, 0.9H), 7.39 – 7.24 (m, 4.1H), 7.23 – 7.18 (m, 1.1H), 7.16 (t, J = 7.8 Hz, 0.9H), 7.13 – 6.99 (m, 3.9H), 4.76 (s, 1.1H), 4.41 (s, 0.9H), 3.51 (q, J = 7.1 Hz, 0.9H), 3.14 (q, J = 7.1 Hz, 1.1H), 1.19 (t, J = 7.1 Hz, 1.3H), 1.02 (t, J = 7.1 Hz, 1.7H). Mixture of rotamers in 1.3:1 ratio. ACQUITY UPLC® BEH C_{18} 1.7 µm: Rt = 1.76 min; m/z 395.1 $[M + H]^+$.

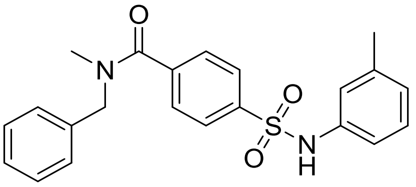

(**23**)

Step 1 Synthesis of methyl 4-(m-tolylsulfamoyl)benzoate

m-Toluidine (82 µL, 0.760 mmol) and 4-chlorosulfonyl-benzoic acid methyl ester (151.3 mg, 0.640 mmol) were dissolved in pyridine (3.5 mL, 43.27 mmol) and the mixture heated for 20 min at 100°C in a Biotage microwave. The reaction mixture was diluted with EtOAc (35 mL), washed with water (3 × 25 mL) and brine (2 × 25mL), the organic phase dried over $MgSO_4$, filtered and concentrated under reduced pressure to give the crude product, which was purified by automated flash column chromatography (CH_2Cl_2:MeOH; 100:0 to 99:1 over 15 min) to afford the product, methyl 4-(*m*-tolylsulfamoyl)benzoate as an off-white solid (141.4 mg, 0.440 mmol, 68% yield). ^{1}H NMR (500 MHz, Methanol-d_4) δ 8.08 (d, J = 8.1 Hz, 2H), 7.83 (d, J = 8.1 Hz, 2H), 7.08 app (t, J = 7.7 Hz, 1H), 6.91 – 6.84 (m, 3H), 3.91 (s, 3H), 2.23 (s, 3H). ACQUITY UPLC® BEH C_{18} 1.7 µm: Rt = 1.73 min; m/z 304.0 $[M-H]^-$.

Step 2 Synthesis of N-benzyl-N-methyl-4-(m-tolylsulfamoyl)benzamide

Methyl 4-(*m*-tolylsulfamoyl)benzoate (54.8 mg, 0.180 mmol) was dissolved in 1,2-dichloroethane (5 mL) and the mixture degassed with nitrogen for 10 min at 0°C. In a separate vessel, a solution of 1M triethylaluminium in hexanes (0.9 mL, 0.900 mmol) and benzylmethylamine (0.12 mL, 0.930 mmol) were added to degassed, ice-cold 1,2-dichloroethane (5 mL). The mixture containing triethylaluminium was warmed to rt and transferred *via* syringe to the solution of methyl 4-(m-tolylsulfamoyl)benzoate. The reaction mixture was then allowed to warm to rt and subsequently heated to 80°C for 18 h. The reaction mixture was cooled to 0°C and quenched with 1M HCl until pH 1 was observed. The mixture was diluted with CH_2Cl_2 (35 mL), the organic phase washed with 1M HCl (3 × 20 mL), water (2 × 20mL) and brine (1 × 20 mL). The organic extract was dried over $MgSO_4$, filtered and concentrated under reduced pressure to give the crude product, which was purified by reverse phase automatic preparative HPLC (C_{18}, 250 mm × 10 mm; H_2O:MeOH; 90:10 to 0:100 over 20 min) to give the desired product, which was dried at 40°C in a vacuum oven to afford the product, *N*-benzyl-*N*-methyl-4-(*m*-tolylsulfamoyl)benzamide as a colourless glass (**24**, 60 mg, 0.145 mmol, 81% yield). ^{1}H NMR (500 MHz, Methanol-d_4) δ 7.84 (d, *J* = 8.0 Hz, 1.1H), 7.78 (d, *J* = 8.0 Hz, 0.9H), 7.56 (d, *J* = 8.0 Hz, 1.1H), 7.52 (d, *J* = 8.0 Hz, 0.9H), 7.40 – 7.26 (m, 4.3H), 7.14 – 7.01 (m, 2H), 6.92 – 6.82 (m, 3H), 4.74 (s, 1.1H), 4.41 (s, 0.9H), 3.02 (s, 1.3H), 2.81 (s, 1.6H), 2.22 (s, 1.6H), 2.17 (s, 1.3H). Mixture of rotamers in 0.8:1 ratio. ACQUITY UPLC® BEH C_{18} 1.7µm: Rt = 1.75 min; m/z 395.1 $[M+H]^+$.

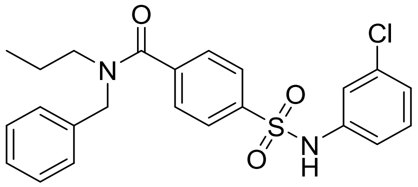

(**24**)

Step 1 – Synthesis of methyl 4-[(3-chlorophenyl)sulfamoyl]benzoate

3-Chloroaniline (47 µL, 0.450 mmol) and 4-chlorosulfonyl-benzoic acid methyl ester (100 mg, 0.430 mmol) were dissolved in pyridine (2.5 mL, 30.91 mmol) and the mixture heated for 30 min at 100°C in a Biotage microwave. The mixture was diluted with CH_2Cl_2 (25 mL), washed with water (5 × 25 mL), brine (3 × 25mL), the organic phase dried over $MgSO_4$, filtered and concentrated under reduced pressure. The isolated solid was dried in a vacuum oven at 45˚C for 1.5 h to afford the product, methyl 4-[(3-chlorophenyl)sulfamoyl]benzoate as a yellow solid (119 mg, 0.347 mmol, 81% yield). ^{1}H NMR (500 MHz, Chloroform-*d*) δ 8.12 (d, *J* = 7.8 Hz, 2H), 7.85 (d, *J* = 7.8 Hz, 2H), 7.21 – 7.14 (m, 1H), 7.14 – 7.07 (m, 2H), 6.95 (d, *J* = 8.0 Hz, 1H), 6.68 (s, 1H), 3.94 (s, 3H). ACQUITY UPLC® BEH C_{18} 1.7 µm: Rt = 1.70 min; m/z 323.9 $[M-H]^-$.

Step 2 – Synthesis of N-benzyl-4-[(3-chlorophenyl)sulfamoyl]-N-propyl-benzamide as a colourless glass

Methyl 4-[(3-chlorophenyl)sulfamoyl]benzoate (100 mg, 0.310 mmol) was dissolved in 1,2-dichloroethane (5 mL) and the solution degassed with nitrogen for 10 min at 0˚C. In a separate vessel, solution of 1M triethylaluminium in hexanes (1.53 mL, 1.53 mmol) and *N*-benzyl-*N*-propylamine (229 mg, 1.53 mmol) were added to degassed, ice-cold 1,2-dichloroethane (5 mL).. The mixture containing triethylaluminium was warmed to rt and transferred *via* syringe to the solution of methyl 4-[(3-chlorophenyl)sulfamoyl]benzoate. The reaction mixture was warmed to rt and subsequently heated to 80˚C for 18 h. The reaction mixture was then allowed to warm to rt and subsequently heated to 80°C for 18 h. The reaction mixture was cooled to 0°C and quenched with 1M HCl until pH 1 was observed. The mixture was diluted with CH_2Cl_2 (35 mL), the organic phase washed with 1M HCl (3 × 20 mL), water (2 × 20mL) and brine (1 × 20 mL). The organic extract was dried over

$MgSO_4$, filtered and concentrated under reduced pressure to give the crude product, which was purified by reverse phase automatic preparative HPLC (C_{18}, 250 mm × 10 mm; H_2O:MeOH; 90:10 to 0:100 over 25 min) to give the desired product, which was dried at 40°C in a vacuum oven to afford the product, *N*-benzyl-4-[(3-chlorophenyl)sulfamoyl]-*N*-propyl-benzamide as a colourless glass (**25**, 104 mg,0.222 mmol, 72% yield). ^{1}H NMR (500 MHz, Methanol-d_4) δ 7.88 (d, *J* = 7.9 Hz, 1.1H), 7.79 (d, *J* = 7.9 Hz, 0.9H), 7.55 (d, *J* = 7.9 Hz, 1.0H), 7.49 (d, *J* = 8.0 Hz, 0.9H), 7.39 – 7.33 (m, 1.9H), 7.33 – 7.22 (m, 1.8H), 7.22 – 7.12 (m, 1.0H), 7.12 – 7.06 (m, 1.27H), 7.06 – 6.96 (m, 2.1H), 4.76 (s, 1.1H), 4.42 (s, 0.9H), 3.44 (t, *J* = 7.6 Hz, 0.9H), 3.04 (t, *J* = 7.8 Hz, 1.1H), 1.67 (app sext, *J* = 7.6 Hz, 0.9H), 1.47 (app sext, *J* = 7.6 Hz, 1.1H), 0.94 (t, *J* = 7.5 Hz, 1.3H), 0.62 (t, *J* = 7.4 Hz, 1.6H). Mixture of rotamers in 1:1.1 ratio. ACQUITY UPLC® BEH C_{18} 1.7 µm: Rt = 1.78 min; m/z 443.0 $[M+H]^+$.

FRAX-486 (**1**)

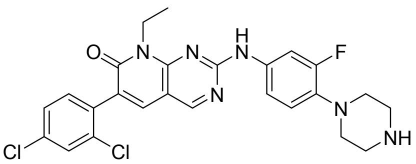

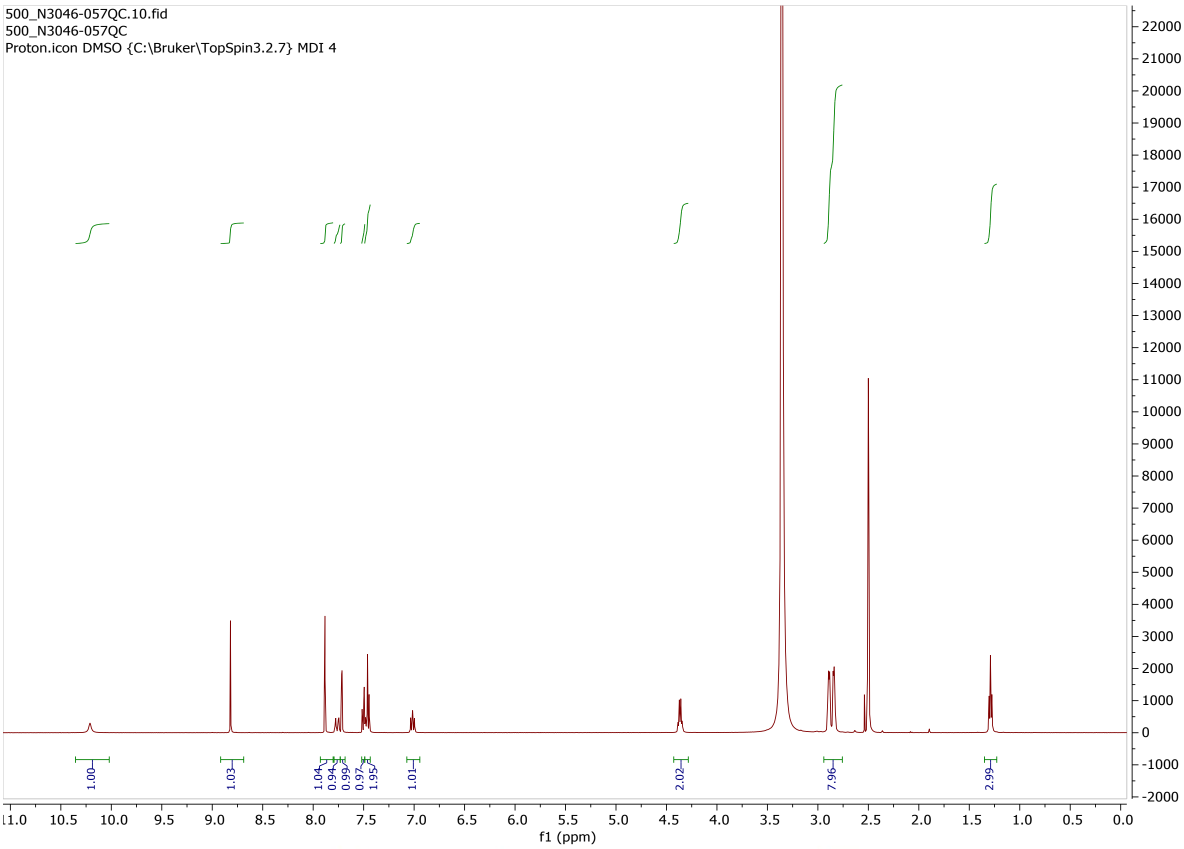

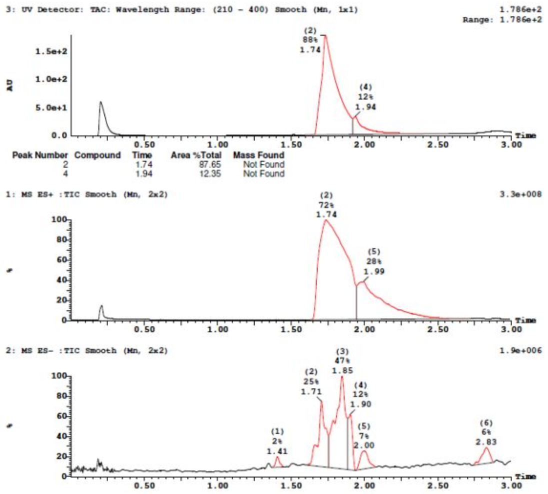

Peak ID	Compound	Time	Mass Found
2		1.74	Not Found

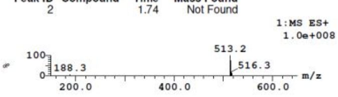

BMS-3 (**2**)

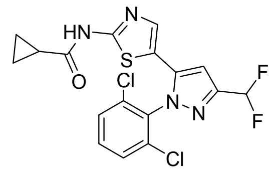

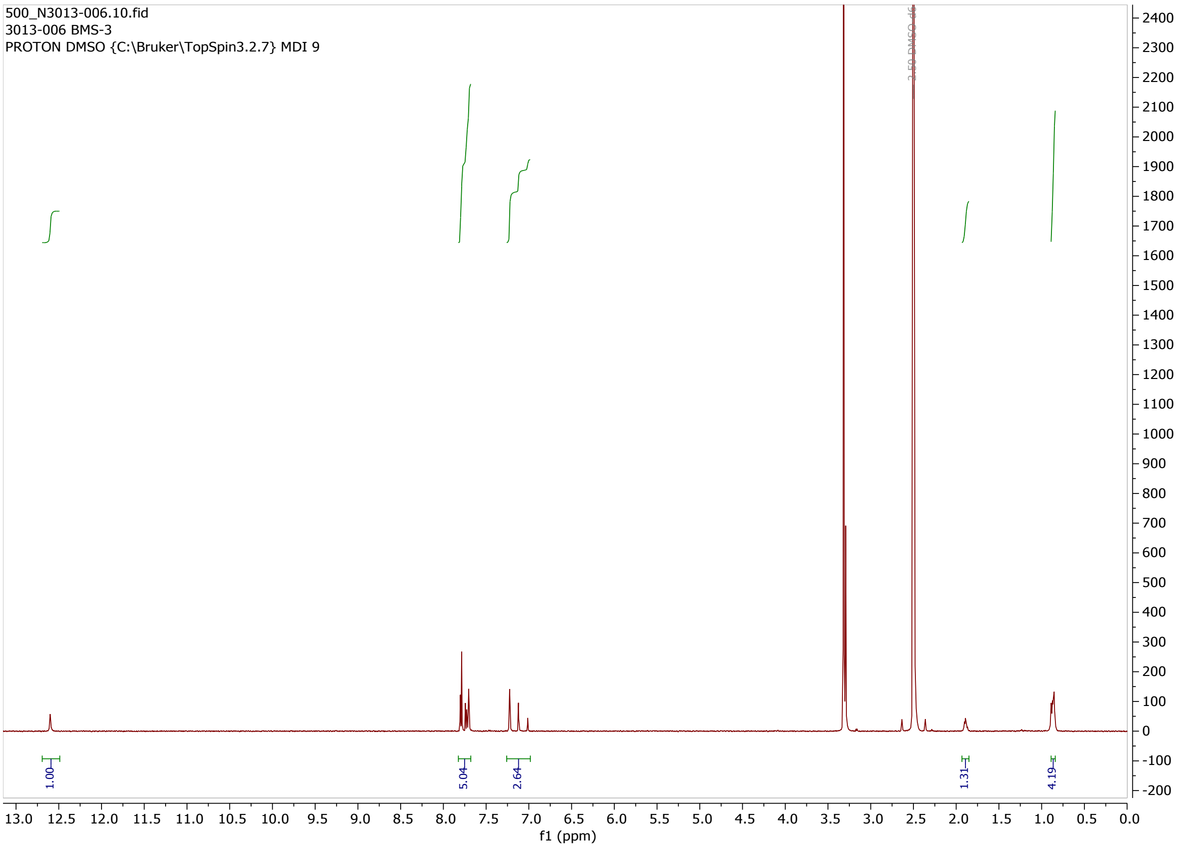

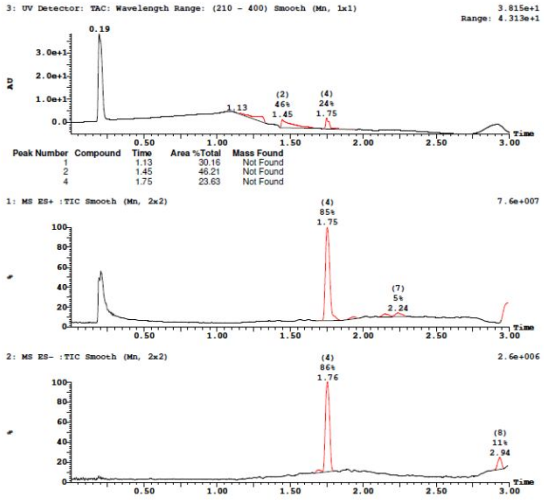

Peak ID	Compound	Time	Mass Found
4		1.75	Not Found

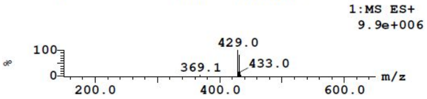

BMS-4 (**3**)

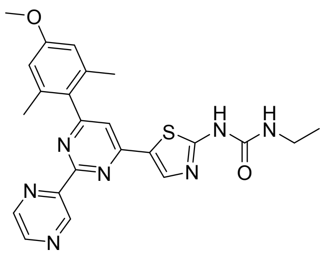

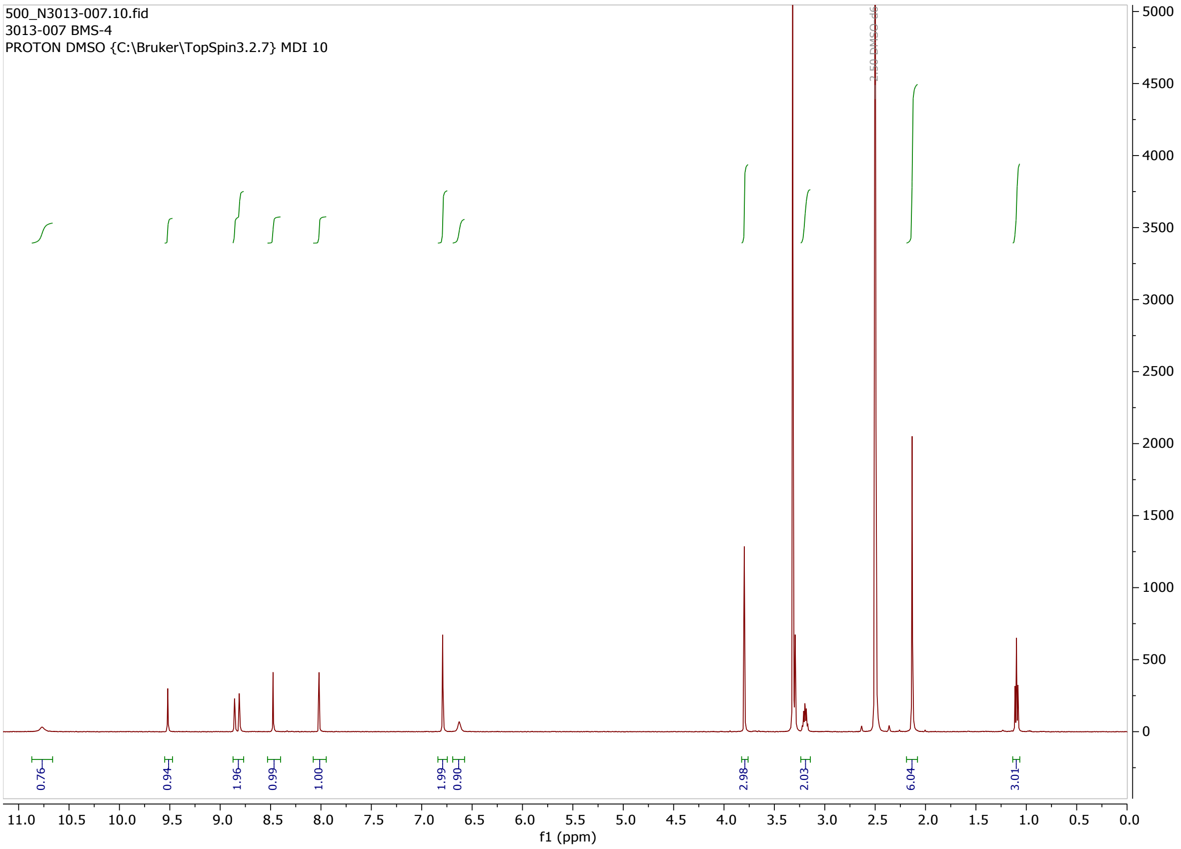

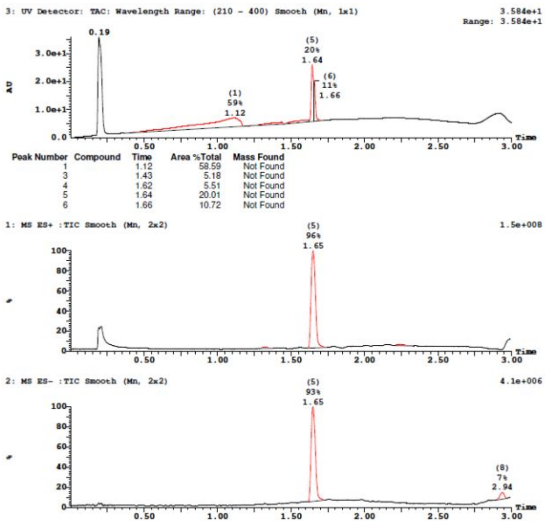

Peak Number	Compound	Time	Area %Total	Mass Found
1		1.12	58.59	Not Found
3		1.43	5.18	Not Found
4		1.62	5.51	Not Found
5		1.64	20.01	Not Found
6		1.66	10.72	Not Found

Peak ID	Compound	Time	Mass Found
4		1.62	Not Found

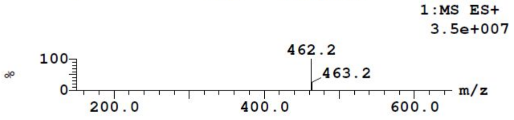

BMS-5/LIMKi3 (**4**)

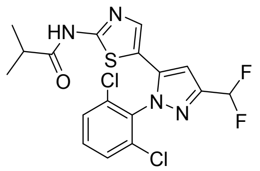

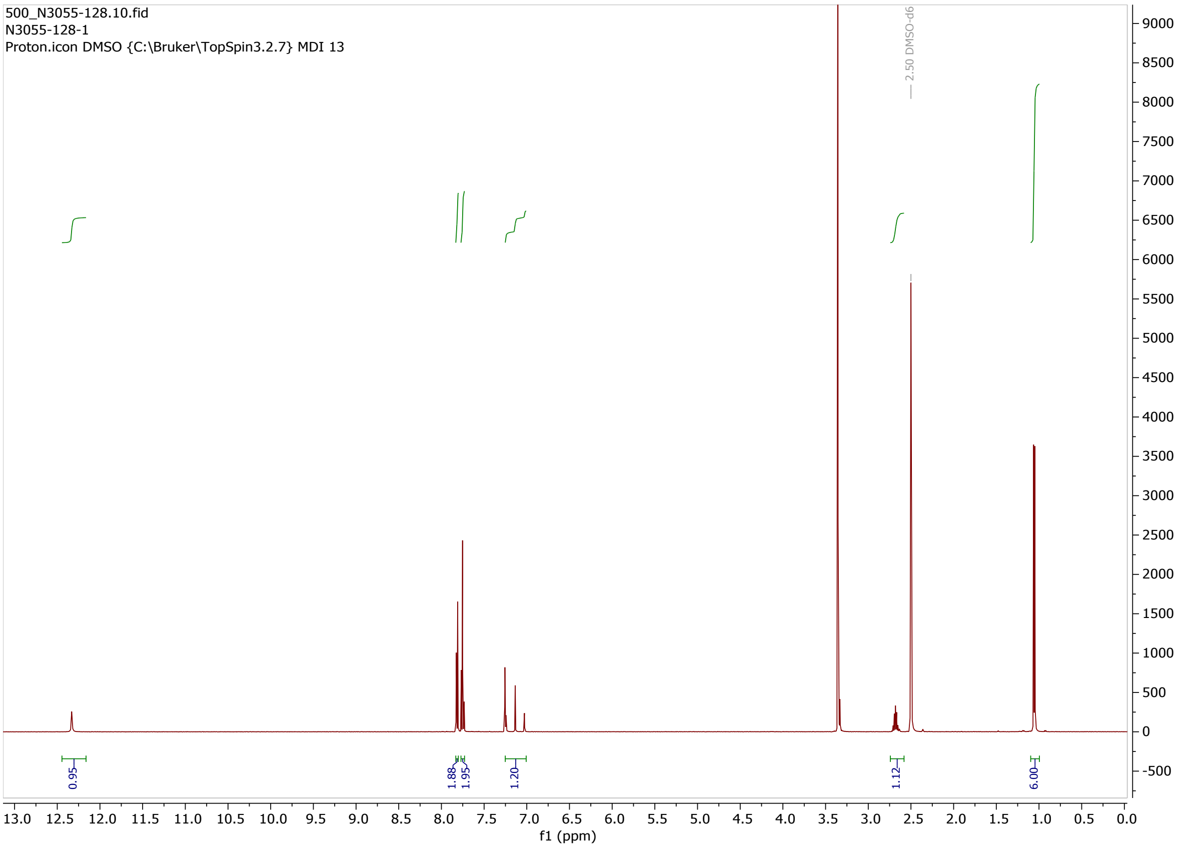

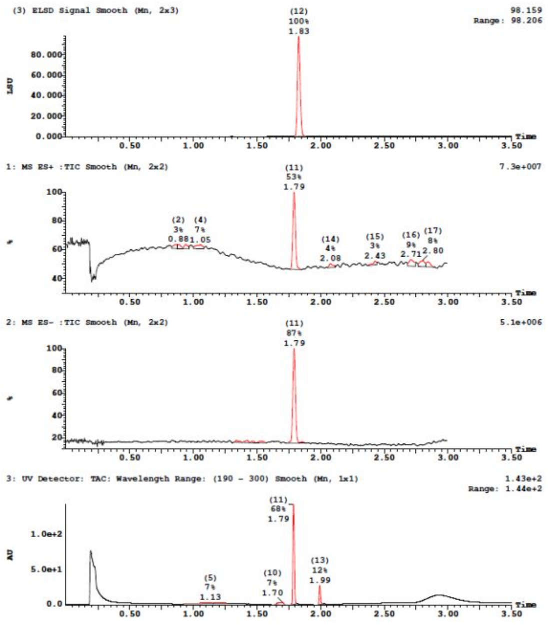

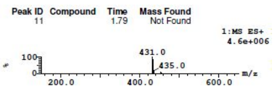

T-56-LIMKi (**5**)

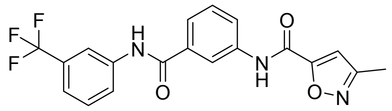

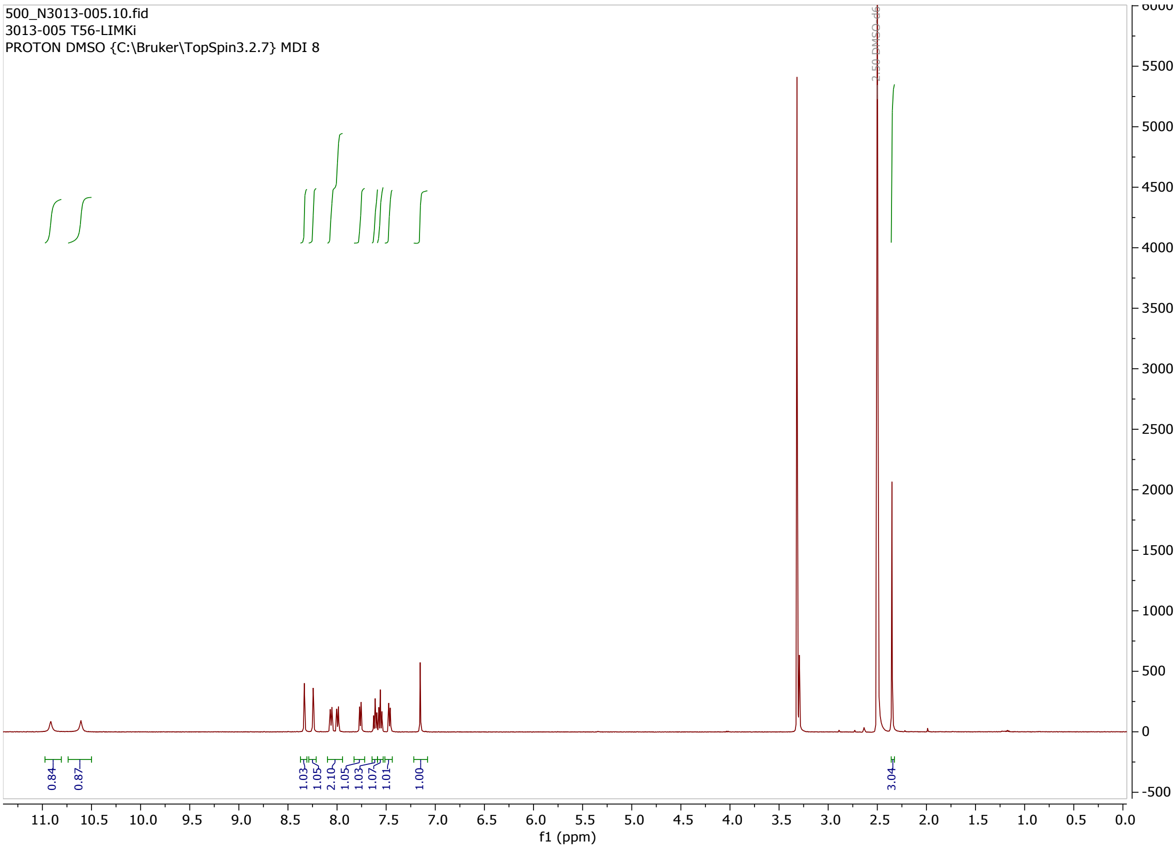

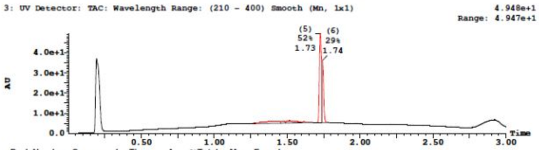

Peak Number	Compound	Time	Area %Total	Mass Found
1		1.39	9.82	Not Found
2		1.48	1.93	Not Found
3		1.51	6.66	Not Found
4		1.62	1.42	Not Found
5		1.73	51.58	Not Found
6		1.74	28.59	Not Found

Peak ID	Compound	Time	Mass Found
6		1.74	Not Found

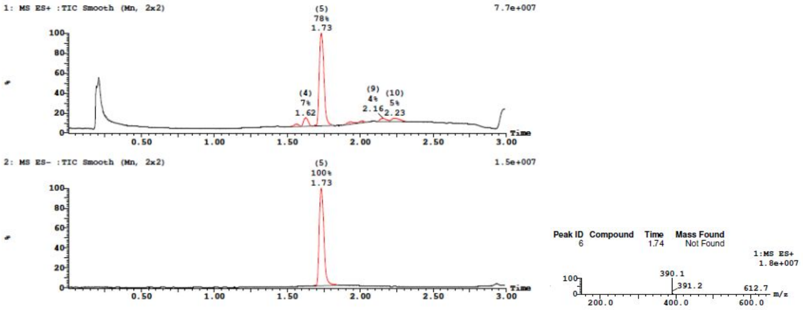

22j (**6**)

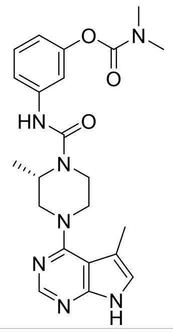

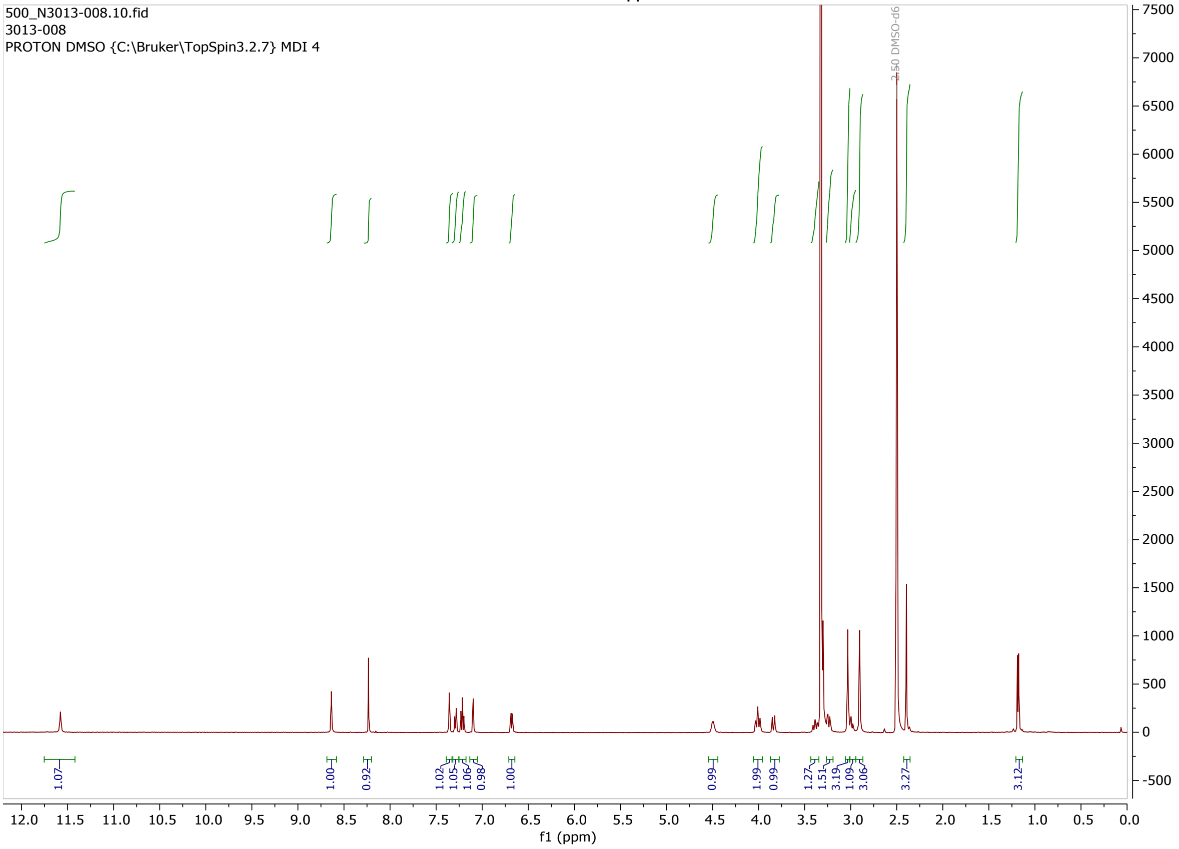

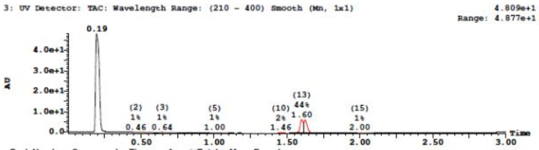

Peak Number	Compound	Time	Area %Total	Mass Found
2		0.46	1.39	Not Found
3		0.64	1.43	Not Found
5		1.00	1.26	Not Found
10		1.46	2.08	Not Found
13		1.60	44.02	Not Found
14		1.63	48.69	Not Found
15		2.00	1.13	Not Found

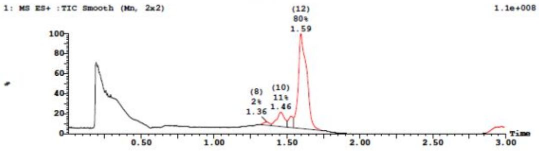

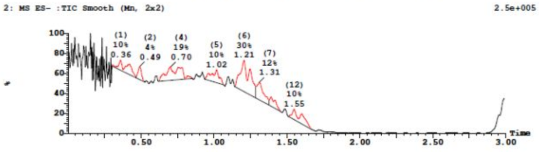

Peak ID	Compound	Time	Mass Found
12		1.59	Not Found

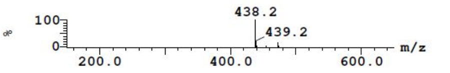

LX-7101 (**7**)

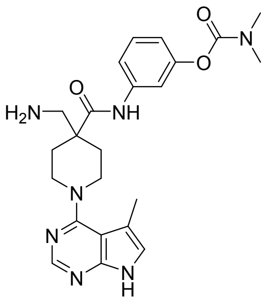

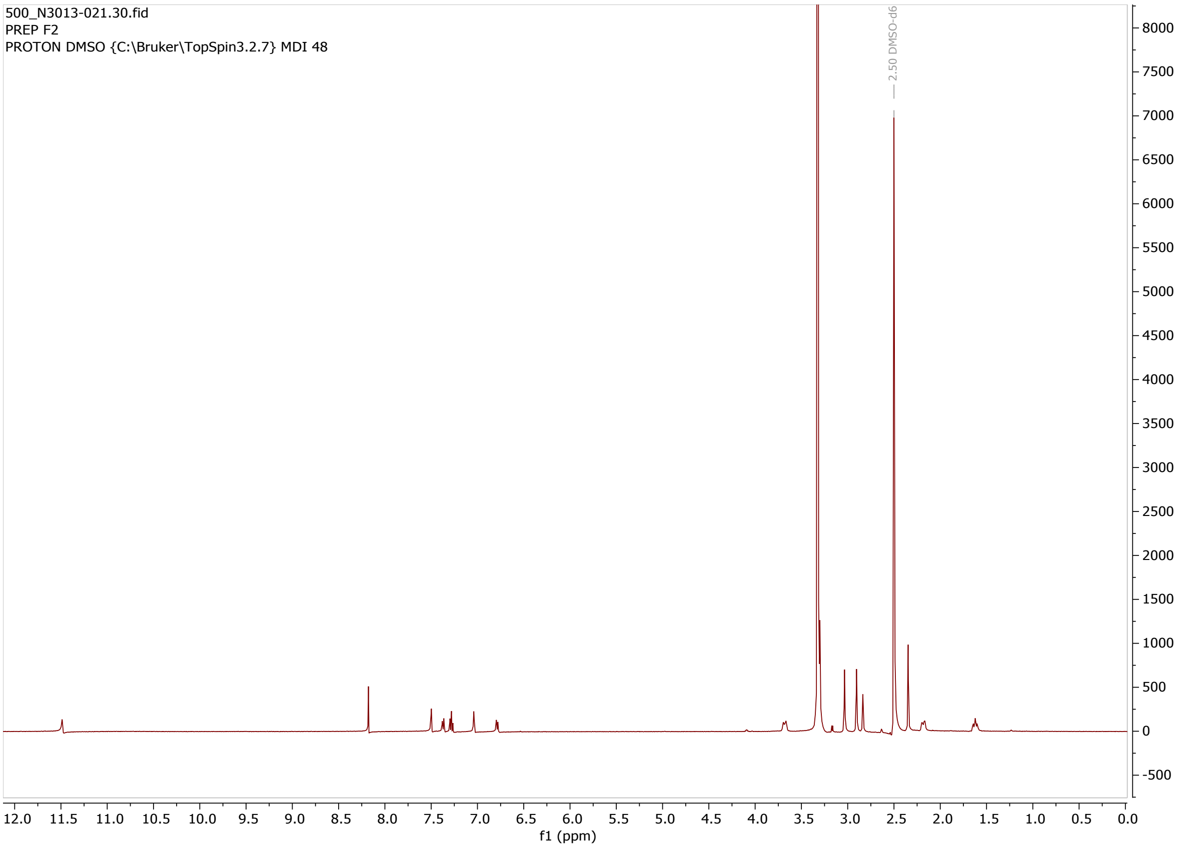

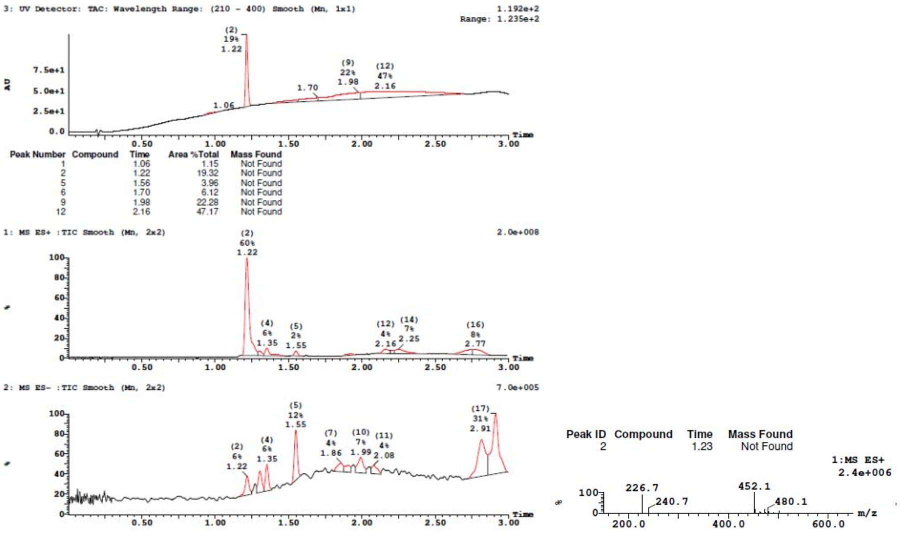

SR-7826 (**8**)

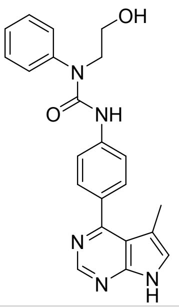
OH
N
O
NH
N
N
N
H

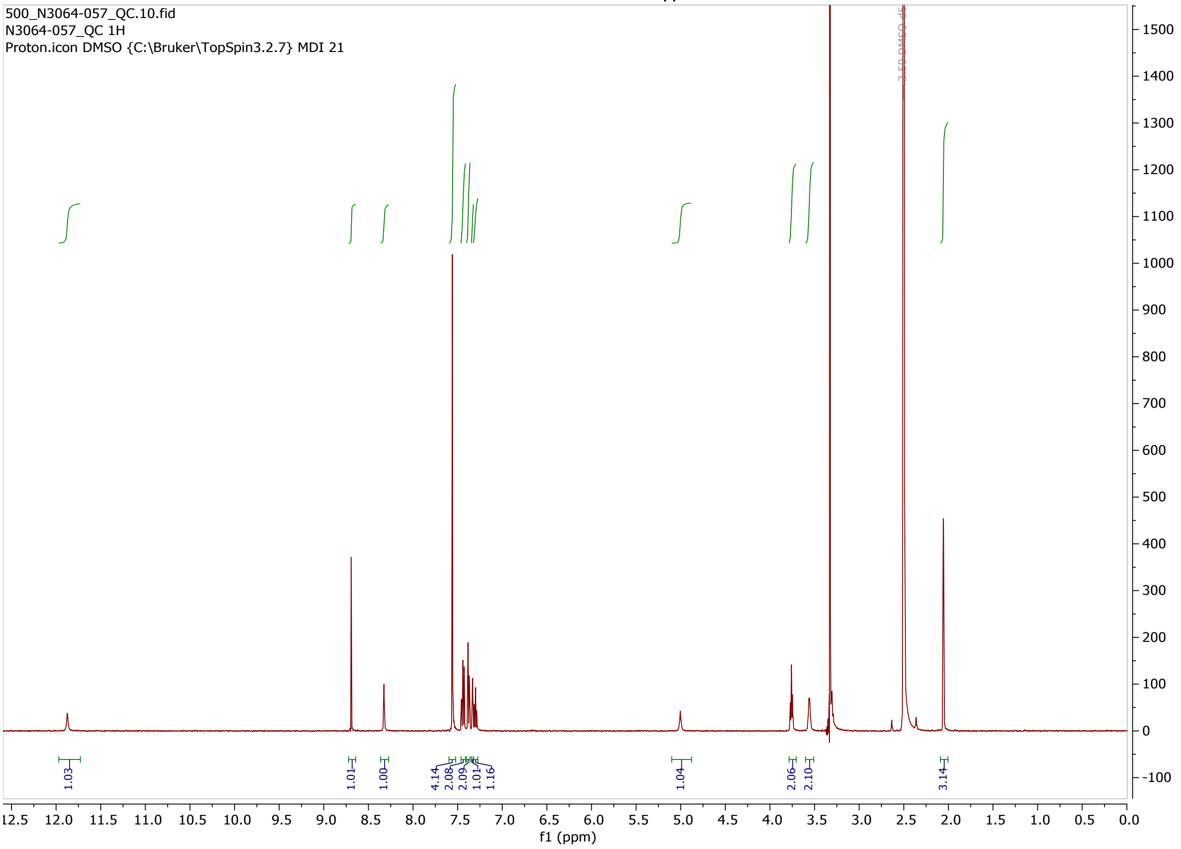
500_N3064-057_QC.10.fid
N3064-057_QC 1H
Proton.icon DMSO {C:\Bruker\TopSpin3.2.7} MDI 21
1.03
1.01
1.00
4.14
2.08
2.09
1.01
1.16
1.04
2.06
2.10
3.14
f1 (ppm)

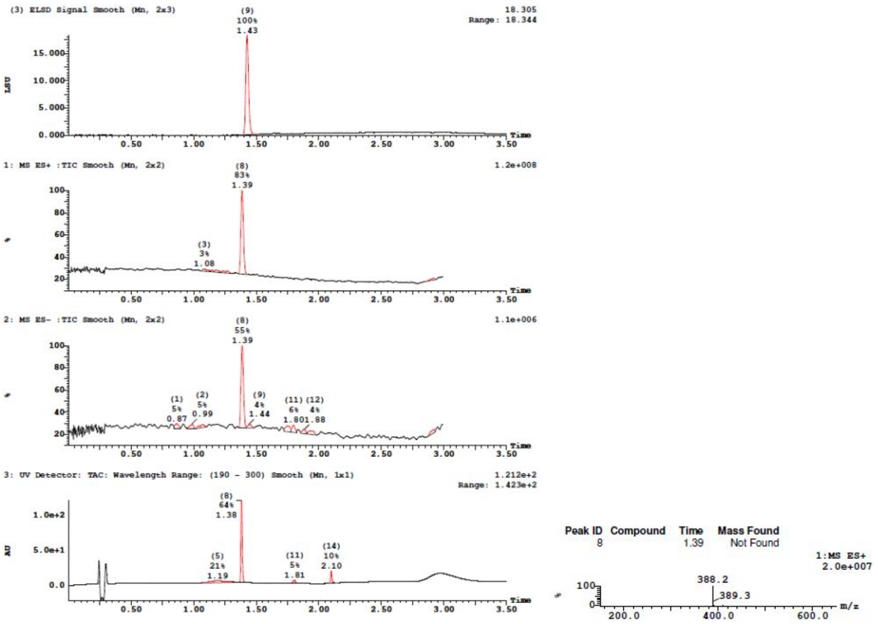
(3) ELSD Signal Smooth (Mn, 2x3)
1: MS ES+ :TIC Smooth (Mn, 2x2)
2: MS ES- :TIC Smooth (Mn, 2x2)
3: UV Detector: TAC: Wavelength Range: (190 - 300) Smooth (Mn, 1x1)
Peak ID Compound Time Mass Found
8 1.39 Not Found
1:MS ES+
388.2
389.3

TH-257 (**9**)

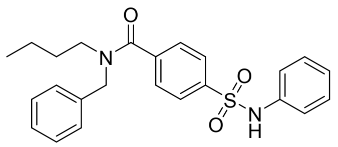

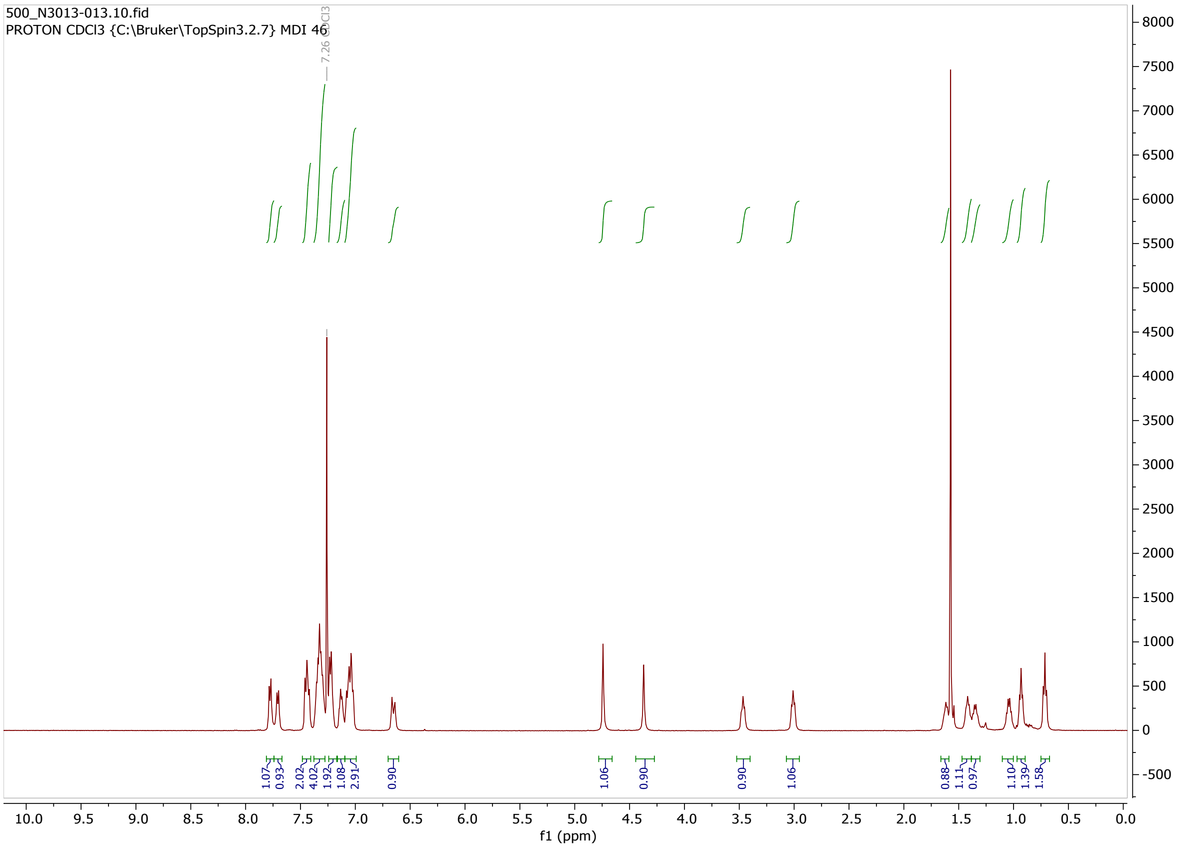

500_N3013-013.10.fid
PROTON CDCl3 {C:\Bruker\TopSpin3.2.7} MDI 46
f1 (ppm)

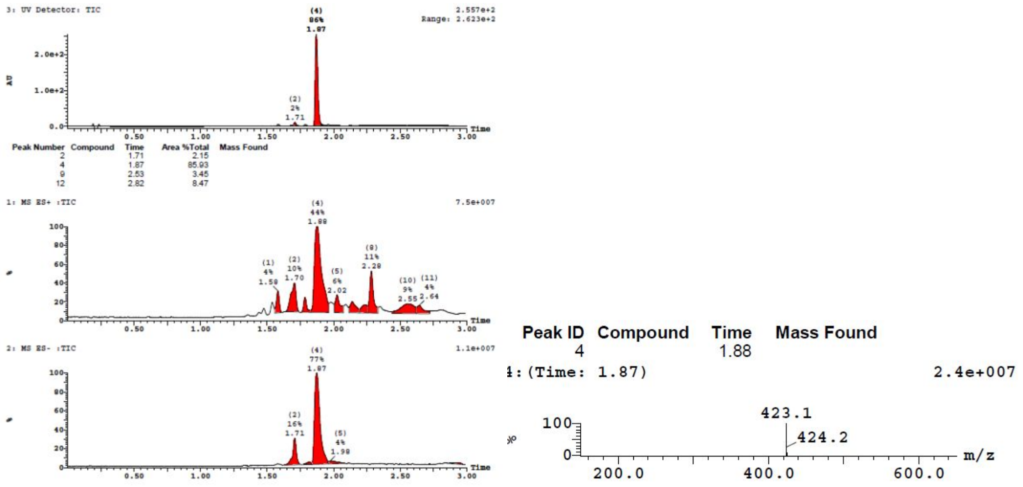

3: UV Detector: TIC
1: MS ES+ :TIC
2: MS ES- :TIC
Peak ID Compound Time Mass Found
4 1.88
423.1
424.2
m/z

TH-470 (**10**)

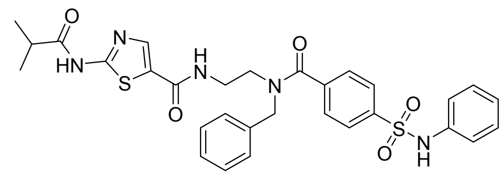

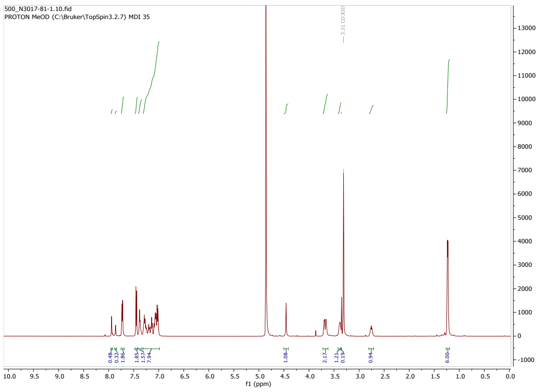

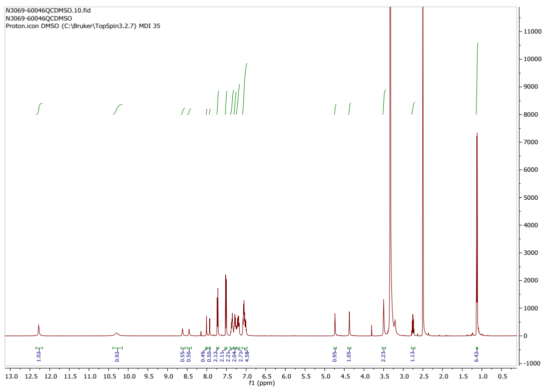

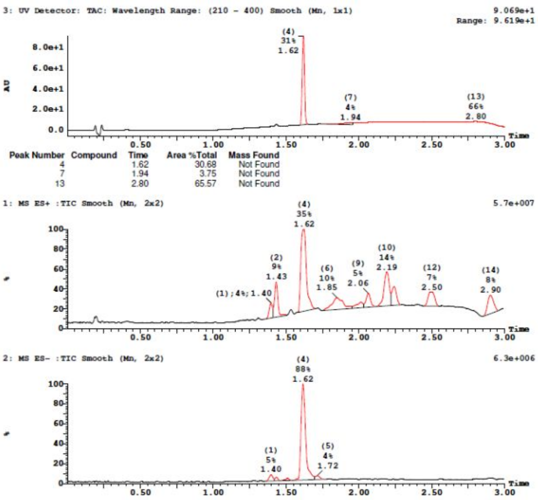

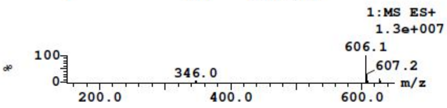

PHA-680632 (**11**)

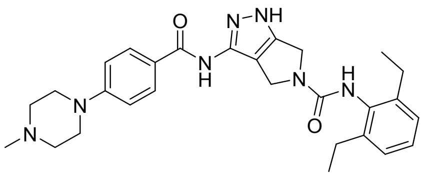
O
N–NH
N
H
N
N
H
N
O
N

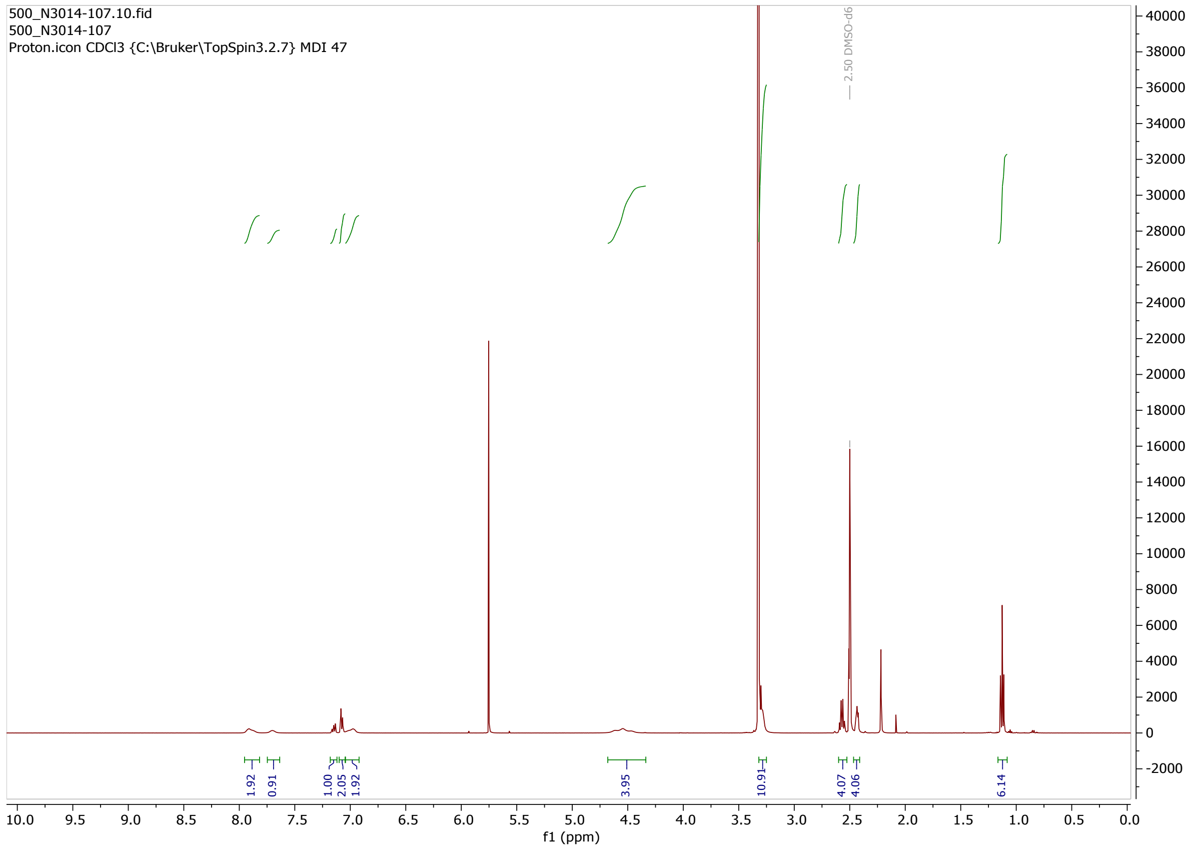
500_N3014-107.10.fid
500_N3014-107
Proton.icon CDCl3 {C:\Bruker\TopSpin3.2.7} MDI 47
2.50 DMSO-d6
1.92
0.91
1.00
2.05
1.92
3.95
10.91
4.07
4.06
6.14
f1 (ppm)

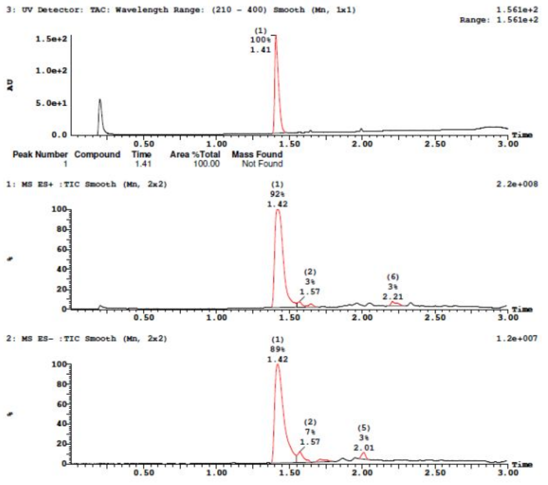
3: UV Detector: TAC: Wavelength Range: (210 - 400) Smooth (Mn, 1x1)
1.561e+2
Range: 1.561e+2
AU
Peak Number Compound Time Area %Total Mass Found
1 1.41 100.00 Not Found
1: MS ES+ :TIC Smooth (Mn, 2x2)
2.2e+008
2: MS ES- :TIC Smooth (Mn, 2x2)
1.2e+007
Time

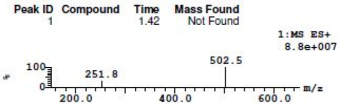
Peak ID Compound Time Mass Found
1 1.42 Not Found
1:MS ES+
8.8e+007
502.5
251.8
m/z

AZ-960 (**12**)

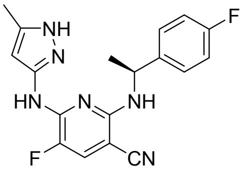
NH
N
HN
N
NH
F
F
CN

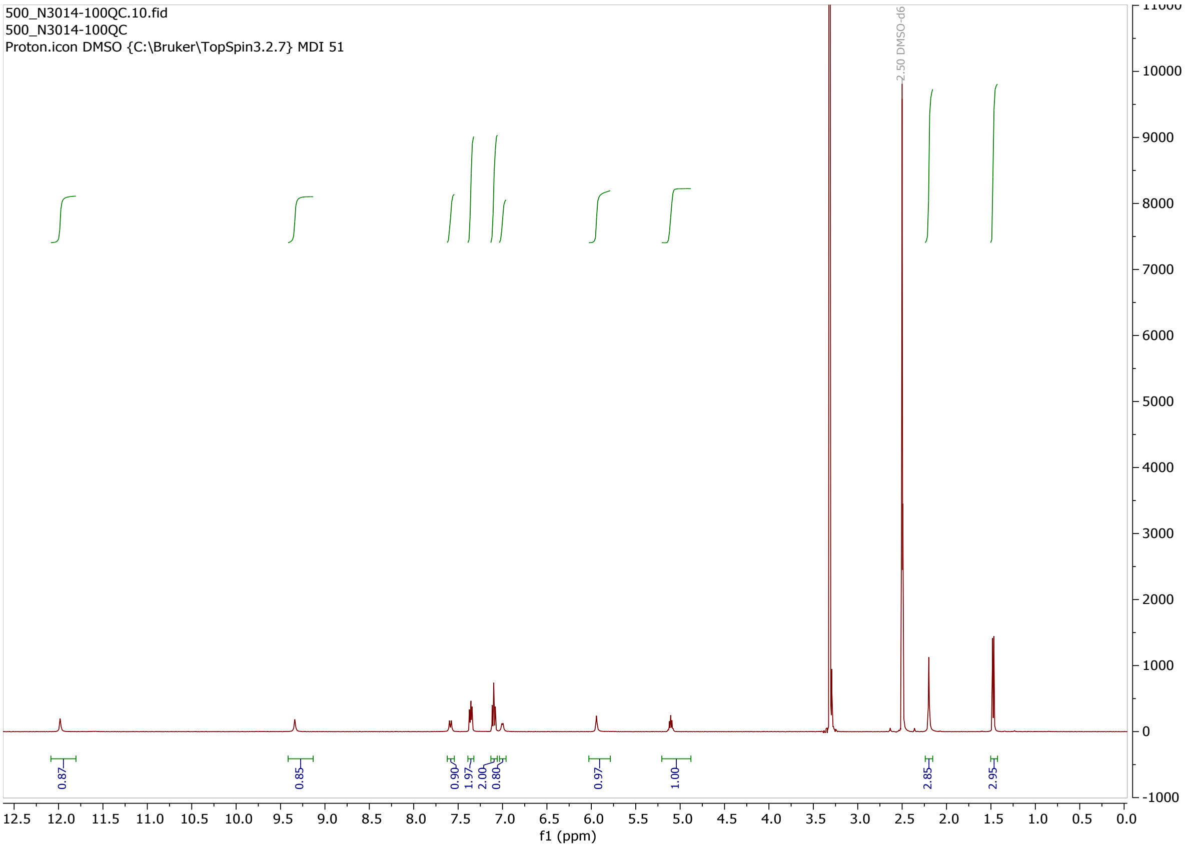
500_N3014-100QC.10.fid
500_N3014-100QC
Proton.icon DMSO {C:\Bruker\TopSpin3.2.7} MDI 51
2.50 DMSO-d6
0.87
0.85
0.90
1.97
2.00
0.80
0.97
1.00
2.85
2.95
f1 (ppm)

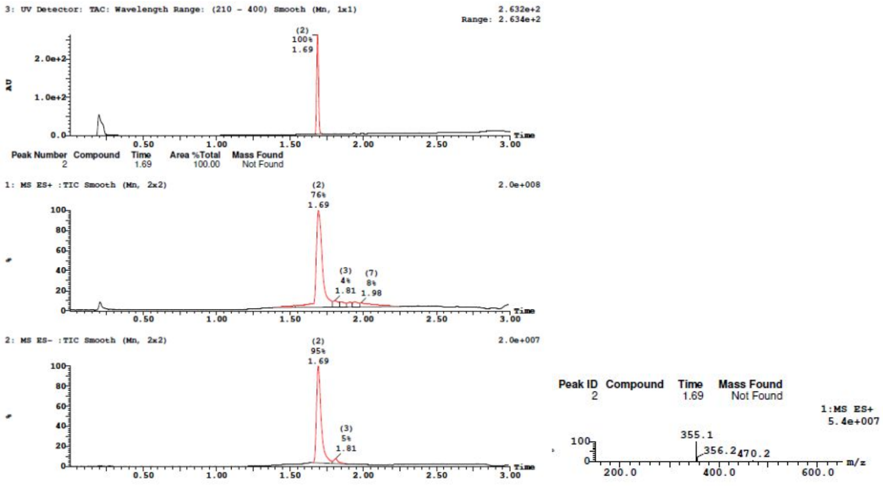
3: UV Detector: TAC: Wavelength Range: (210 - 400) Smooth (Mn, 1x1)
2.632e+2
Range: 2.634e+2
(2)
100%
1.69
Peak Number Compound Time Area %Total Mass Found
2 1.69 100.00 Not Found
1: MS ES+ :TIC Smooth (Mn, 2x2)
2.0e+008
(2)
76%
1.69
(3)
4%
1.81
(7)
8%
1.98
2: MS ES- :TIC Smooth (Mn, 2x2)
2.0e+007
(2)
95%
1.69
(3)
5%
1.81
Peak ID Compound Time Mass Found
2 1.69 Not Found
1:MS ES+
5.4e+007
355.1
356.2
470.2
m/z

Gandotinib (**13**)

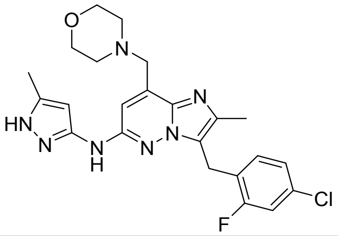

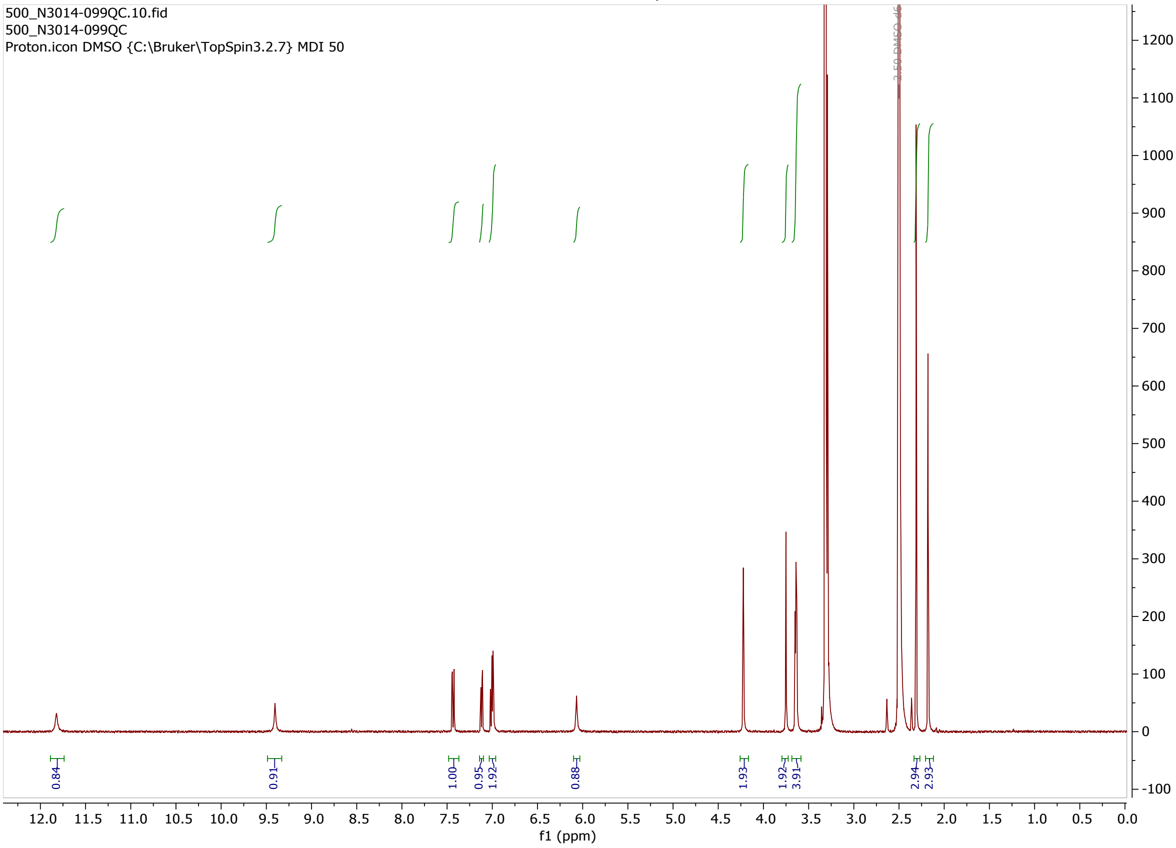

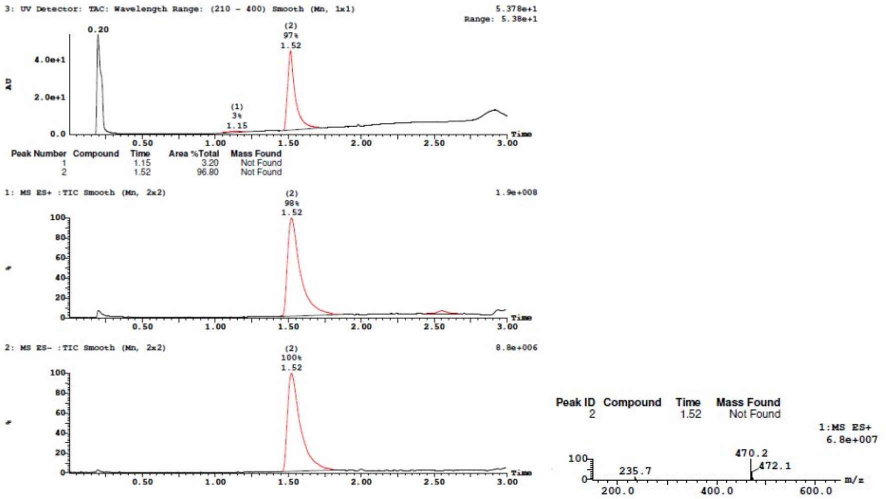

CHIR-98014 (**14**)

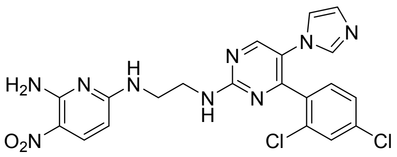

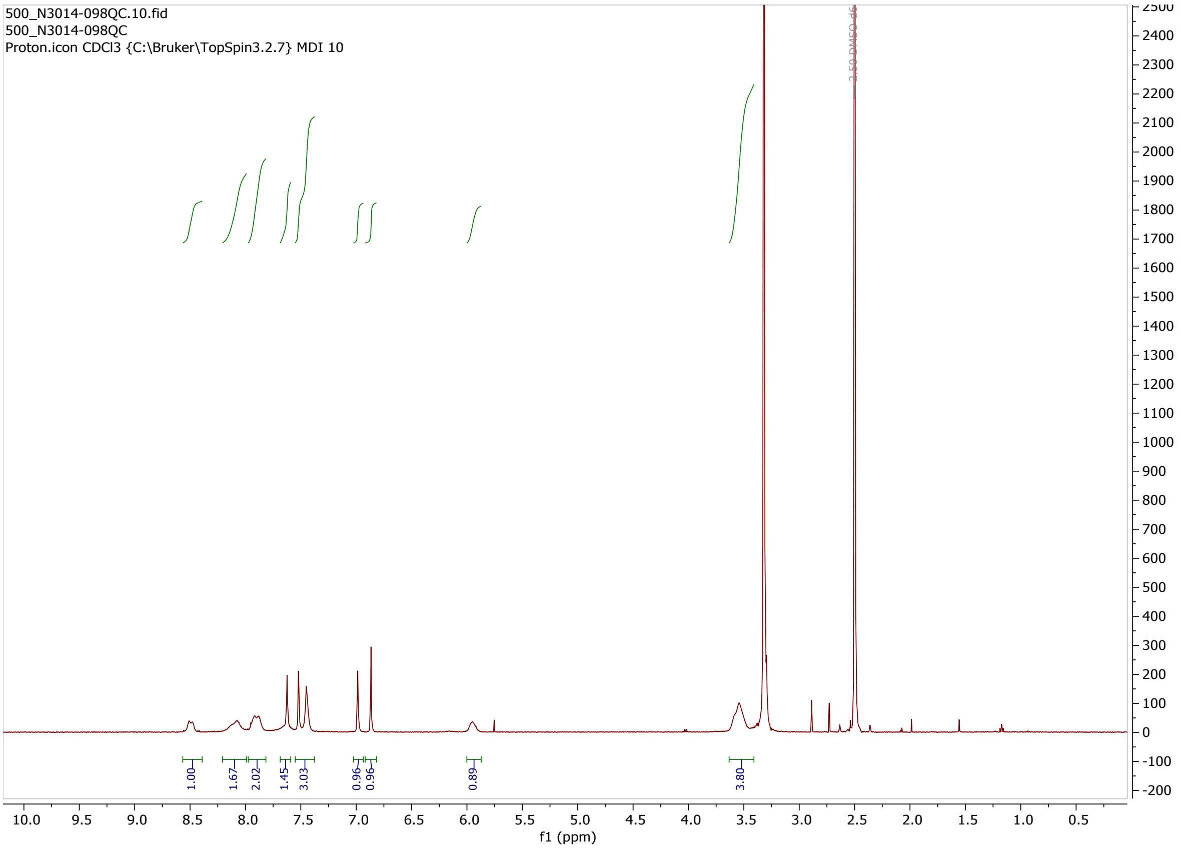

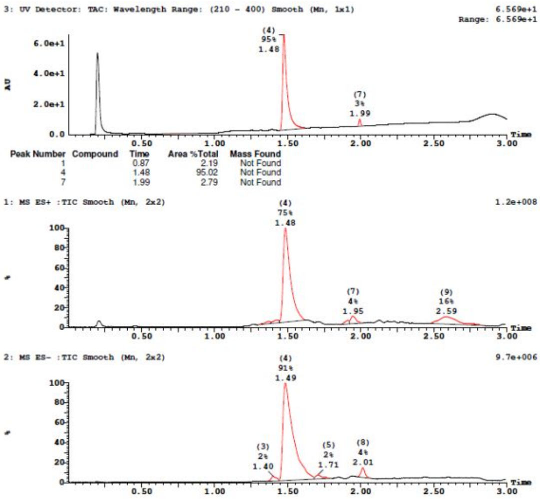

Peak Number	Compound	Time	Area %Total	Mass Found
1		0.87	2.19	Not Found
4		1.48	95.02	Not Found
7		1.99	2.79	Not Found

Peak ID	Compound	Time	Mass Found
4		1.48	Not Found

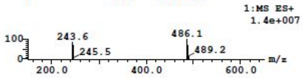

Dasatinib (**15**)

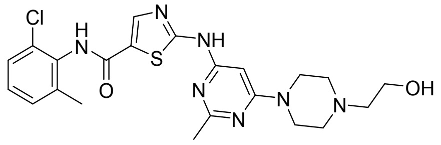

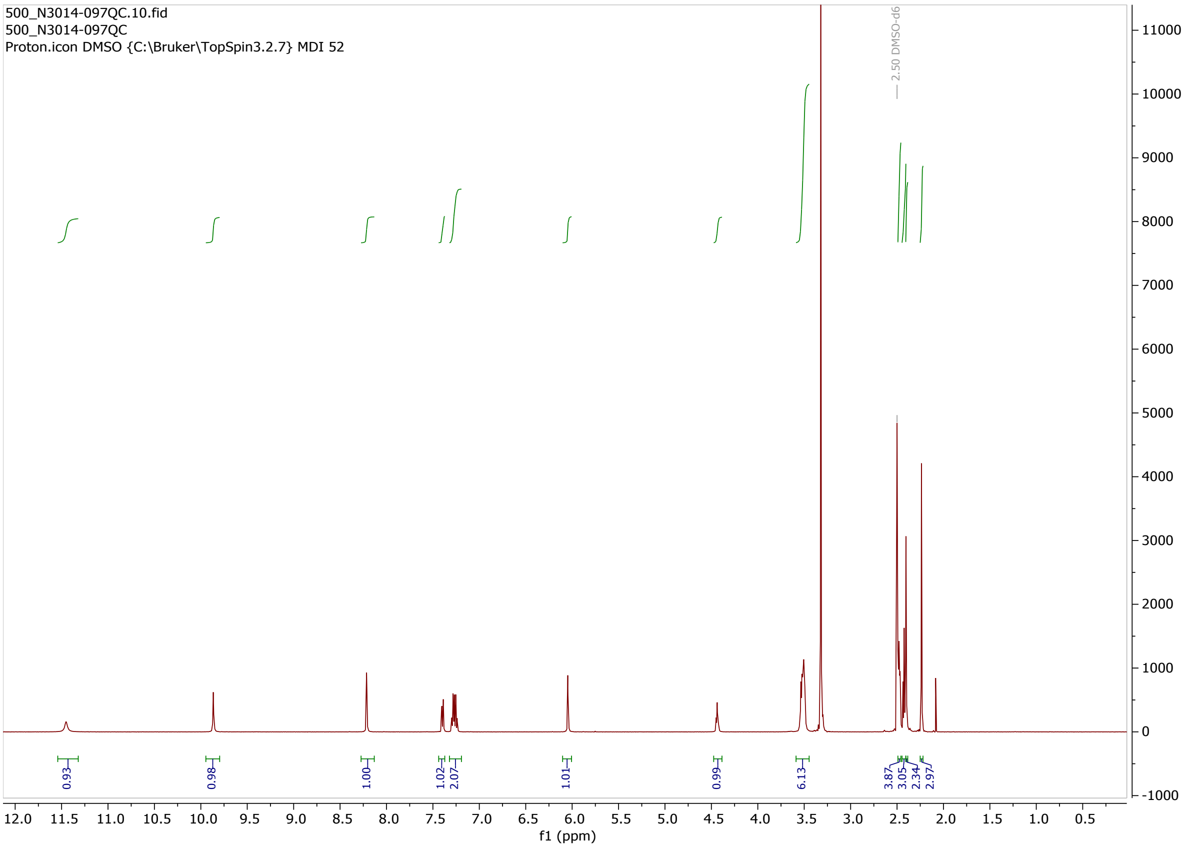

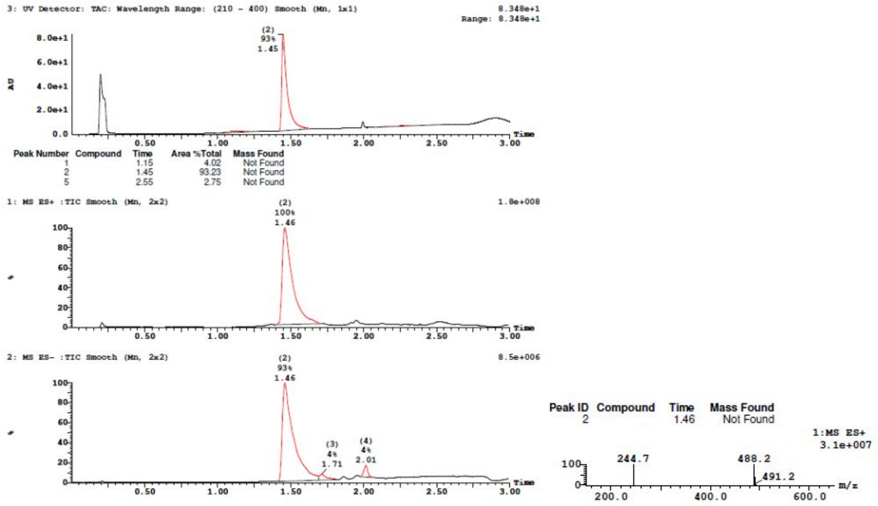

PF-477736 (**16**)

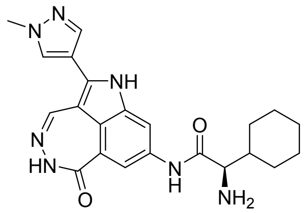

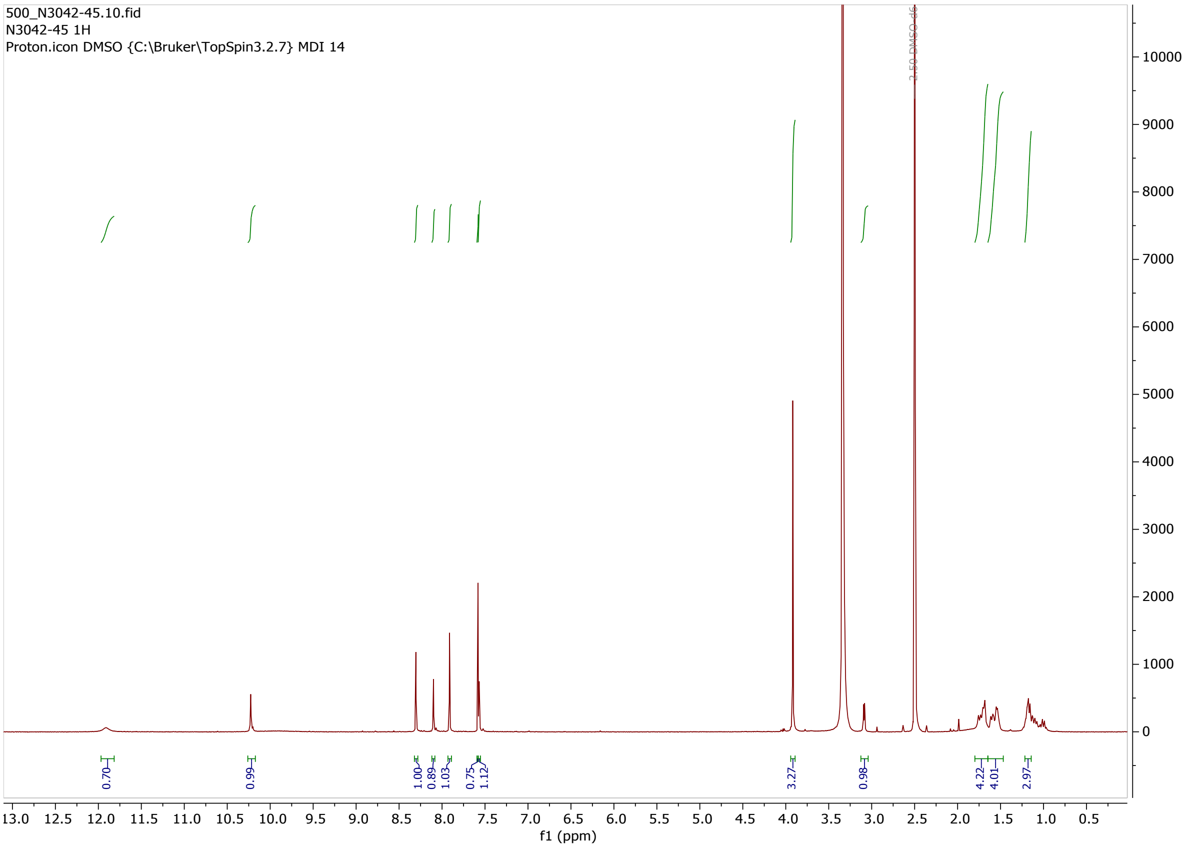

500_N3042-45.10.fid
N3042-45 1H
Proton.icon DMSO {C:\Bruker\TopSpin3.2.7} MDI 14
f1 (ppm)

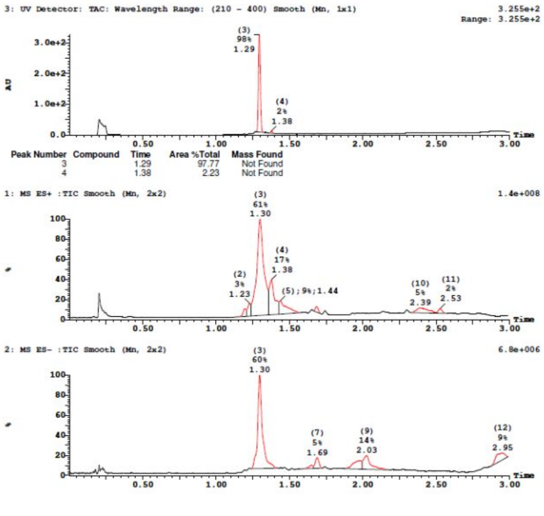

3: UV Detector: TAC: Wavelength Range: (210 - 400) Smooth (Mn, 1x1)
Peak Number Compound Time Area %Total Mass Found
3 1.29 97.77 Not Found
4 1.38 2.23 Not Found
1: MS ES+ :TIC Smooth (Mn, 2x2)
2: MS ES- :TIC Smooth (Mn, 2x2)

LIJTF500025 (**17**)

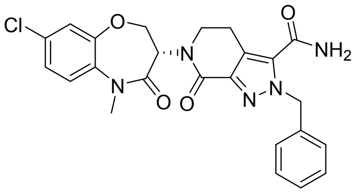

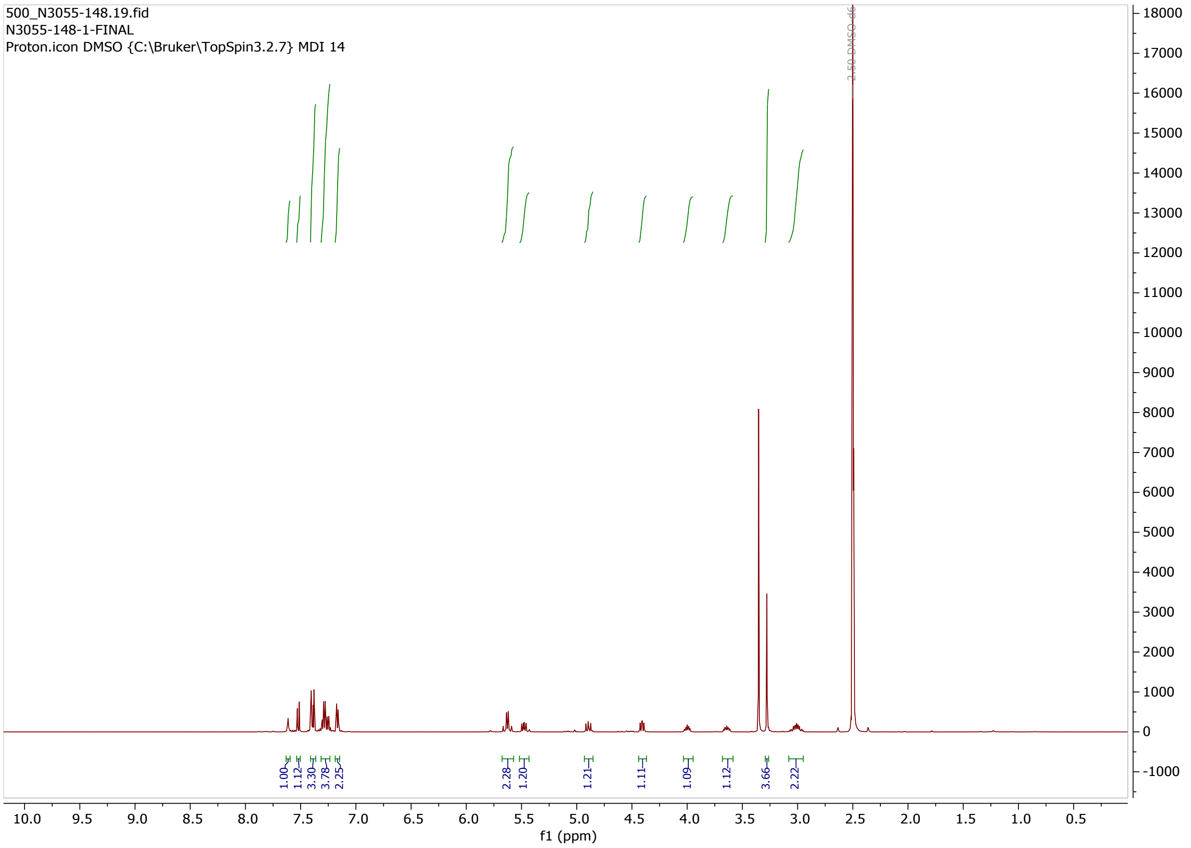

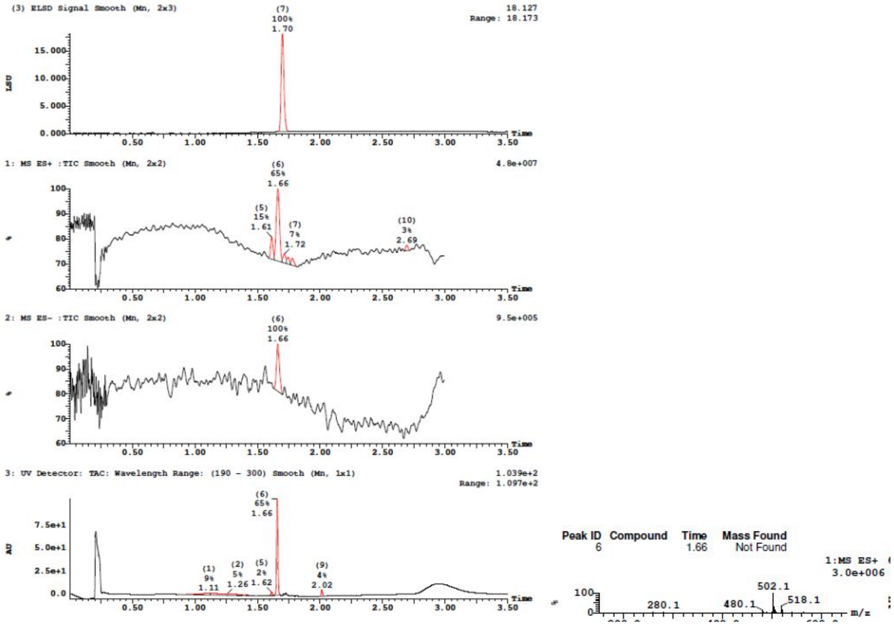

Peak ID	Compound	Time	Mass Found
6		1.66	Not Found

MDI-60021 (**21**)

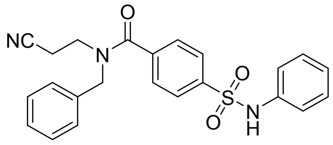

NC
N
O
O
S
O
N
H

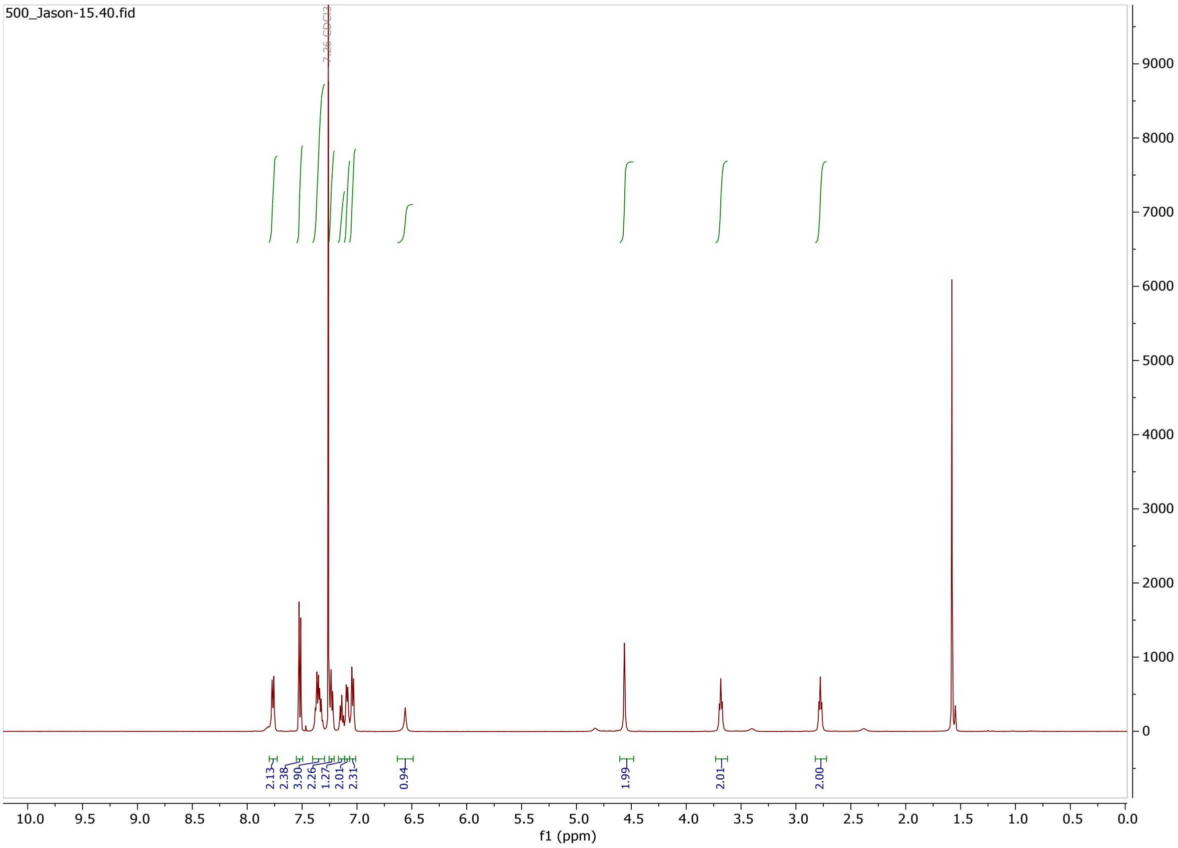

500_Jason-15.40.fid
7.26 CDCl3
2.13
2.38
3.90
2.26
1.27
2.01
2.31
0.94
1.99
2.01
2.00
f1 (ppm)

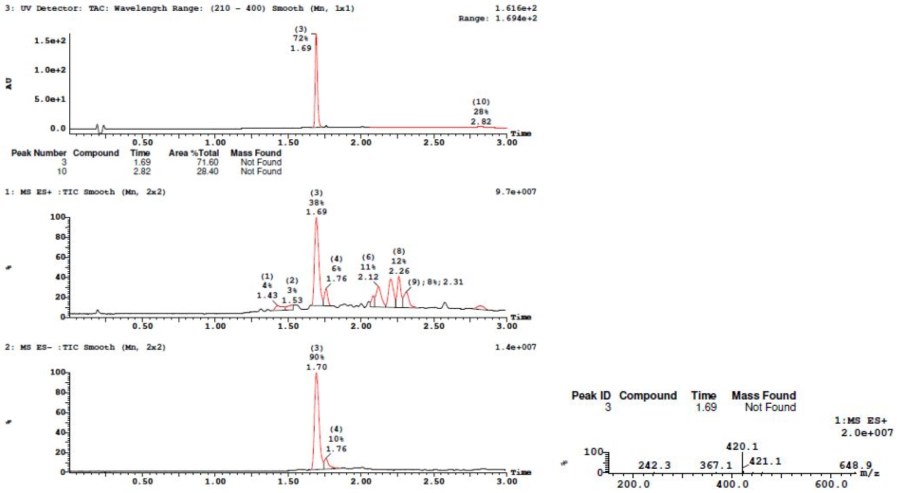

3: UV Detector: TAC: Wavelength Range: (210 - 400) Smooth (Mn, 1x1)
1.616e+2
Range: 1.694e+2
Peak Number Compound Time Area %Total Mass Found
3 1.69 71.60 Not Found
10 2.82 28.40 Not Found
1: MS ES+ :TIC Smooth (Mn, 2x2)
9.7e+007
2: MS ES- :TIC Smooth (Mn, 2x2)
1.4e+007
Peak ID Compound Time Mass Found
3 1.69 Not Found
1:MS ES+
2.0e+007
420.1
421.1
242.3
367.1
648.9
m/z

MDI-60018 (**22**)

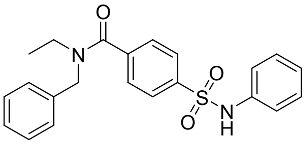

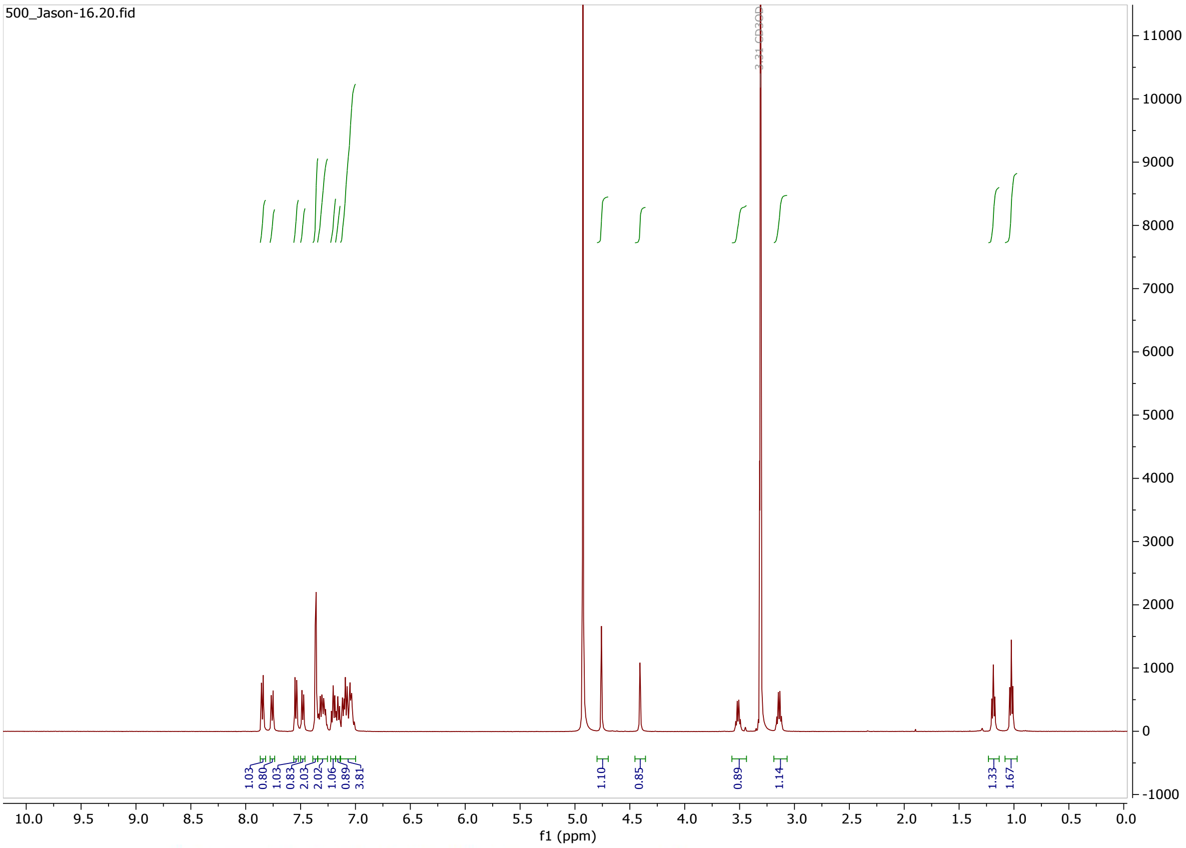

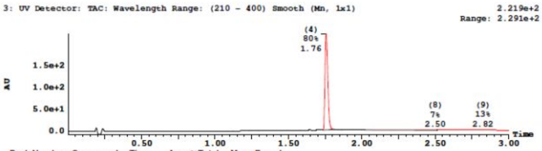

Peak Number	Compound	Time	Area %Total	Mass Found
4		1.76	79.86	Not Found
8		2.50	6.65	Not Found
9		2.82	13.49	Not Found

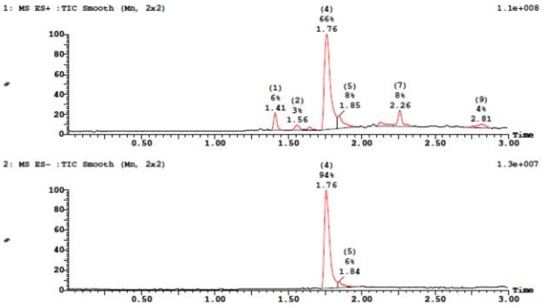

Peak ID	Compound	Time	Mass Found
5		1.85	Not Found

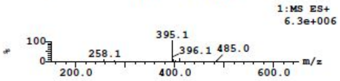

MDI-60052 (**23**)

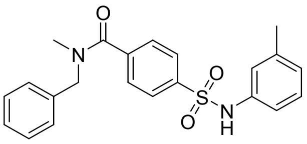

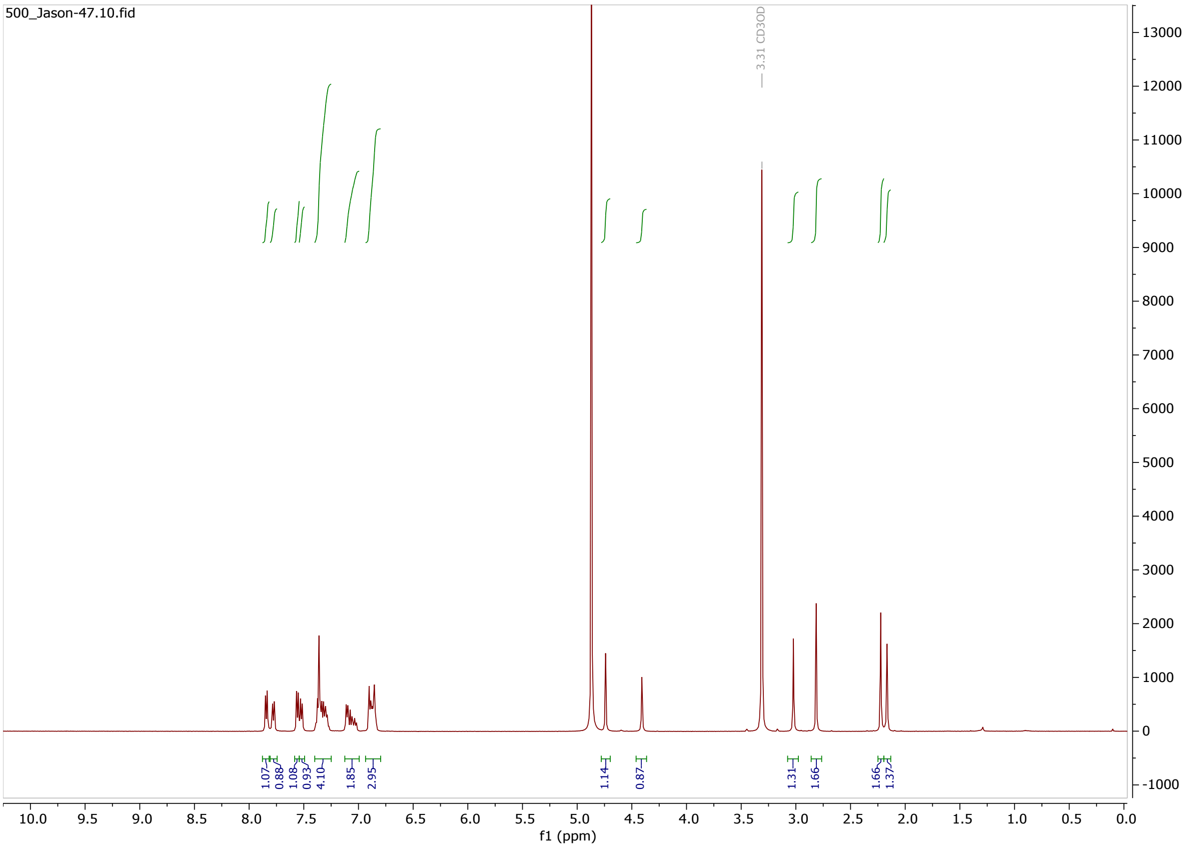

500_Jason-47.10.fid
3.31 CD3OD
1.07
0.88
1.08
0.93
4.10
1.85
2.95
1.14
0.87
1.31
1.66
1.66
1.37
f1 (ppm)

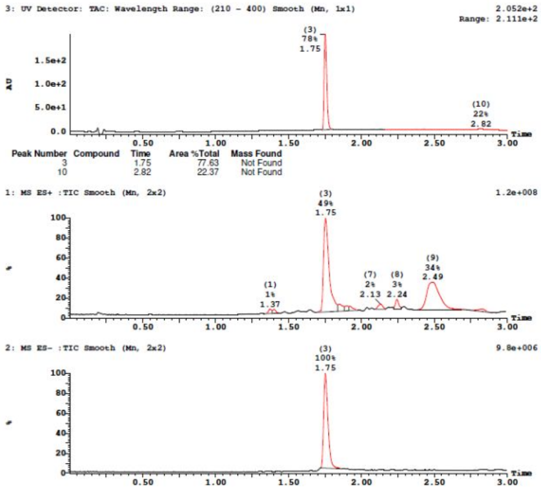

3: UV Detector: TAC: Wavelength Range: (210 - 400) Smooth (Mn, 1x1)
2.052e+2
Range: 2.111e+2
(3) 78% 1.75
(10) 22% 2.82
Peak Number Compound Time Area %Total Mass Found
3 1.75 77.63 Not Found
10 2.82 22.37 Not Found
1: MS ES+ :TIC Smooth (Mn, 2x2)
1.2e+008
(3) 49% 1.75
(1) 1% 1.37
(7) 2% 2.13
(8) 3% 2.24
(9) 34% 2.49
2: MS ES- :TIC Smooth (Mn, 2x2)
9.8e+006
(3) 100% 1.75

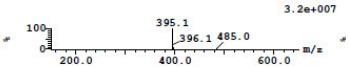

Peak ID Compound Time Mass Found
3 1.75 Not Found
1:MS ES+
3.2e+007
395.1
396.1
485.0
m/z

MDI-60047 (**24**)

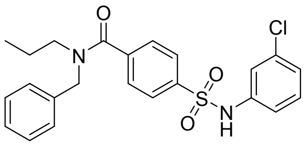
O
N
Cl
O
S
O
N
H

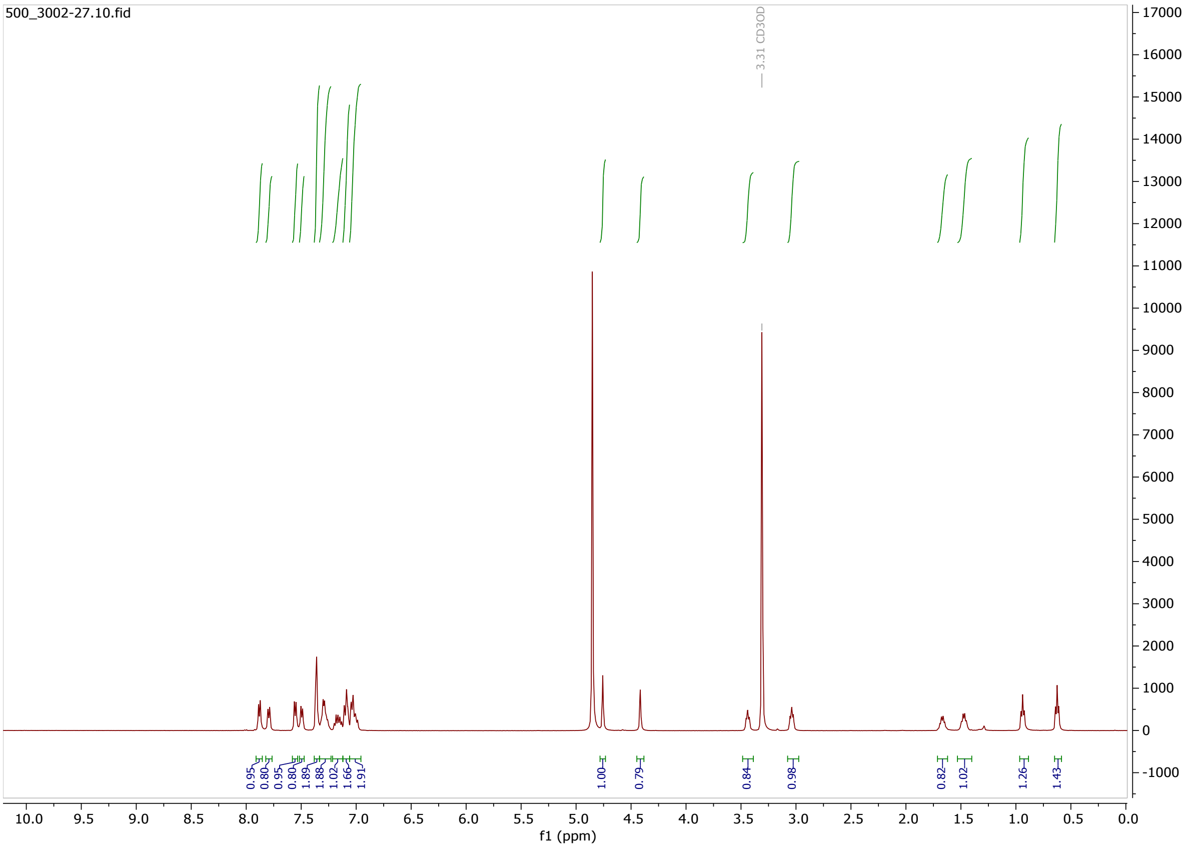
500_3002-27.10.fid
3.31 CD3OD
0.95
0.80
0.95
0.80
1.89
1.88
1.02
1.66
1.91
1.00
0.79
0.84
0.98
0.82
1.02
1.26
1.43
f1 (ppm)

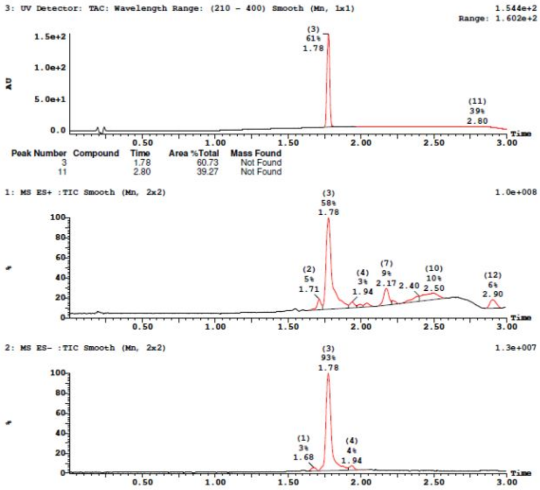
3: UV Detector: TAC: Wavelength Range: (210 - 400) Smooth (Mn, 1x1)
1.544e+2
Range: 1.602e+2
AU
Peak Number Compound Time Area %Total Mass Found
3 1.78 60.73 Not Found
11 2.80 39.27 Not Found
1: MS ES+ :TIC Smooth (Mn, 2x2)
1.0e+008
2: MS ES- :TIC Smooth (Mn, 2x2)
1.3e+007
Time

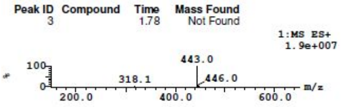
Peak ID Compound Time Mass Found
3 1.78 Not Found
1:MS ES+
1.9e+007
443.0
318.1
446.0
m/z
